# Comparative Metabolomics Identifies Diterpene-Rich Marine Macroalgae with Potent Antiproliferative Activity Against Colorectal Cancer Cells

**DOI:** 10.3390/md24090329

**Published:** 2026-09-21

**Authors:** Ana M. García-Cervantes, Tarik Chileh-Chelh, Rosalía López-Ruiz, Juan J. Gallardo-Rodríguez, José Luis Guil-Guerrero

**Affiliations:** 1 Department of Agronomy, Food Technology División, University of Almeria, 04120 Almeria, Spain; agc874@ual.es (A.M.G.-C.); ct506@ual.es (T.C.-C.); 2 Department of Chemistry-Physics, Analytical Chemistry of Contaminants, University of Almeria, 04120 Almeria, Spain; rosalialr@ual.es; 3 Department of Chemical Engineering, University of Almeria, 04120 Almeria, Spain; jgr285@ual.es

**Keywords:** *Dictyota dichotoma*, *Rugulopteryx okamurae*, LC-MS untargeted metabolomics, cancer cell cultures, reactive oxygen species (ROS)

## Abstract

Marine macroalgae constitutes an important source of structurally diverse secondary metabolites with significant pharmaceutical potential. In the present study, an untargeted liquid chromatography–mass spectrometry (LC-MS)-based metabolomic approach was integrated with biological screening to investigate the relationship between metabolite composition and antiproliferative activity in eight Mediterranean macroalgal species. Crude organic extracts were evaluated against the human colorectal cancer cell lines HT-29 and HCT-116, while the most active extracts were further investigated by flow cytometry to explore their cellular effects. Comparative metabolomic analysis revealed marked interspecific differences, with the brown algae *Dictyota dichotoma* and *Rugulopteryx okamurae* exhibiting metabolomes enriched in lipophilic diterpenoids and showing the highest cytotoxic activity. Although the bioactive compounds were not isolated, the antiproliferative effects were associated with diterpene-rich metabolite profiles containing tentatively annotated compounds such as dictyodial- and dilkamural-related diterpenoids tentatively. *D. dichotoma* displayed potent activity against both HT-29 and HCT-116 cells (GI_50_ ≈ 4.5–5 μg mL^−1^), whereas *R. okamurae* was markedly more active against HT-29 than HCT-116 cells, demonstrating that tumour genetic background substantially influences cellular susceptibility to macroalgal extracts and highlighting the importance of evaluating multiple cancer cell models during preliminary screening. Flow cytometry demonstrated increased intracellular reactive oxygen species (ROS), reduced metabolic viability, and decreased cell concentration following treatment with the most active extracts, whereas Annexin V/propidium iodide staining showed no detectable increase in apoptotic cell populations. These findings suggest that oxidative stress-associated metabolic impairment, rather than classical apoptosis, contributes to the observed antiproliferative activity. Overall, this study demonstrates the value of integrating untargeted metabolomics with comparative biological screening to identify promising marine macroalgae and metabolite classes for future bioassay-guided isolation and development of marine-derived anticancer agents.

## 1. Introduction

Macroalgae are rich sources of bioactive compounds with antioxidant, antimicrobial, antitumor, anti-inflammatory, and other biological activities, attracting growing interest because of their potential applications in medicine, biotechnology, nutraceuticals, and industrial processes. They are classified into three major groups based on pigmentation: Rhodophyta (red), Phaeophyta (brown), and Chlorophyta (green) algae.

Red algae are marine organisms characterised by phycoerythrin and phycocyanin. Their cell walls contain cellulose and sulfated polysaccharides such as agar, agarose, and carrageenan, which are widely used in the food, pharmaceutical, and biotechnology industries [1,2]. Brown algae contain chlorophyll a and c together with fucoxanthin, the carotenoid responsible for their characteristic brown colouration. Rich in alginates, laminarin, and fucoidan, they are valuable for food, cosmetic, pharmaceutical, and biofuel applications [1,2,3]. Green algae, containing chlorophyll a and b, inhabit marine and freshwater environments and produce compounds including ulvan, sulfated galactans, and xylans, which have attracted attention because of their potential applications as functional ingredients, dietary supplements, and biofuel feedstocks [1,2,3].

Macroalgae produce a wide range of secondary metabolites with antimicrobial, antioxidant, anti-inflammatory, antidiabetic, and anticancer activities [4,5]. Growing experimental evidence indicates that extracts from taxonomically diverse macroalgal species exert antiproliferative effects against colorectal cancer cells through multiple mechanisms, including oxidative stress modulation, apoptosis induction, and cell-cycle regulation. Among the most relevant studies, hexadecanoic acid from *Turbinaria ornata* inhibited HT-29 cell growth (IC_50_ = 36.04 μg mL^−1^) [6], while extracts from *Sargassum ilicifolium* also showed activity against HT-29 cells [7]. Fucoidan from *Saccharina cichorioides* enhanced the antiproliferative effect of resveratrol in HCT-116 cells [8]. Likewise, ethanol extracts of *Eucheuma* spp. were active against HCT-116 cells, carotenoid-rich extracts from *Neochloris oleoabundans* inhibited several colon cancer cell lines, and isololiolide from *Cystoseira tamariscifolia* induced apoptosis in hepatocarcinoma cells [9,10,11]. Extracts from *Asparagopsis armata* and *Sphaerococcus coronopifolius* also exhibited significant antiproliferative effects in colon cancer models [12]. Collectively, these studies highlight the considerable potential of macroalgae as sources of bioactive compounds for the development of chemopreventive and therapeutic strategies against colorectal and other cancers [13]. Additionally, brown algae have shown significant antioxidant and antiviral properties [14,15].

The biological activities of macroalgae are largely attributed to compounds such as phlorotannins, fucoidan, laminarin, carotenoids, and other polyphenols. The biological activity of macroalgal extracts likely results from the combined, potentially synergistic action of multiple metabolite classes rather than from individual compounds acting in isolation. Phlorotannins exhibit antioxidant, anti-inflammatory, and antitumor properties, whereas fucoidan and laminarin have demonstrated antioxidant, anti-inflammatory, and anticancer effects, with laminarin also showing prebiotic activity [4,16,17]. Fucoxanthin, the predominant carotenoid in brown algae, together with other xanthophylls, possesses antioxidant and anticancer properties, while polyphenols from species such as *Fucus vesiculosus* have shown immunostimulatory effects [5,14].

To further investigate the relationship between chemical composition and biological activity, extracts from *Cystoseira humilis*, *Rugulopteryx okamurae*, *Dictyota dichotoma*, *Ericaria selaginoides*, *Codium bursa*, *Asparagopsis armata*, *Polysiphonia elongata* and *Polysiphonia* spp. collected along different coastal areas of Andalusia (southern Spain) were evaluated for their biological potential. Given the growing interest in marine natural products, macroalgae constitute valuable sources of compounds with promising applications in pharmaceuticals, functional foods, and other biotechnology sectors [15,18,19].

Although numerous macroalgal extracts have shown cytotoxic activity, few comparative studies have systematically integrated untargeted metabolomics with biological activity across multiple macroalgal species to identify metabolite signatures associated with antiproliferative effects. To address this gap, this study aimed to characterise and compare the untargeted LC-MS metabolomic profiles of eight Mediterranean marine macroalgal species and evaluate their targeted cytotoxic activity against colorectal cancer cell lines.

To evaluate interspecific variation across major taxonomic groups, we collected eight abundant macroalgal species representing brown (Phaeophyta), red (Rhodophyta), and green (Chlorophyta) algae along the Andalusian coastline (southern Spain) to compare their metabolomic profiles and bioactivities within a shared regional environment.

Crucially, most existing literature evaluates isolated macroalgal species under varying experimental and geographical conditions, making it difficult to discern whether bioactivity variations stem from intrinsic taxonomic chemical profiles or environmental plasticity. The selection of these eight specific species, comprising native brown taxa (*Cystoseira*, *Ericaria*, *Dictyota*), the invasive brown alga *R. okamurae*, alongside representative red and green macroalgae, spans a highly diverse chemical space ranging from lipophilic diterpenoids to complex halogenated and nitrogenous compounds. Evaluating these co-occurring species within a unified regional framework eliminates environmental confounding factors, enabling a direct, side-by-side comparison to pinpoint the specific secondary metabolite signatures driving selective colorectal cytotoxicity.

**Figure 1:**
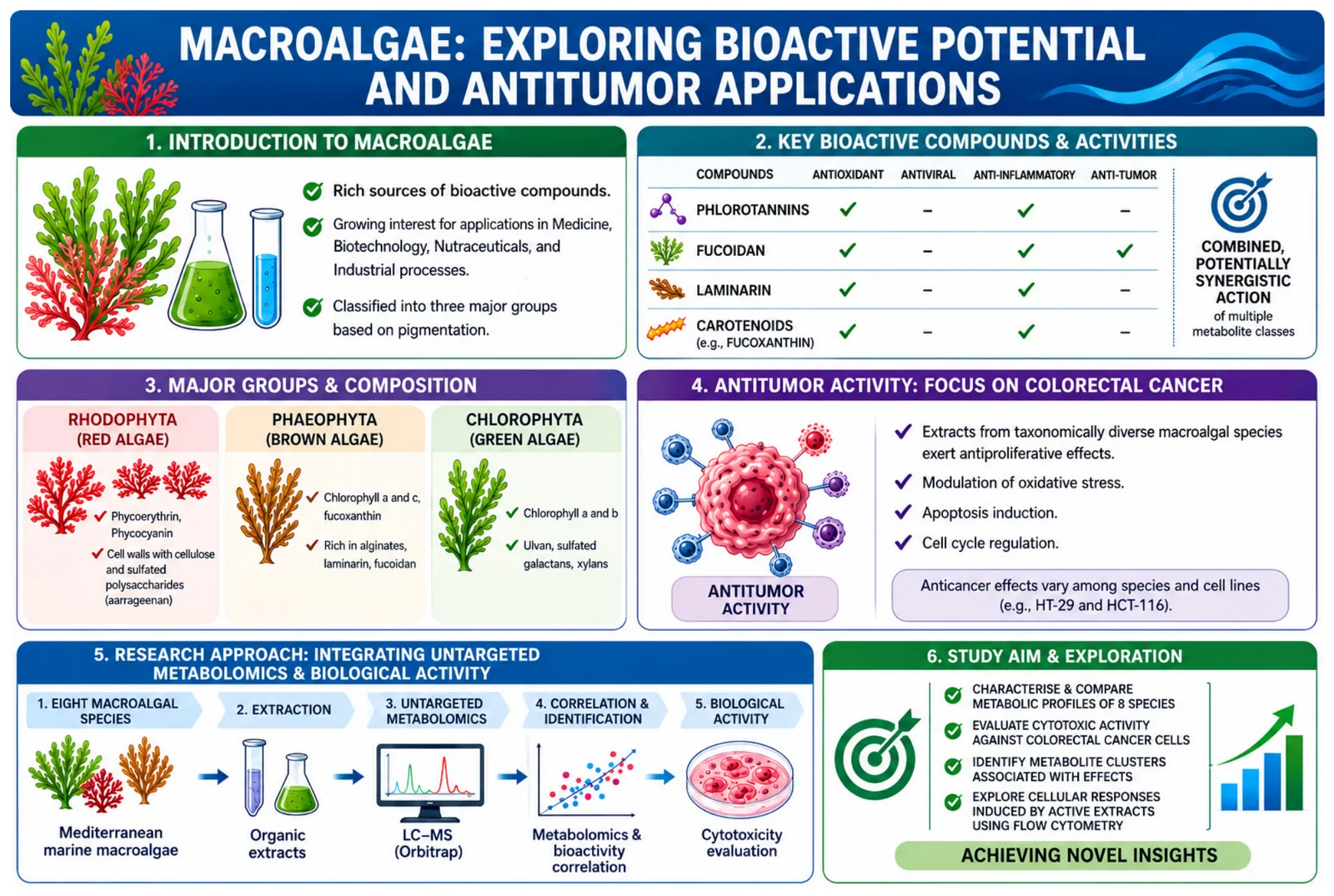
Overview of the study’s framework exploring the bioactive and antitumor potential of marine macroalgae. The schematic outlines the major macroalgal taxonomic groups (Rhodophyta, Phaeophyta, and Chlorophyta) and their key bioactive compounds, contextualising their application in colorectal cancer models. It also illustrates an integrated research workflow—combining organic extraction, untargeted LC-MS metabolomics, and biological cytotoxicity evaluation—designed to identify metabolite clusters associated with antiproliferative cellular responses. Figure generated using ChatGPT (OpenAI, GPT-5.5) with the prompt: “Create a structured 6-panel scientific infographic layout titled ‘MACROALGAE: EXPLORING BIOACTIVE POTENTIAL AND ANTITUMOR APPLICATIONS’ illustrating: (1) Introduction to macroalgae; (2) Key bioactive compound classes and activities; (3) Major macroalgal groups (Rhodophyta, Phaeophyta, Chlorophyta); (4) Antitumor activity mechanisms focused on colorectal cancer cell lines (HT-29, HCT-116); (5) Integrated research workflow (8 Mediterranean species, organic extraction, Orbitrap LC-MS metabolomics, correlation, cytotoxicity evaluation); and (6) Overall study aims.” The authors have reviewed and edited the output and take full responsibility for the content of this publication.

As summarized in Figure 1, our approach integrates these metabolomic correlations with flow cytometry to explore the cellular responses induced by the most active extracts, ultimately aiming to identify promising metabolite clusters for the development of marine-derived anticancer agents.

## 2. Results and Discussion

### 2.1. Metabolomics Overview

#### 2.1.1. Overall Metabolomic Overview

An untargeted liquid chromatography–mass spectrometry (LC-MS) metabolomics of various marine macroalgae provides insight into the chemical diversity and metabolic specialisation of different marine macroalgae. Only metabolites with relative abundances greater than 0.1% were included in the comparative analysis. Their classification into metabolite families is summarised in Table 1, whereas the complete metabolite annotations and relative abundance data are provided in the Supplementary Tables S1–S8 (Supplementary Material). The distribution of metabolite families and their corresponding Metabolomics Standards Initiative (MSI) annotation levels provide valuable insights into species-specific metabolic composition, potential ecological adaptations, and the analytical landscape of marine natural products. Nevertheless, these findings should be interpreted with consideration of the confidence associated with the different MSI annotation levels, which indicate the degree of certainty of each metabolite annotation.

The present metabolomic dataset should be interpreted in light of the extraction protocol’s selectivity. Sequential extraction with dichloromethane and methanol preferentially recovered low- and medium-polarity metabolites, including terpenoids, fatty acids, oxylipins, sterols, pigments, and small nitrogen- and sulfur-containing compounds, whereas highly polar macromolecules are expected to be underrepresented owing to their limited extraction by organic solvents [20,21,22,23]. Furthermore, because the dried extracts were reconstituted in a dichloromethane/acetonitrile (50:50) mixture prior to LC-MS analysis, recovery of the most polar metabolites may have been further limited. Consequently, the metabolomic profiles obtained primarily represent the extractable low- and medium-polarity metabolome rather than the complete chemical composition of each algal species.

This extraction strategy efficiently recovered the lipophilic diterpenoids characteristic of the brown algae *R. okamurae* and *D. dichotoma*, as well as the fatty acids, sterols, meroterpenoids, and other lipid-derived metabolites that predominate in *C. bursa*, *A. armata*, *P. elongata*, and *Polysiphonia* spp. However, highly polar or high-molecular-weight compounds are expected to be underrepresented. These include structural and storage polysaccharides such as alginate, fucoidans, and laminarin in brown algae; sulfated galactans and floridean starch in red algae; sulfated cell wall polysaccharides in *C. bursa*; and polymeric phlorotannins, glycoproteins, and phycobiliproteins, which generally require aqueous extraction procedures. Likewise, highly polar primary metabolites (e.g., soluble sugars, sugar phosphates, organic acids, and many amino acid derivatives) are expected to be underrepresented because of their limited solubility in low-polarity solvent systems and the use of dichloromethane/acetonitrile for sample reconstitution prior to LC-MS analysis, which favours the recovery of more lipophilic metabolites [20,24].

Therefore, the observed interspecific differences should be interpreted in the context of the extraction methodology. While the protocol provides broad coverage of extractable lipophilic and moderately polar metabolites, complementary aqueous extraction approaches would be required to achieve a more comprehensive characterisation of the complete algal metabolome [20,21,22,23].

#### 2.1.2. Species-Specific Metabolomic Profiles

*Cystoseira humilis*

This species displays a metabolome dominated by a combined terpenoid/fatty acid-lipid fraction (31.00%), consistent with the original grouping of terpenoids, fatty acids, oxylipins, and polyols. Sulfur-containing compounds (24.00%) and nitrogenous metabolites (11.00%) are also abundant, whereas halogenated compounds account for only 1.00%. The lipid fraction is characterised by abundant polyunsaturated fatty acids (PUFAs), including arachidonic acid, eicosapentaenoic acid (EPA), linolenic acid, palmitoleic acid, and myristic acid, together with numerous oxylipins and the brown algal storage polyol mannitol. Sulfolipids (SQDG-related compounds), glycolipids, and terpenoids further contribute to this profile, which is consistent with that which has previously been associated with responses to environmental stress of lipid and sulfur metabolism in Phaeophyceae species exposed to environmental stress [25,26] (Table 1, Supplementary Table S1).

*Rugulopteryx okamurae*

The metabolome of *R. okamurae* is dominated by terpenoids, particularly diterpenoids (34.96%; MSI levels 2–3), followed by fatty acids/lipids including oxylipins (11.17%). Minor classes include nitrogenous compounds (3.52%), volatiles (1.48%), and pigments (0.91%). This composition agrees with previous reports describing *R. okamurae* as rich in terpenoid and lipid metabolites. The diterpenoid fraction consists predominantly of spatane- and secospatane-type diterpenoids, including mono-, di-, and tri-oxygenated derivatives, together with characteristic compounds such as 4β-acetoxydictyodial A, Rugukadiol A, and Rugukamural C, which were previously reported as major defensive secondary metabolites of the species. The lipid fraction is characterised by abundant polyunsaturated fatty acids (PUFAs), including arachidonic acid, eicosapentaenoic acid (EPA), oleic acid, palmitic acid, and myristic acid [26,27]. Characteristic low-abundance metabolites (<0.1%) include rugukamurals A–B, okaspatols A–D, and oxygenated carotenoid derivatives related to fucoxanthin, violaxanthin, and neoxanthin (Table 1, Supplementary Table S2).

*Dictyota dichotoma*

*D. dichotoma* exhibited the highest terpenoid content among all analysed species (47.44%), comprising mainly diterpenoids (43.49%) and smaller amounts of sesquiterpenoids/norisoprenoids (3.95%). Additional metabolites included fatty acids (7.54%), phenolics (2.41%), and nitrogenous compounds (0.60%). This diterpene-rich profile is characteristic of the genus *Dictyota*, a recognised source of bioactive diterpenes, several of which have demonstrated cytotoxic, antiviral, and ecological defensive activities. The lipid fraction is characterised by abundant PUFAs, including arachidonic acid, eicosapentaenoic acid (EPA), oleic acid, palmitic acid, and myristic acid [26,28,29]. Representative metabolites include dictyolactam acetate, acetoxyditerpenes, and numerous oxygenated diterpene derivatives that are previously implicated in chemical defence against herbivores and fouling organisms. Minor constituents (<0.1%) included pachydictyol A, dityol E, oxygenated xanthophylls, oxidised sterols, and sulfated fucosterol derivatives (Table 1, Supplementary Table S3).

*Ericaria selaginoides*

The metabolome of *E. selaginoides* is characterised by a high abundance of nitrogenous compounds (30.0%) and terpenoids (27.0%), the latter including oxylipins. Sulfur compounds account for 5.00%, while fatty acids and phenolics each represent approximately 2.0%. The lipid fraction is characterised by PUFA derivatives, including stearidonic acid, palmitoleic acid, and myristic acid derivatives. The nitrogen-containing fraction includes diverse polyamines, indole derivatives, and alkaloid-like metabolites, whereas the lipid fraction comprises fatty acids and oxylipins involved in stress signalling and defence. This metabolite distribution is consistent with previous reports showing that nitrogen-containing metabolites and polyphenols in this species vary according to seasonal and environmental conditions [26,30] (Table 1, Supplementary Table S4).

*Codium bursa*

The metabolome of *C. bursa* is largely composed of terpenoids (38.65%) and fatty acids/lipids (25.23%), including phospholipids (3.84%). Minor metabolite classes comprise pigments (4.50%), nitrogenous compounds (3.50%), volatiles (1.33%), and phenolics (0.85%). The terpenoid fraction is characterised by meroterpenoids, including Brevione A and related derivatives, while the lipid fraction contains abundant PUFAs such as palmitic, oleic, linolenic, and palmitelaidic acids. This composition is consistent with previous reports describing metabolites involved in chemical defence and environmental adaptation [26,31]. Low-abundance metabolites (<0.1%) include siphonaxanthin, dimethylsulfoniopropionate, oxygenated clerosterol and codisterol derivatives, together with oxysterols and polyoxygenated sterols (Table 1, Supplementary Table S5).

*Asparagopsis armata*

*A. armata* exhibits a chemically diverse metabolome, dominated by fatty acids/lipids (17.09%), nitrogenous compounds (12.48%), terpenoids (9.53%), oxylipins (9.15%), phenolic/polyketide metabolites (7.51%), and pigments (3.40%). The lipid fraction is characterised by abundant PUFAs, such as palmitic, oleic, and palmitelaidic acids, along with numerous oxidised fatty acids, including dihydroxyhexadecanoic acid derivatives. Additional meroterpenoids and carotenoid-derived metabolites further contribute to the chemical diversity of this species, which is consistent with the previously reported antioxidant and antimicrobial properties of this species [26,27]. Characteristic minor constituents (<0.1%) include 9,10-epoxy-12-octadecanoic acid, oxidised sterols, sulfated sterols, sterol glycosides, and oxygenated carotenoid derivatives (Table 1, Supplementary Table S6).

*Polysiphonia elongata*

*P. elongata* exhibits a relatively balanced metabolomic profile, with fatty acids/lipids (16.14%), terpenoids (15.20%), nitrogenous compounds (10.48%), phenolics (4.72%), and oxylipins (3.45%) as the major classes. The predominant lipid metabolites include palmitic, oleic, myristic, and palmitelaidic acids, together with abundant oxylipins such as hydroxyeicosapentaenoic acid (HEPE) and epoxystearic acid derivatives. This chemical composition is consistent with the accumulation of nitrogen-containing metabolites and photoprotective compounds characteristic of filamentous red algae previously described in filamentous red algae exposed to variable light environments [26,32] (Table 1, Supplementary Table S7).

*Polysiphonia* spp.

In contrast, *Polysiphonia* spp. is dominated by fatty acids and lipids (51.00%), including oxylipins, followed by nitrogenous compounds (23.00%) and terpenoids (11.00%), encompassing phenolic metabolites. Sulfur (5.00%) and halogenated compounds (2.00%) are present in lower proportions. The lipid fraction contains abundant oleic and palmitic acids, together with oxygenated fatty acids and polyoxygenated polyketides, suggesting an active oxidative lipid metabolism. This metabolomic profile is consistent with adaptive strategies reported for the genus, where lipid remodelling and halogenated metabolites have been proposed to contribute to membrane stability and protection against biofouling [33,34] (Table 1, Supplementary Table S8).

Overall, while species such as *P. elongata*, *C. bursa*, and *Polysiphonia* spp. present metabolomes dominated by primary lipids, phenolics, and photoprotective compounds, the distinct diterpenoid enrichment observed in *D. dichotoma* and *R. okamurae* establishes the primary chemical basis for their bioactivity. Subsequently, we present the direct correlation between these species-specific metabolomic signatures, their cytotoxic potency against HCT-116 and HT-29 cells, and the resulting Selectivity Indices (SI).

**Figure 2:**
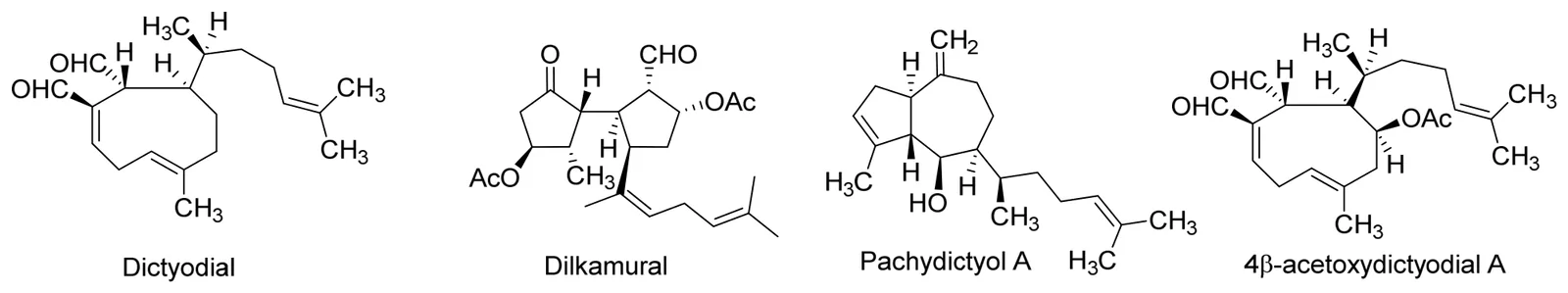
Chemical structures of key diterpenoids.

To illustrate the structural diversity of the key secondary metabolites identified in the algae, the chemical structures of representative diterpenoids—including dictyodial, dilkamural, pachydictyol A, and 4β-acetoxydictyodial A—are depicted in Figure 2. These skeletons represent the primary chemical features associated with the biological profiles observed in these extracts.

#### 2.1.3. Heatmap Analysis of Algal Chemodiversity

**Figure 3:**
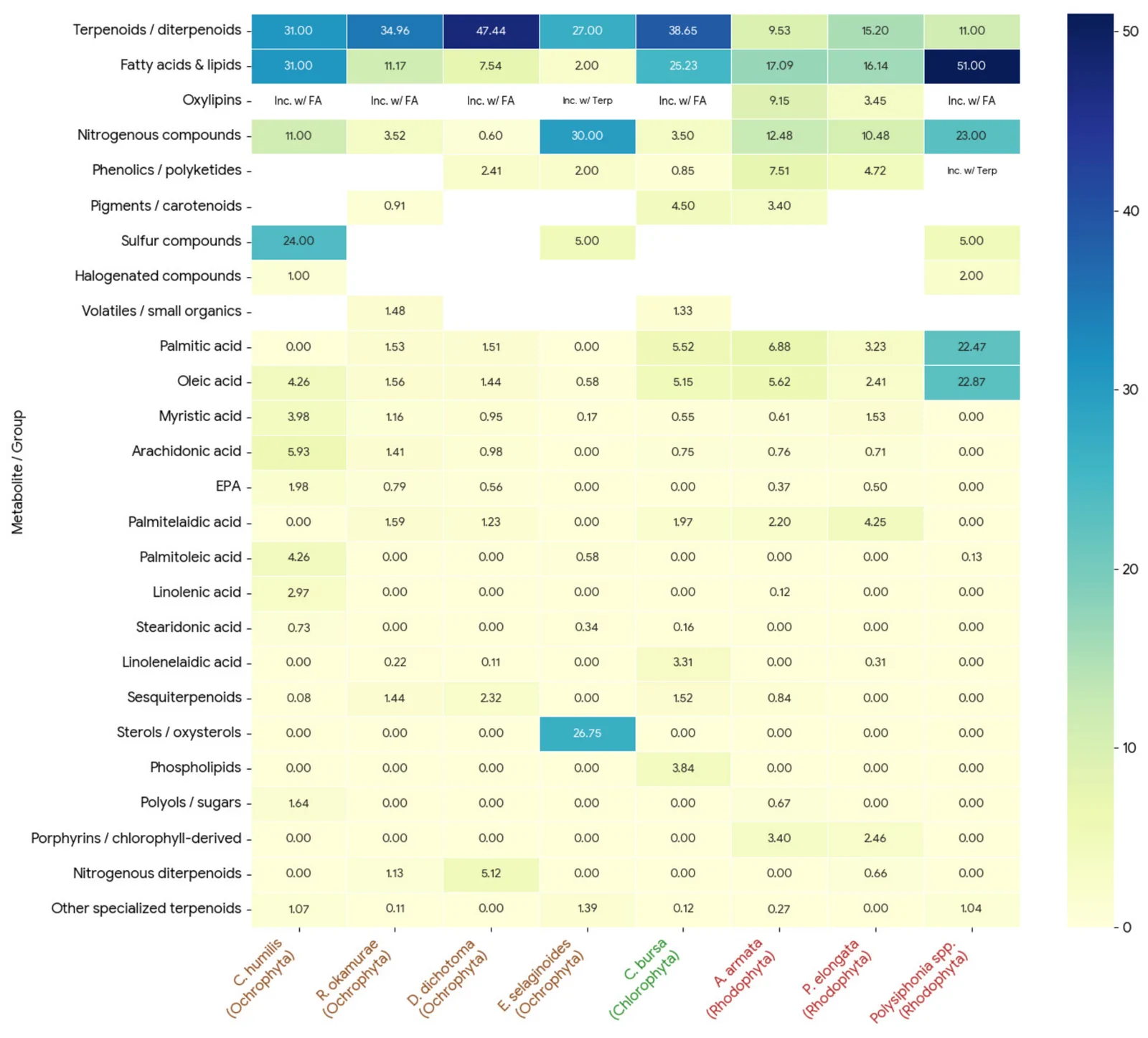
Quantitative profile of targeted metabolites in selected macroalgae species categorised by phylum. Heatmap displaying the distribution of major metabolite groups and specific compounds across eight macroalgal species. Species on the x-axis are grouped and colour-coded by their respective phyla: Ochrophyta (brown), Chlorophyta (green), and Rhodophyta (red). The colour scale reflects the relative concentration or abundance of each metabolite, with darker blue indicating higher values and pale yellow indicating zero or trace amounts. Blank cells containing text indicate that the specific metabolite group was quantified within a broader category (“Inc. w/FA” = Included with Fatty acids & lipids; “Inc. w/Terp” = Included with Terpenoids).

The quantitative analysis of the algal metabolome reveals distinct, phylum-specific chemical signatures, most notably in the distribution of terpenoids and lipids (Figure 3). Terpenoids and diterpenoids dominate the metabolic profiles of the Ochrophyta species, reaching their maximum concentration in *D. dichotoma* (47.44), while also representing a primary component of the Chlorophyta species *C. bursa* (38.65) (Figure 3).

Conversely, lipid accumulation is most pronounced within the Rhodophyta phylum. *Polysiphonia* spp. exhibits the highest total fatty acid and lipid concentration (51.00) across the dataset. This elevated lipid profile is driven almost entirely by exceptional levels of palmitic acid (22.47) and oleic acid (22.87), which are minimal in the Ochrophyta species (Figure 3).

Secondary metabolite production also demonstrates extreme species-specific specialisation. Nitrogenous compounds peak significantly in the Ochrophyte *E. selaginoides* (30.00), which otherwise lacks notable fatty acid or sulfur concentrations. Meanwhile, *C. humilis* shows a unique prevalence of sulfur compounds (24.00) that are either absent or negligible in the rest of the surveyed algae (Figure 3).

### 2.2. Antitumor Cell Activity

**Figure 4:**
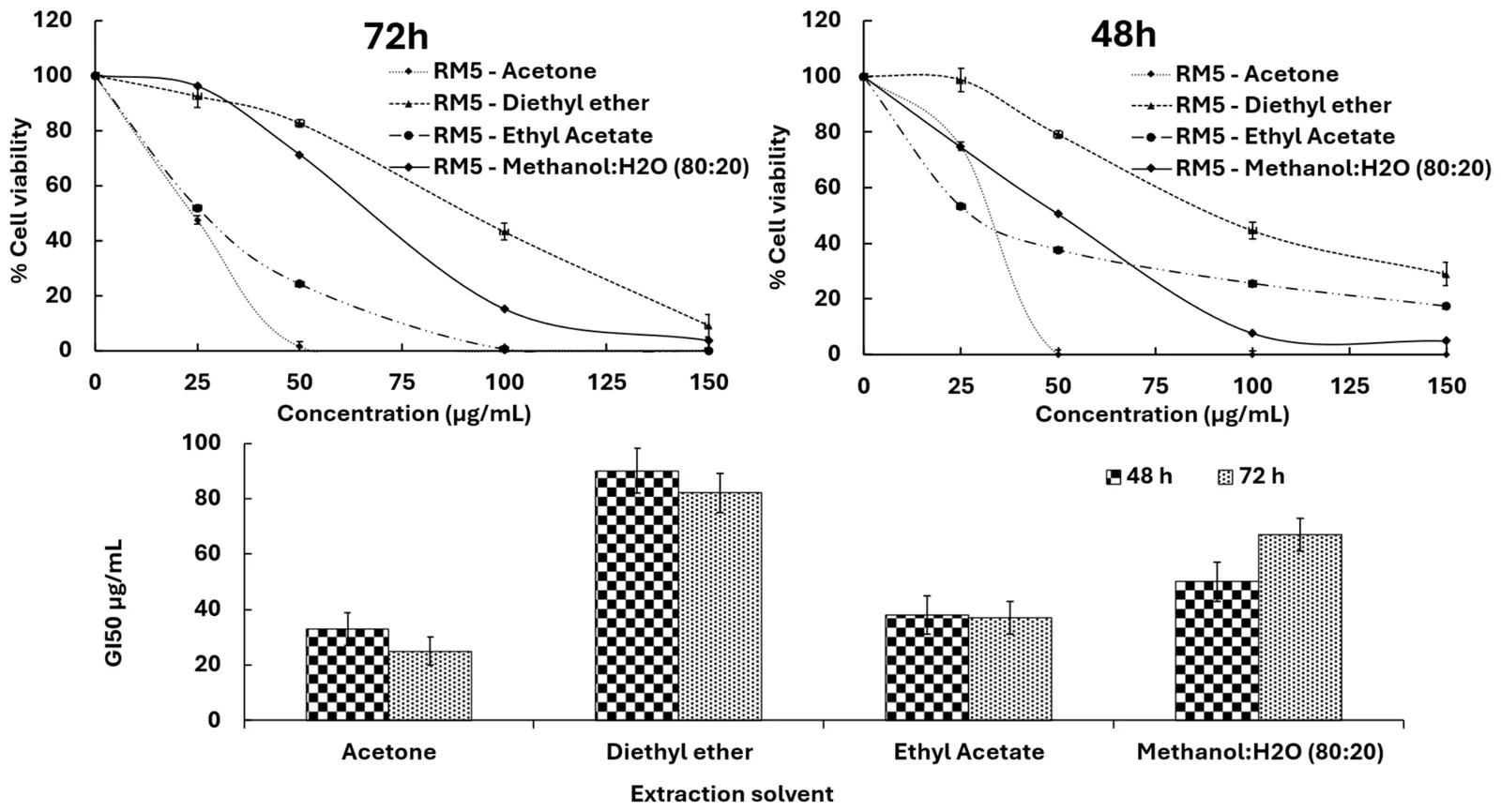
Cytotoxic potential of *R. okamurae* against HT-29 in different solvents. Error bars represent SD (*n* = 3).

The preliminary solvent screening revealed that the cytotoxic activity of *R. okamurae* against HT-29 colon cancer cells was strongly influenced by extraction solvent, suggesting that the principal bioactive metabolites are predominantly lipophilic (Figure 4). The acetone extract exhibited the highest antiproliferative activity (GI_50_ = 25 ± 4 µg mL^−1^ at 72 h), followed by the diethyl ether extract (GI_50_ = 82 ± 7 µg mL^−1^), consistent with the efficient extraction of the alga’s abundant diterpenoid fraction (~34%), including secospatane-type compounds such as dilkamural. These results suggest that diterpenoids may contribute substantially to the observed cytotoxicity.

In contrast, the ethyl acetate extract showed the lowest activity (GI_50_ = 37 ± 6 µg mL^−1^ at 72 h). The aqueous methanol (80:20) extract exhibited a time-dependent increase in cytotoxicity, indicating that more polar metabolites or partially soluble bioactive compounds may require longer exposure times to accumulate intracellularly and exert measurable antiproliferative effects. Overall, these findings demonstrate that extraction solvent polarity markedly influences the recovery of cytotoxic constituents from *R. okamurae*, with non-polar and moderate polar solvents providing the greatest enrichment of bioactive compounds. This trend is consistent with previous observations in *D. dichotoma*, where the chloroform and petroleum ether fractions exhibited the strongest cytotoxic activity, supporting the hypothesis that lipophilic secondary metabolites are primarily responsible for the anticancer properties of brown algae [35].

**Figure 5:**
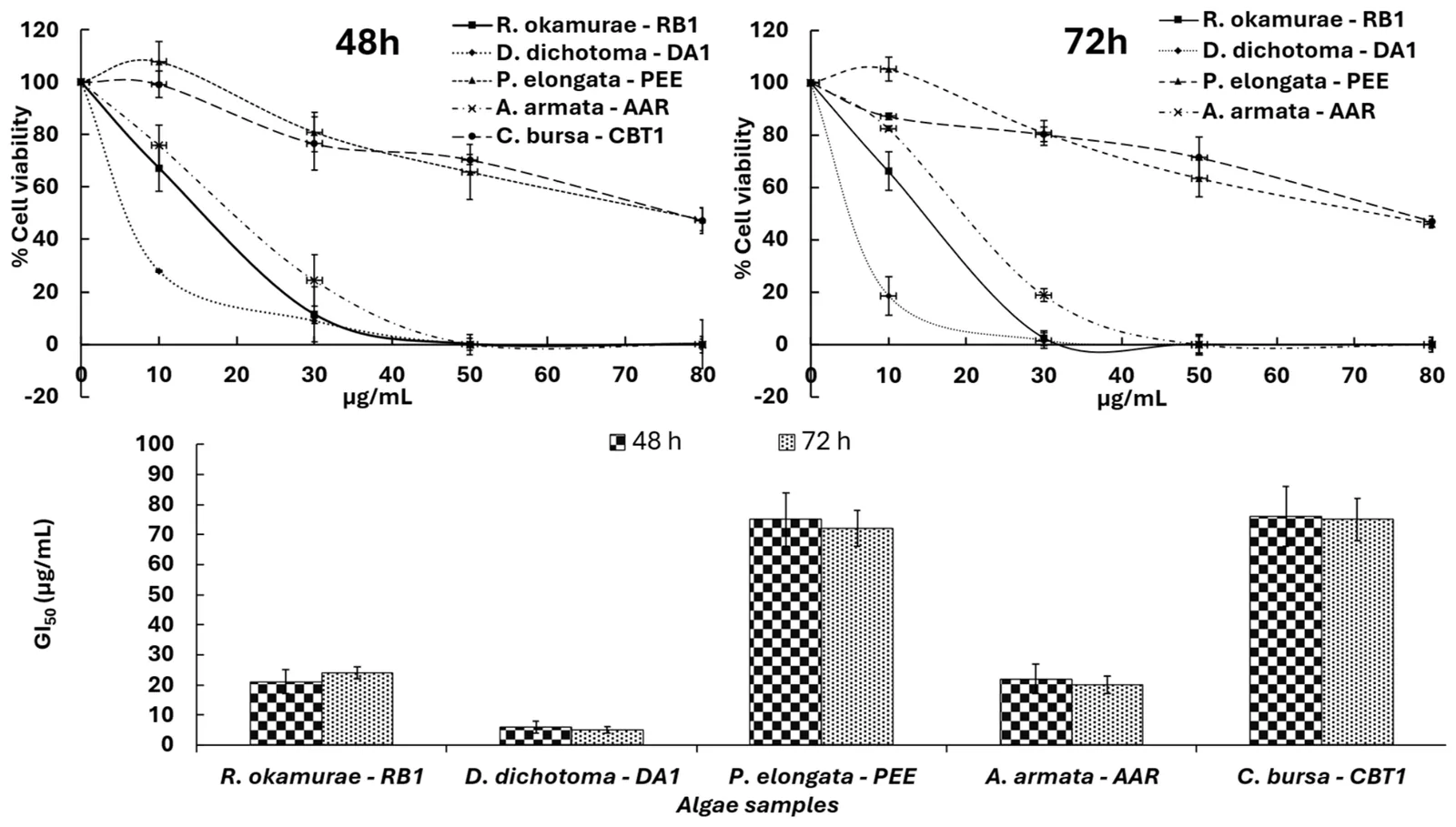
Cytotoxic effects of acetone extracts from selected marine macroalgae on HT-29 colon cancer cells assessed by the MTT assay. Dose–response curves (48 and 72 h) and corresponding GI_50_ values are shown. Error bars represent SD (*n* = 3).

The evaluation of the acetone extracts against the HT-29 colon cancer cell line reveals a correlation between the abundance of metabolites previously associated with ecological defence and the observed antiproliferative activity. The bioassay results display a clear concentration- and time-dependent cytotoxic effect, with GI_50_ values at 72 h highlighting distinct tiers of bioactivity that closely correspond to the relative abundance of specific secondary metabolite families (Figure 5).

The strongest cytotoxic activity against HT-29 cells was observed for the brown algae *D. dichotoma* (GI_50_ = 5 ± 1 µg mL^−1^ at 72 h) and *R. okamurae* (GI_50_ = 24 ± 2 µg mL^−1^ at 72 h). Their metabolomic profiles plausibly explain this activity, given that both species contain a high abundance of diterpenoids. In *D. dichotoma*, diterpenes accounted for 47% of the metabolome and included highly oxidised compounds such as dictyodial and pachydictyol A. These lipophilic metabolites, known for their ecological roles as antifoulants and herbivore deterrents, have previously been associated with membrane disruption and oxidative stress and have repeatedly been proposed among the principal contributors to the cytotoxic activity of *D. dichotoma*.

Likewise, the activity of *R. okamurae* correlated with its high diterpenoid content (35%; MSI Level 3), particularly secospatane-type diterpenoids such as dilkamural. These compounds, previously linked to the species’ invasive success, also appear to possess potent antiproliferative properties. The different responses observed between HT-29 and HCT-116 cells suggest cell line-specific susceptibility, indicating that the molecular targets or resistance mechanisms differ between these colorectal cancer models. Similar cancer cell-specific responses have previously been reported for *D. dichotoma*, whose extracts displayed differential cytotoxicity depending on the tumour cell line evaluated.

*A. armata* demonstrated moderate-to-strong antiproliferative activity against HT-29 colon cancer cells, exhibiting a GI_50_ value of 20 ± 3 µg mL^−1^ at 72 h (Figure 5). This bioactivity strongly aligns with its specialised metabolomic profile, which is notably enriched in fatty acids (17.09%), oxylipins (9.15%), and halogenated or nitrogenous compounds (12.48%). These results are consistent with published literature benchmarks, where non-polar extracts of *A. armata* consistently display high cytotoxic potency against human colorectal models, typically yielding IC_50_ values between 10 and 35 µg mL^−1^ [36]. Their lipophilic character may facilitate cellular uptake, and previous studies have associated these compounds with increased ROS production and activation of apoptotic pathways [36,37]. However, the flow cytometry results obtained in the present study suggest that alternative, predominantly non-apoptotic mechanisms may contribute to the observed cytotoxicity (Section 2.3).

Conversely, *C. bursa* and *P. elongata* displayed substantially weaker antiproliferative effects against HT-29 cells, returning GI_50_ values of 75 ± 7 µg mL^−1^ and 72 ± 6 µg mL^−1^ at 72 h, respectively (Figure 5). These experimental outcomes mirror broad marine screening studies, which generally classify Chlorophyta species such as *Codium* spp. and non-bromophenol-enriched Rhodophyta such as *Polysiphonia* spp. as exhibiting low direct cytotoxicity toward mammalian cancer models, frequently reporting IC_50_ values above 50–100 µg mL^−1^ [38,39].

The lower antiproliferative activity may be related, at least in part, to their metabolomic composition. Metabolomic analysis revealed that *C. bursa* is primarily composed of phytol derivatives and aliphatic lipid fractions (38.65% terpenoids and 25.23% lipids), while *P. elongata* lacked highly reactive secondary metabolites such as oxylipins or oxidised diterpenes. Unlike the electrophilic dialdehydes in *Dictyota* or haloforms in *Asparagopsis*, saturated aliphatic hydrocarbons, phytol derivatives, and generic fatty acids are less chemically reactive than necessary to induce acute cell disruption or apoptosis. Consequently, the literature prioritises *Codium* and *Polysiphonia* species for antioxidant, photoprotective, and cosmeceutical applications rather than systemic antineoplastic therapeutics [38,40].

**Figure 6:**
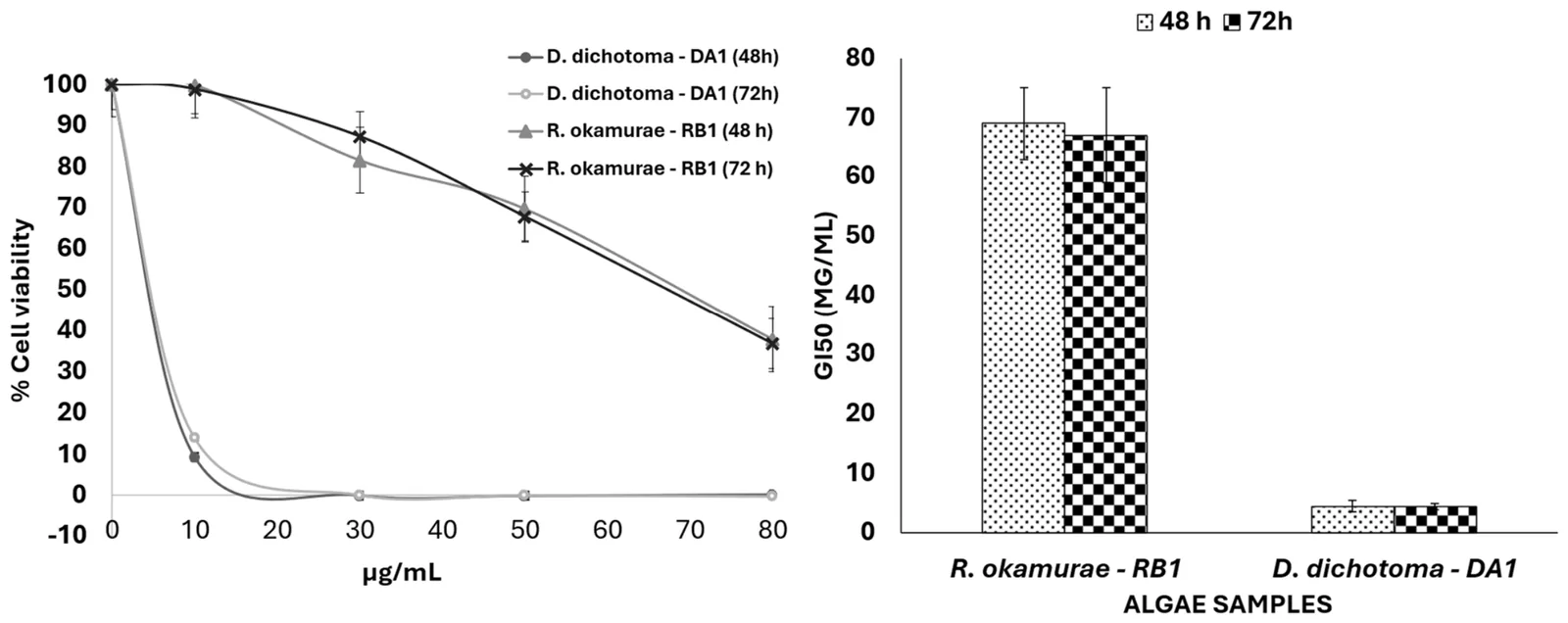
Comparative antiproliferative activity of *D. dichotoma* (DA1) and *R. okamurae* (RB1) acetone extracts against HCT-116 colon cancer cells. Dose–response curves (48 and 72 h) and GI_50_ values are shown. Error bars represent SD (*n* = 3).

Following the initial screening of all macroalgal extracts against HT-29 cells, the acetone extracts of *D. dichotoma* (DA1) and *R. okamurae* (RB1), which exhibited the strongest antiproliferative activity, were selected for further evaluation in HCT-116 cells. HT-29 cells were used for the initial screening (Figure 4 and Figure 5) because they represent a well-established colorectal cancer model for assessing the cytotoxic potential of natural products and allowed a direct comparison of all macroalgal extracts under identical experimental conditions. Preliminary MTT experiments performed in our laboratory demonstrated comparable overall sensitivity patterns between HT-29 and HCT-116 cells, supporting the use of HT-29 as the primary screening model. Subsequently, HCT-116 cells were selected for the detailed evaluation of the two most active extracts (Figure 6) to determine whether their antiproliferative effects were maintained across colorectal cancer cell lines with distinct molecular characteristics and genetic backgrounds. This sequential approach enabled the assessment of both general cytotoxic activity and cell line-specific differences in sensitivity while avoiding unnecessary duplication of the complete screening experiments.

The results revealed marked differences in antiproliferative activity between the two diterpene-rich species, demonstrating that diterpene abundance alone does not determine cytotoxic potency and that metabolite composition and cancer cell susceptibility are also critical factors (Figure 6).

In HCT-116 cancer cells, *D. dichotoma* displayed very strong cytotoxic activity, yielding an identical GI_50_ value of 4.5 ± 0.1 µg mL^−1^ at both 48 and 72 h. This time-independent behaviour indicates that maximal growth inhibition is reached within the initial 48 h exposure window. While an invariant GI_50_ can indicate a rapid onset of cytotoxic action, the absence of marked apoptotic induction in flow cytometry points toward early-onset cytostasis, such as sustained cell cycle arrest, rather than acute apoptotic cell death. Lipophilic diterpenoids in *D. dichotoma* extracts may rapidly permeate cell membranes to engage intracellular targets that halt cell cycle progression early, preventing further cell proliferation without immediately triggering apoptotic cascades. Previous studies likewise identified *D. dichotoma* as highly active against HCT-116 cells, with the greatest activity found in low-polarity fractions such as chloroform and petroleum ether, supporting the importance of lipophilic metabolites in its antiproliferative activity.

Conversely, *R. okamurae* exhibited a drastically reduced, moderate-to-low antiproliferative effect against this cell line, with stable GI_50_ values of 69 ± 6 µg mL^−1^ (48 h) and 67 ± 8 µg mL^−1^ (72 h). This represents a marked drop in potency compared to its performance against the HT-29 cell line (where GI_50_ values ranged from 24 to 25 µg mL^−1^), further highlighting that colorectal cancer cell lines differ substantially in their susceptibility to diterpene-rich algal extracts.

To evaluate the therapeutic safety margin of the extracts against aggressive colon cancer, cytotoxicity was assessed individually in the non-tumoral human colon epithelial cell line CCD 841 CoN. *D. dichotoma* displayed a non-tumoral baseline GI_50_ of 85.2 ± 5.6 μg mL^−1^, whereas *R. okamurae* exhibited a baseline GI_50_ of 85.2 ± 6.1 μg mL^−1^. Using these species-specific baseline values, the Selectivity Index (SI = GI_50_ [CCD 841 CoN]/GI_50_ [cancer line]) revealed marked differences in therapeutic window between the two macroalgae *D. dichotoma* exhibited an exceptional selectivity profile against HCT-116 cells with an SI of 18.9 (GI_50_ = 4.5 ± 0.1 µg mL^−1^ against HCT-116), vastly exceeding the recognised benchmark for high preferential cytotoxicity (SI > 2.0) [41] and demonstrating a broad therapeutic window. Conversely, *R. okamurae* displayed modest-to-moderate selectivity depending on the target line, with an SI of 1.27 (GI_50_ = 67 ± 8 µg mL^−1^) against HCT-116 and a SI of 3.54 (GI_50_ = 24 ± 2 µg mL^−1^) against HT-29, confirming that its safety margin is strongly tied to tumor cell susceptibility. Because its GI_50_ values were markedly lower than those of the rest of the dataset, its data were excluded from the primary graphic representation to maintain visual scale readability. At 72 h of exposure, doxorubicin displayed GI_50_ values of 1.9 ± 0.1 µg mL^−1^ in HCT-116 colon cancer cells and 2.3 ± 0.2 µg mL^−1^ in HT-29 colon cancer cells. When calculated against non-tumorigenic healthy colon cells (CCD 841 CoN), the resulting SI values were 1.6 for HCT116 and 0.9 for HT-29 cells. Although doxorubicin exhibited high antiproliferative potency, the SI of 0.9 in HT-29 cells highlights significant off-target toxicity, confirming that it exerts comparable or even greater cytotoxic damage to non-tumorigenic healthy colon cells than to the malignant cells.

These findings are consistent with those of El-Shaibany et al. [35], who reported strong cytotoxic activity of *D. dichotoma* against HCT-116 and several other human cancer cell lines, with the greatest activity observed in low-polarity fractions such as chloroform and petroleum ether. However, by integrating metabolomic profiling with bioactivity assays, the present study extends previous studies by directly integrating untargeted metabolomics with biological assays, thereby providing evidence that diterpenoid-rich metabolomes are associated with enhanced antiproliferative activity while also demonstrating that the specific metabolite composition, rather than diterpene abundance alone, influences the response of different colorectal cancer cell lines.

HT-29 and HCT-116 cells differ substantially in their molecular background, including p53 status, APC mutations, β-catenin signalling, differentiation state, and oxidative stress responses [42,43]. These biological differences may influence the uptake, metabolism, or intracellular targets of diterpenoid-rich extracts, thereby contributing to the distinct sensitivities observed in the present study [44]. Specifically, the lipophilic nature of diterpenoids suggests that variations in membrane transporter expression between the differentiated HT-29 and the poorly differentiated HCT-116 cells could modulate initial drug availability [45]. Furthermore, the downstream activation of apoptosis or antioxidant defences might be heavily dictated by their divergent p53 and β-catenin mutational statuses [46].

Although the precise mechanisms remain to be elucidated, these findings highlight the importance of evaluating marine natural products across multiple tumour models rather than relying on a single cell line.

### 2.3. Flow Cytometry

**Figure 7:**
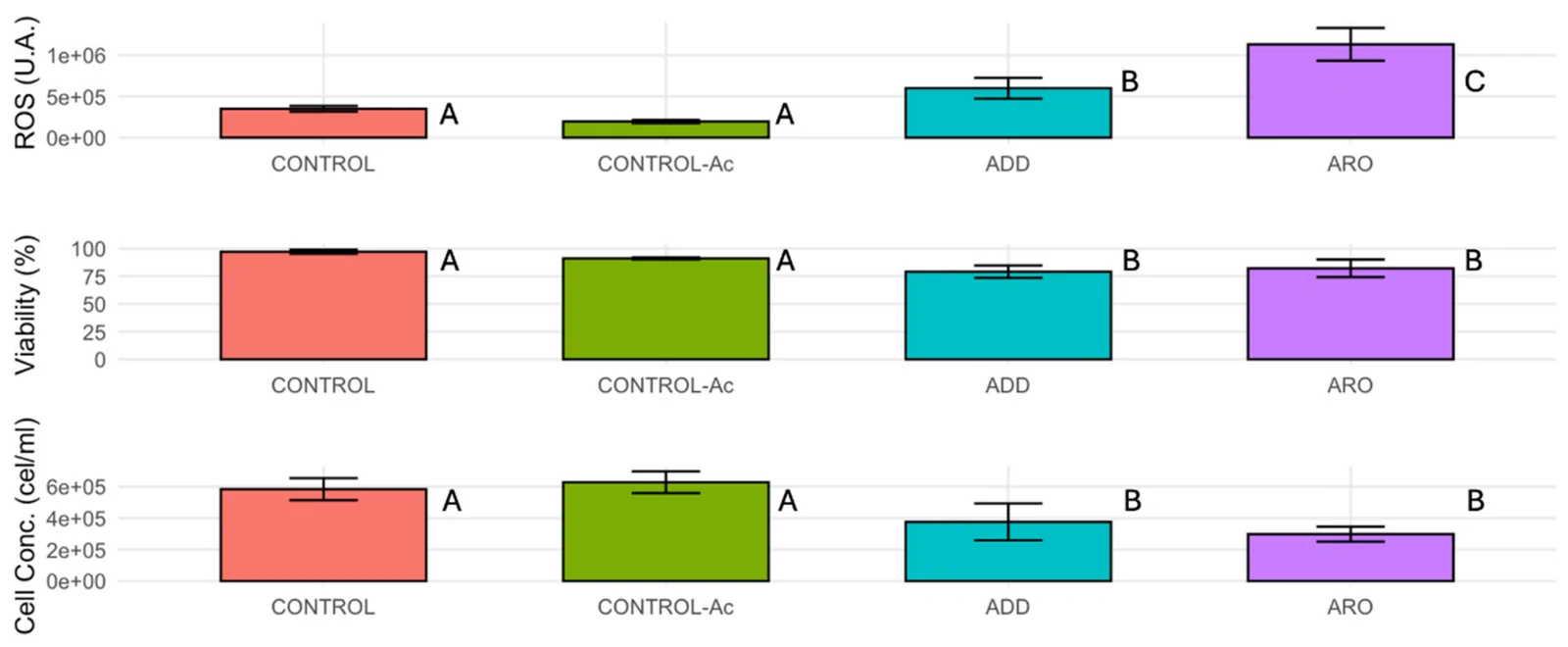
Flow cytometric analysis of intracellular ROS production, metabolic viability assessed by FDA staining, and cell concentration in HCT-116 cells treated with *D. dichotoma* (ADD) and *R. okamurae* (ARO) extracts. Data are expressed as mean ± standard deviation (SD) of three independent replicates (*n* = 3). Different uppercase letters (A–C) adjacent to bars denote statistically significant differences between experimental groups within each panel (*p* < 0.05, one-way ANOVA followed by Tukey’s post-hoc test; groups sharing the same letter are not significantly different).

Exposure of HCT-116 cells to the acetone extracts of *D. dichotoma* (ADD) and *R. okamurae* (ARO) triggered distinct physiological alterations, characterised by marked increases in intracellular oxidative stress together with significant reductions in metabolic viability and absolute cell concentration (Figure 4). Intracellular reactive oxygen species (ROS) levels exhibited a marked increase upon treatment, rising significantly from 3.57 × 10^5^ arbitrary units (AU) in the untreated CONTROL to 5.95 × 10^5^ AU in the ADD group (*p* < 0.05). This oxidative response was most pronounced after ARO treatment, which increased intracellular ROS levels to 1.13 × 10^6^ AU, significantly exceeding values in all other experimental groups (*p* < 0.05; Figure 7). Representative flow cytometry histograms showing intracellular ROS production in control and treated cells are presented in Supplementary Material Figure S1.

Concurrently, metabolic viability, assessed by fluorescein diacetate (FDA) staining, remained at optimal baseline levels in the CONTROL (96.7%) and CONTROL-Ac (91.2%) cultures. However, viability declined significantly to 79.3% and 82.4% for the ADD and ARO treatments, respectively (*p* < 0.05; Figure 6). Supplementary Figure S2 shows representative FDA fluorescence histograms illustrating the effects of the treatments on HCT-116 cell metabolic viability. Because FDA fluorescence depends on intracellular esterase activity and retention of the fluorescent product within viable cells, the observed decrease is consistent with impaired cellular metabolic activity and/or membrane integrity.

The reduction in metabolic viability was accompanied by a marked decrease in absolute cell concentration. Absolute cell concentrations dropped significantly from a maximum baseline of 6.30 × 10^5^ cells mL^−1^ in the CONTROL-Ac group to 3.76 × 10^5^ cells mL^−1^ under ADD exposure and reached a minimum of 3.01 × 10^5^ cells mL^−1^ in the ARO-treated cultures (*p* < 0.05; Figure 7). Evaluating these figures relative to the initial seeding density (3.0 × 10^5^ cells mL^−1^) reveals that while CONTROL-Ac cultures doubled during the incubation period (6.30 × 10^5^ cells mL^−1^), populations exposed to ADD (3.76 × 10^5^ cells mL^−1^) and ARO (3.01 × 10^5^ cells mL^−1^) remained at or near their starting density. This demonstrates a clear cytostatic response characterised by robust antiproliferative growth arrest, rather than active cell death or net population regression. No significant differences were observed between the CONTROL and vehicle CONTROL-Ac across any evaluated metrics, confirming that the solvent carrier did not introduce detectable confounding artefacts.

Collectively, these findings demonstrate that both macroalgal extracts induced substantial oxidative stress accompanied by impaired metabolic activity and reduced cell accumulation. Although excessive ROS production is known to disrupt cellular redox homeostasis and damage proteins, membrane lipids, and nucleic acids, ROS accumulation alone does not identify the mechanism responsible for the observed cytotoxicity [47]. Therefore, Annexin V-FITC/PI staining was performed to determine whether these physiological alterations were associated with apoptotic cell death.

**Figure 8:**
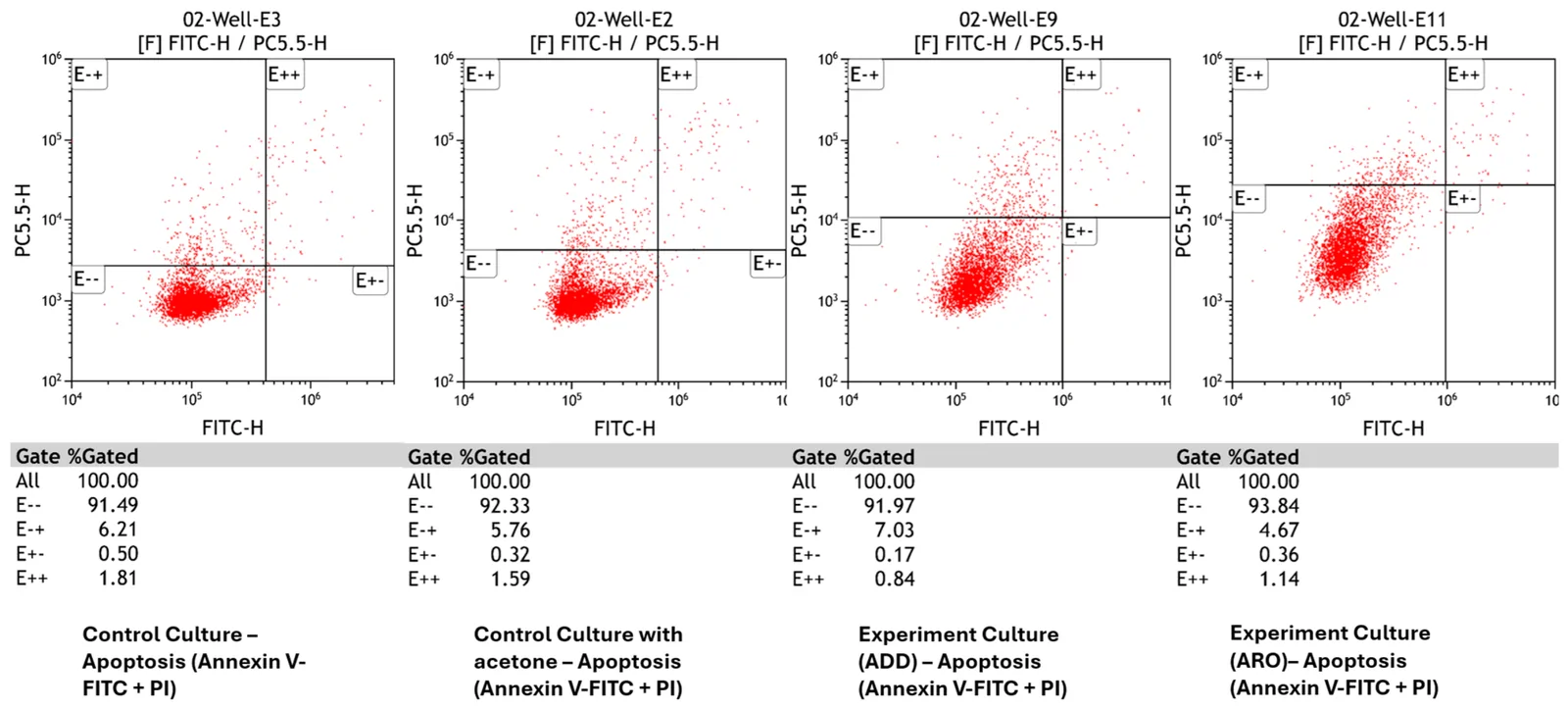
Representative Annexin V-FITC/PI dot plots showing viable, early apoptotic, late apoptotic, and necrotic populations of HCT-116 cells following treatment with *D. dichotoma* (ADD) and *R. okamurae* (ARO) extracts.

Despite the significant reductions in metabolic viability and cell concentration, Annexin V-FITC/PI analysis did not reveal a significant increase in apoptotic cell populations following treatment with either extract. Multiparametric gating of representative cultures (Figure 8) demonstrated that baseline viable populations forshowed that the baseline populations for CONTROL (91.49% viable, E−−) and CONTROL-Ac (92.33% viable, E−−) remained stable and healthy, indicating minimal spontaneous apoptosis throughout the experiment. Furthermore, exposure to the ADD and ARO extracts did not trigger a substantial shift toward the Annexin V-negative/PI-positive quadrant (E−+), indicative of primary necrosis, nor to the Annexin V-positive/PI-positive quadrant (E++), representing late apoptosis or secondary necrosis. The absence of prominent PI-positive populations indicates that an acute loss of plasma membrane integrity is not the primary driver of the observed cytotoxicity (Figure 8).

To confirm the absence of apoptotic induction across independent experiments, cell population percentages from three biological replicates (*n* = 3) were quantified and statistically analyzed (Supplementary Table S1). Viable cell populations remained consistently high across all groups, averaging 92.3 ± 1.1% in CONTROL-Ac, 92.0 ± 1.4% in ADD, and 93.8 ± 0.9% in ARO treatments (*p* > 0.05). Early apoptotic (E+−) populations remained negligible across all conditions (<0.5%), showing no statistically significant difference between vehicle control (0.32 ± 0.08%), ADD (0.17 ± 0.05%), and ARO (0.36 ± 0.09%) treatments (*p* > 0.05). Similarly, late apoptotic (E++) fractions remained below 1.8% across all treatments (1.59 ± 0.19% for CONTROL-Ac vs. 0.84 ± 0.14% for ADD and 1.14 ± 0.21% for ARO; *p* > 0.05). These quantitative evaluations statistically confirm that neither extract induces programmed cell death via apoptotic pathways Supplementary Figure S3 and Supplementary Table S9 (Supplementary Material) shows a representative apoptosis-positive control validating the Annexin V-FITC/PI staining procedure.

Taken together, these flow cytometry results complement the MTT assays by demonstrating that the marked reduction in cell viability produced by the diterpene-rich extracts is accompanied by increased intracellular ROS, impaired metabolic activity, and decreased cell accumulation, but not by a detectable increase in Annexin V-positive apoptotic populations or PI-positive necrotic populations. These findings suggest that classical apoptosis and acute necrosis are unlikely to be the predominant mechanism underlying the observed antiproliferative effects under the experimental conditions evaluated. Instead, the extracts may induce profound metabolic impairment, cytostatic responses, or alternative non-apoptotic cell death pathways. However, the present data do not allow discrimination among these possibilities, and additional studies evaluating cell-cycle progression, mitochondrial membrane potential, caspase activation, autophagy, and other regulated cell death pathways would be required to elucidate the underlying molecular mechanisms.

### 2.4. Limitations of This Study

Several limitations should be considered. First, the metabolomic analysis was performed on crude organic extracts obtained using acetone. Although this extraction strategy efficiently recovered low- and medium-polarity metabolites, particularly terpenoids, fatty acids, oxylipins, sterols, and other lipophilic compounds, highly polar metabolites such as polysaccharides, proteins, peptides, and many primary metabolites were likely underrepresented. Consequently, the metabolomic profiles presented here reflect the acetone-extractable fraction of the low- and medium-polarity metabolome rather than the complete chemical composition of each macroalgal species.

Second, while this study highlights distinct chemodiversity across the evaluated macroalgal phyla, it is important to acknowledge that the metabolomic profiles represent a baseline snapshot of each species based on a single collection event. Macroalgal secondary metabolism is highly dynamic and strongly influenced by environmental factors such as seasonal variation, temperature, geographical location, and reproductive stage. Because our sampling spanned different seasons and locations, the observed interspecific metabolic variations may be partially confounded by this environmental plasticity. Consequently, while these initial profiles successfully identify promising bioactive compound sources, future targeted investigations incorporating rigorous spatial and temporal biological replicates will be essential to fully disentangle constitutive species-specific signatures from environmentally induced metabolic responses.

Third, metabolite annotation was based primarily on untargeted LC-MS analysis, with most compounds assigned according to Metabolomics Standards Initiative (MSI) confidence Levels 2–4. Although these annotations provide a robust comparative overview of metabolite families, unequivocal structural confirmation would require purifying individual compounds and using complementary spectroscopic techniques such as nuclear magnetic resonance (NMR), along with comparison with authentic reference standards.

Fourth, the biological assays were performed using crude extracts rather than purified metabolites. While the chemical complexity of these crude extracts precludes attributing the observed cytotoxicity to a single compound or a definitive mechanism, the distinct cellular responses documented here provide a critical framework for future isolation-driven studies. Specifically, the preliminary phenotypic fingerprint induced by the most active extracts—characterised by substantial reactive oxygen species (ROS) generation and profound metabolic impairment without triggering classical apoptosis or acute necrosis—will serve as a targeted screening parameter for subsequent bioassay-guided fractionation.

Rather than relying solely on standard viability assays, future fractionation steps will use these specific flow cytometric markers to trace the bioactivity. Fractions that successfully replicate this unique cytostatic and ROS-inducing profile will be prioritised for further chromatographic separation and structural elucidation. This targeted translational pathway will allow us to determine whether the observed bioactivity stems from isolated specific metabolites, such as the prominent diterpenoids identified in *D. dichotoma*, or relies on the synergistic interactions among multiple compound classes within the extract. Fifth, biological evaluations were also restricted to in vitro screening using two human colorectal cancer cell lines (HT-29 and HCT-116) and one non-tumoral colon epithelial cell line (CCD 841 CoN). Although these models provide a useful initial assessment of cytotoxicity and selectivity, they cannot fully reproduce the complexity of the tumour microenvironment, including immune responses, stromal interactions, pharmacokinetics, and systemic metabolism. Therefore, readers should interpret the translational relevance of these findings with caution until confirmed in more physiologically relevant models.

Finally, the flow cytometry analyses demonstrated increased intracellular ROS production together with reduced metabolic viability and cell concentration but did not identify the precise molecular mechanism responsible for the antiproliferative effects. The absence of detectable apoptosis suggests that alternative mechanisms, including cell-cycle arrest, mitochondrial dysfunction, autophagy, ferroptosis, necroptosis, or other forms of regulated cell death, may be involved. Future studies combining transcriptomic, proteomic, metabolomic, and targeted biochemical analyses, together with in vivo validation, will be essential to elucidate the molecular targets of these bioactive metabolites and to assess their therapeutic potential.

Despite these limitations, integrating untargeted metabolomics with comparative biological screening across taxonomically distinct macroalgal species provides a robust framework for identifying metabolite classes associated with antiproliferative activity and prioritising promising species for future bioassay-guided isolation and mechanistic studies.

## 3. Materials and Methods

### 3.1. Reagents and Materials

Macroalgal extraction, extract reconstitution, and vehicle controls were performed using acetone (Sigma-Aldrich, St. Louis, MO, USA; Cat. 179124). Mobile phases for LC-MS analysis consisted of water containing 0.1% formic acid (Sigma-Aldrich, Cat. 1.00264) and LC-MS-grade acetonitrile (Sigma-Aldrich, Cat. 34851). For cell culture, RPMI-1640 medium (Sigma-Aldrich, Cat. R1145) was supplemented with fetal bovine serum (FBS) (Sigma-Aldrich, Cat. F7524), L-glutamine (Sigma-Aldrich, Cat. G8540), sodium pyruvate (Sigma-Aldrich, Cat. P5280), amphotericin B (Sigma-Aldrich, Cat. A2942), and penicillin–streptomycin (Sigma-Aldrich, Cat. P4333). TrypZean^®^ (Sigma-Aldrich, Cat. T3449) and calcium- and magnesium-free Dulbecco’s phosphate-buffered saline (DPBS) (Sigma-Aldrich, Cat. D8537) were used for cell detachment and washing, respectively. Cytotoxicity and metabolic assays utilized MTT (Sigma-Aldrich, Cat. 475989), DMSO (Sigma-Aldrich, Cat. D8418) for formazan dissolution, doxorubicin (Sigma-Aldrich, Cat. D5220) as a positive control, fluorescein diacetate (FDA) (Sigma-Aldrich, Cat. F7378) for metabolic viability, and 2′,7′-dichlorodihydrofluorescein diacetate (DCDHFDA) (Sigma-Aldrich, Cat. 287810) for intracellular ROS measurement. Apoptosis and necrosis were evaluated using the Annexin V-FITC/PI Apoptosis Detection Kit (Cat. TBK0508–0509; Tiaris Biosciences, Córdoba, Spain).

### 3.2. Samples

The macroalgal samples included in this study are listed in Table 2. Upon arrival at the laboratory, the specimens were washed, taxonomically identified, labelled, gently cleaned to remove adhering sand and epiphytes (if applicable), weighed, and froze at −18 °C until processing. Immediately before extraction, the samples were ground to a fine powder using a mortar and pestle to ensure sample homogeneity.

### 3.3. Preliminary Solvent Screening and Preparation of Bioassay Extracts

To identify the optimal solvent for recovering bioactive compounds, we conducted a preliminary solvent screening using *R. okamurae*. We subjected the raw macroalgal biomass to solid-liquid extraction using a panel of solvents—acetone, diethyl ether, methanol:water (80:20, *v*/*v*), and ethyl acetate. We prepared crude extracts by sequential solvent extraction. Briefly, we extracted 0.5 g of fresh algal material with 20 mL of solvent in an ultrasonic bath (50 °C, 5 min), then filtered the mixture. We evaporated the resulting extracts to dryness to calculate extraction yields, resuspended them in acetone, and evaluated their antiproliferative efficacy against the HT-29 colorectal cancer cell line. The final acetone concentration in the cell culture medium was kept well below the cytotoxic threshold for the cell line (<0.1% *v*/*v*).

Based on the preliminary bioassay results, the acetone-extracted extract exhibited the highest cytotoxic activity. Consequently, acetone was selected as the optimal extraction solvent. All macroalgal extracts evaluated in the subsequent biological assays (MTT and flow cytometry) were prepared by direct extraction of the biomass with acetone (hereafter referred to as “acetone extracts”). The extracts were dried and subsequently appropriately diluted for in vitro dosing.

### 3.4. Extraction and Characterisation of Metabolome by LC-MS

Crude extracts were prepared by solvent extraction. Briefly, 0.5 g of fresh algal material was extracted with 20 mL of acetone in an ultrasonic bath (50 °C, 5 min), followed by filtration and a second extraction. Subsequently, the extracts were centrifuged (10,000× *g*, 5 min), the supernatant was collected, and then the extracts were evaporated to dryness under a gentle stream of nitrogen at 50 °C. The dried extracts were stored at −20 °C until chemical and biological analyses.

This extraction used the same experimental conditions—including incubation time, temperature, and extract concentrations—as the primary cell viability assays described in the previous section.

Separations were conducted on a Vanquish Flex Quaternary LC system using a Hypersil Gold reverse-phase C18 column (100 mm × 2.1 mm, 1.9 μm; 0.3 mL min^−1^) with a mobile phase of acidified water (0.1% formic acid) and acetonitrile. Quality control (QC) samples prepared by pooling aliquots from all extracts and blank injections were included to monitor instrumental stability, assess mass accuracy, evaluate analytical reproducibility, and normalise the data.

The LC unit was coupled to a Thermo Fisher Scientific Orbitrap mass spectrometer operating in positive and negative ionisation modes (*m*/*z* 90–1000). Chromatographic data were processed using XcaliburTM 3.0 and Trace Finder 4.0. Feature filtering, alignment, and missing value imputations were applied before identification with Compound DiscovererTM 2.1. Identifications were categorised using Metabolomics Standards Initiative (MSI) levels, where Level 2 indicates matched literature spectra, Level 3 indicates tentative candidates based on MS/MS fragmentation, and Level 4 signifies an unequivocal molecular formula lacking reference spectra. The complete list of annotated metabolites and their relative abundances for each species is provided in the Supplementary Material.

### 3.5. Cell Viability Assay (MTT)

The antiproliferative activity of extracts from *R. okamurae*, *D. dichotoma*, *P. elongata*, *A. armata*, *C. bursa*, *E. selaginoides*, *Polysiphonia* spp., and *C. humilis* was evaluated against the human colorectal cancer cell lines HT-29 and HCT-116 and the normal colon epithelial cell line CCD 841 CoN. We obtained the cell lines from the Technical Instrumentation Service of the University of Granada (Granada, Spain) and routinely tested them to confirm the absence of *Mycoplasma* contamination.

Cells were maintained in RPMI-1640 medium supplemented with 5% fetal bovine serum, 2 mM L-glutamine, 1 mM sodium pyruvate, 0.125 mg mL^−1^ amphotericin B, and 100 U mL^−1^ penicillin–streptomycin at 37 °C in a humidified atmosphere containing 5% CO_2_. Cell culture and the MTT assay were performed as previously described [48].

For cytotoxicity assays, cells were seeded into 96-well plates at a density of 1 × 10^4^ viable cells per well and allowed to attach for 24 h before treatment. Algal extracts were dissolved in acetone and diluted in culture medium to final concentrations ranging from 0 to 300 μg mL^−1^. The amount of acetone used to redissolve the tested extracts was 1 mL. After 48 and 72 h of exposure to extracts, cell viability was determined by adding MTT solution (5 mg mL^−1^). Following incubation, the resulting formazan crystals were dissolved in 100 μL DMSO, and absorbance was measured at 570 nm using a microplate reader (Thermo Electron Corporation, Sant Cugat del Vallès, Spain).

Cell viability was expressed as a percentage relative to untreated controls according to the following equation:

Cell viability (%) = (Absorbance of treated cells/Absorbance of untreated cells) × 100.

The concentration required to inhibit cell growth by 50% (GI_50_) was calculated by nonlinear regression using sigmoidal dose-response curves. Doxorubicin (Sigma-Aldrich, Madrid, Spain) was used as the positive control, while acetone served as vehicle control. Acetone extracts and controls were evaluated in three independent biological experiments, each performed in triplicate, and results are presented as the mean ± standard error of the mean (SEM) [49].

### 3.6. Flow Cytometry Analysis

Human colorectal carcinoma HCT-116 cells were detached using TrypZean^®^ solution, washes with calcium- and magnesium-free DPBS, collected by centrifugation, and resuspended in fresh RPMI-1640 medium. Cell viability and concentration were determined by the trypan blue exclusion method using a Neubauer hemocytometer. Cells were seeded at a density of 3 × 10^5^ cells per T-12.5 flask in 3 mL of complete RPMI-1640 medium and incubated for 24 h at 37 °C in a humidified atmosphere containing 5% CO_2_ to allow cell attachment. Subsequently, the culture medium was replaced with fresh medium containing the algae extracts at their respective GI_50_ values determined after 72 h of treatment: *D. dichotoma* (4.5 µg mL^−1^) and *R. okamurae* (67 µg mL^−1^). Untreated cells and cells exposed to 0.1% acetone served as negative and vehicle controls, respectively. Following 72 h of treatment, both adherent and floating cells were collected to ensure recovery of apoptotic and necrotic populations. Cells were harvested by combining the culture medium, DPBS wash, and trypsinised fraction, centrifuged at 1600 rpm for 5 min, resuspended in fresh RPMI-1640 medium, and maintained on ice in the dark until flow cytometric analysis.

To evaluate the cells’ physiological status, flow cytometry analyses were performed using a CytoFLEX LX flow cytometer (Beckman Coulter, Brea, CA, USA), with all samples strictly maintained on ice before data acquisition. Absolute cell concentrations were directly determined using the cytometer’s volumetric fluidics system. Cell viability was evaluated via metabolic staining with fluorescein diacetate (FDA; Sigma-Aldrich, St. Louis, MO, USA) following incubation at a final concentration of 5 μM for 10 min in the dark. Intracellular reactive oxygen species (ROS) levels were quantified using 2′,7′-dichlorodihydrofluorescein diacetate (DCDHFDA; Sigma-Aldrich), where cells were incubated with 35 μM for 30 min in the dark to allow for intracellular oxidation into the fluorescent derivative. Cell apoptosis and necrosis were characterised using an Annexin V-FITC and Propidium Iodide (PI) commercial kit (TIARIS Biosciences, Córdoba, Spain), prepared and executed in strict accordance with the manufacturer’s instructions. For all assays, we collected a minimum of 10,000 events per sample and performed gating and data processing using Kaluza Analysis software v2.1 (Beckman Coulter). Cell debris and doublets were excluded prior to fluorescence analyses using forward- and side-scatter gating. Representative flow cytometry outputs used to support the analyses are provided in the Supplementary Material (Figures S1–S3 and Table S9).

### 3.7. Generative AI Usage

During the preparation of this manuscript, the authors used ChatGPT (OpenAI, GPT-5.5) exclusively as a graphical design assistant to generate the Graphical Abstract and Figure 1. All scientific concepts, figure organisation, annotations, and interpretations originated from the authors. The AI-generated output was critically reviewed, manually edited, corrected where necessary, and verified against the cited literature before inclusion. The authors assume full responsibility for the originality, accuracy, and final content of this figure.

### 3.8. Statistical Analysis

All data were analysed in R software (v4.3.2; R Foundation for Statistical Computing, Vienna, Austria). Cell viability assays by MTT and Flow Cytometry were performed in triplicate. Means and standard deviations (SD) were calculated for each experimental group and represented as error bars in figures. Differences between the controls (CONTROL and CONTROL-Ac) and the treatments (ADD and ARO) were tested using a one-way ANOVA followed by Tukey’s post-hoc test for multiple comparisons using Statgraphics^®^ Centurion XVI (v16.1.18; StatPoint Technologies, Warrenton, VA, USA). Results with a *p*-value of *p* < 0.05 were considered statistically significant. The heatmap was generated using Python (v3.11.5; Python Software Foundation, Wilmington, DE, USA) with the Seaborn (v0.13.0; sns.heatmap) and Matplotlib (v3.8.0) visualisation libraries, along with Pandas (v2.1.0) for data structuring.

## 4. Conclusions

By integrating comparative LC-MS metabolomics with in vitro cytotoxic screening across eight marine macroalgal species, this study demonstrates the usefulness of combining untargeted metabolomics with biological screening to identify metabolite classes associated with antiproliferative activity against human colorectal cancer cells. Rather than merely documenting chemical diversity, the comparative metabolomic approach identified diterpene-rich metabolite profiles in the brown algae *D. dichotoma* and *R. okamurae* that were consistently associated with the highest cytotoxic activities. Tentatively annotated diterpenoids, including dictyodial- and dilkamural-related compounds, are proposed as potential contributors to the observed antiproliferative effects. Conversely, species whose metabolomes were dominated by lipids, phytol-derived compounds, or structural metabolites, such as *C. bursa* and *P. elongata*, exhibited comparatively low cytotoxic activity, supporting an association between metabolite composition and biological response.

The comparative evaluation of HT-29 and HCT-116 colorectal cancer cell lines further demonstrated that tumour genetic background substantially influences sensitivity to macroalgal extracts. While *D. dichotoma* maintained high antiproliferative activity against both cell lines (GI_50_ ≈ 4.5–5 μg mL^−1^), *R. okamurae* exhibited markedly lower activity against HCT-116 cells (GI_50_ ≈ 67–69 μg mL^−1^) than against HT-29 cells. These findings indicate that diterpene abundance alone does not fully explain biological activity and highlight the importance of evaluating candidate marine extracts across multiple tumour models with distinct molecular characteristics.

Mechanistically, flow cytometry showed that exposure to the two most active extracts resulted in increased intracellular reactive oxygen species (ROS), reduced metabolic viability, and decreased cell concentration. However, no significant increase in Annexin V-positive cell populations was detected under the experimental conditions evaluated. These findings suggest that the antiproliferative effects are unlikely to be predominantly mediated through classical apoptotic pathways and may instead involve oxidative stress-associated metabolic impairment or alternative mechanisms of cell death, although further mechanistic studies are required to confirm these possibilities.

The present work also demonstrates the value of integrating comparative metabolomics with functional bioassays to prioritise macroalgal species for subsequent bioassay-guided fractionation and compound isolation. Future research should focus on purifying and structurally confirming bioactive diterpenoids, evaluating their individual and synergistic effects, elucidating their molecular targets using transcriptomic, proteomic, and biochemical approaches, and validating their efficacy and safety in appropriate in vivo models. Overall, this study identifies *D. dichotoma* and *R. okamurae* as promising sources of bioactive metabolites and provides a robust foundation for the future discovery and development of marine-derived anticancer agents.

## Figures and Tables

**Table 1:** Classification of the comparative tentative metabolite-family matrix across algal species *.

Species	Terpenoids/ Diterpenoids	Fatty Acids & Lipids	Oxylipins	Nitrogenous Compounds	Phenolics/Polyketides	Pigments/ Carotenoids	Sulfur Compounds	Halogenated Compounds	Volatiles/Small Organics
*Cystoseira* *humilis*	31.00 (2–3) ^b^	31.00 (3) ^b^	Incl. with FA	11.00 (3)	ND	ND	24.00 (3)	1.00 (3)	ND
*Rugulopteryx* *okamurae*	34.96 (2–3)	11.17 (2)	Incl. with FA	3.52 (2–3)	ND	0.91 (2–4)	ND	ND	1.48 (3)
*Dictyota* *dichotoma*	47.44 (2–4) ^c^	7.54 (2)	Incl. with FA	0.60 (2)	2.41 (2)	ND	ND	ND	ND
*Ericaria* *selaginoides*	27.00 (2)	2.00 (2)	Incl. with terpenoids	30.00 (3)	2.00 (3)	ND	5.00 (3)	ND	ND
*Codium bursa*	38.65 (2–3)	25.23 (2–3) ^a^	Incl. with FA	3.50 (2)	0.85 (3)	4.50 (3)	ND	ND	1.33 (2–3)
*Asparagopsis* *armata*	9.53 (2–3)	17.09 (2–3)	9.15 (2–3)	12.48 (2–4)	7.51 (2–3)	3.40 (2)	ND	ND	ND
*Polysiphonia* *elongata*	15.20 (3)	16.14 (2)	3.45 (2)	10.48 (3)	4.72 (3)	ND	ND	ND	ND
*Polysiphonia * spp.	11.00 (3–4)	51.00 (3–4)	Incl. with FA	23.00 (3–4)	Incl. with terpenoids	ND	5.00 (3–4)	2.00 (4)	ND

* Values represent relative abundance (%) of each metabolite family, with the corresponding Metabolomics Standards Initiative (MSI) confidence level shown in brackets. ND: Not Detected/below detection threshold; Incl.: Included within indicated class. ^a^ Includes phospholipids (3.84%) within the broader lipid category. ^b^ Original category reported as “Fatty Acids, Oxylipins, Polyols and Terpenoids”; value retained in both relevant columns for comparison. ^c^ Sum of diterpenoids (43.49%) and sesquiterpenoids/norisoprenoids (3.95%). Species names are color-coded by their respective phyla: brown text for Ochrophyta/Phaeophyceae or brown seaweeds, green text for Chlorophyta or green seaweeds, and red text for Rhodophyta or red seaweeds).

**Table 2:** Summary of Mediterranean seaweed species collection.

Phyla of Marine Macroalgae	Seaweed Species	Code	Date of Collection	Location	Geographical Coordinates
Ochrophyta/Phaeophyceae (Brown Seaweeds)	*Cystoseira humilis*	CHR	2 October 2022	Roquetas de Mar, Almería	36.712321, −2.635997
	*Rugulpteryx okamurae*	RB1	12 April 2023	Manilva, Málaga	36.318979, −5.244384
	*Dictyota dichotoma*	DA1	1 March 2023	Almerimar, Almería	36.705501, −2.811383
	*Ericaria selaginoides*	ESC	3 March 2022	Carchuna, Granada	36.695148, −3.440386
Chlorophyta (Green Seaweeds)	*Codium bursa*	CB1	21 January 2024	Toyo Beach, Almería	36.835711, −2.326845
Rhodophyta (Red Seaweeds)	*Asparagopsis armata*	AAR	28 November 2022	Toyo Beach, Almería	36.835711, −2.326845
	*Polysiphonia elongata*	PEE	22 November 2022	Estepona, Málaga	36.415978, −5.173438
	*Polysiphonia * spp.	PSC	20 January 2024	Toyo Beach, Almería	36.835711, −2.326845

Species names are color-coded by their respective phyla: brown text for Ochrophyta/Phaeophyceae or brown seaweeds, green text for Chlorophyta or green seaweeds, and red text for Rhodophyta or red seaweeds).

## Data Availability

All data generated or analysed during this study are included in this published article and its Supplementary Information files.

## Supplementary Materials

The following supporting information can be downloaded at: https://www.mdpi.com/article/10.3390/md24090329/s1.
