# Supplementary material for: Comparative Metabolomics Identifies Diterpene-Rich Marine Macroalgae with Potent Antiproliferative Activity Against Colorectal Cancer Cells
Source: Mar Drugs. 2026 Sep 21;24(9):329. doi: 10.3390/md24090329 (PMC13608122; doi:10.3390/md24090329)
Supplement: Supplementary file 1 [file marinedrugs-24-00329-s001.zip › marinedrugs-4517241-supplementary.pdf]

SUPPLEMENTARY MATERIAL INDEX

"Comparative Metabolomics Identifies Diterpene-Rich Marine Macroalgae with Potent Antiproliferative Activity Against Colorectal Cancer Cells"

Supplementary Tables on Metabolomic Profile

- **Table S1.** ***Cystoseira humilis*** 2
- **Table S2.** ***Rugulopteryx okamurae*** 10
- **Table S3.** ***Dictyota dichotoma*** 14
- **Table S4.** ***Ericaria selaginoides*** 19
- **Table S5.** ***Codium bursa*** 26
- **Table S6.** ***Asparagopsis armata*** 30
- **Table S7.** ***Polysiphonia elongata*** 35
- **Table S8.** ***Polysiphonia* ssp.** 40

Supplementary Material about Flow Cytometry. Figures and tables... 47

- **Supplementary Figure S1. ROS**.. 47
- **Supplementary Figure S2. Viability (Fluorescein diacetate)** 48
- **Supplementary Figure S3. Apoptosis-necrosis** 49
- **Table S9. Quantified Population Breakdown Across Replicates** 49

Comprehensive LC–HRMS Metabolite Annotation and Profiling of Eight Mediterranean Macroalgal Species

Supplementary tables S1–S8: Detailed chemical classifications, retention times, observed mass-to-charge ratios (m/z), adducts, formula assignments, and Metabolomics Standards Initiative (MSI) identification confidence levels

Table S1. *Cystoseira humilis* Metabolite Profile

Family	Tentative identification	Rt (min)	m/z	Molecular Formula	Ionization Mode	Rel. Area (%)	MSI
**Fatty Acids, Oxylipins, Polyols, and Terpenoids (C, H, O only)**	Arachidonic acid	29.28	303.23	C20 H32 O2	$[M-H]^-$	5.93	2
	Myristic acid	28.54	227.20	C14 H28 O2	$[M-H]^-$	3.90	2
	Linolenic acid isomer	26.49	277.22	C18 H30 O2	$[M-H]^-$	2.97	2
	Palmitoleic acid	28.94	253.22	C16 H30 O2	$[M-H]^-$	2.70	2
	EPA	25.7	301.22	C20 H30 O2	$[M-H]^-$	1.77	2
	Palmitoleic acid isomer	29.74	253.22	C16 H30 O2	$[M-H]^-$	1.49	3
	Hydroxy-octadecadienoic acid	20.60	295.23	C18 H32 O3	$[M-H]^-$	1.26	3
	Oxidized sugar / Polyol	1.65	185.07	C5 H12 O7	$[M+H]^+$	0.75	3
	Very long-chain PUFA (22:5)	28.05	327.23	C22 H32 O2	$[M-H]^-$	0.63	3
	Hexanoic acid	1.73	117.09	C6 H12 O2	$[M+H]^+$	0.63	2
	Oxo-octadecatrienoic acid	21.24	293.21	C18 H30 O3	$[M-H]^-$	0.55	2
	Stearidonic acid	23.86	275.2	C18 H28 O2	$[M-H]^-$	0.39	2
	Stearidonic acid isomer	21.26	277.22	C18 H28 O2	$[M+H]^+$	0.34	3
	Oxylipin isomer	19.73	293.21	C18 H30 O3	$[M-H]^-$	0.30	3
	Hydroxy-eicosatetraenoic acid	20.81	319.23	C20 H32 O3	$[M-H]^-$	0.30	2
	Highly oxygenated lipid / Glycoside	24.15	403.23	C20 H34 O8	$[M+H]^+$	0.30	3
	Alkyl diol / Hydroxylated lipid	25.35	319.28	C18 H38 O4	$[M+H]^+$	0.29	3
	Complex lipid / Glycolipid	27.86	621.49	C34 H68 O9	$[M+H]^+$	0.27	3
	Glycolipid fragment	23.53	465.31	C23 H46 O9	$[M-H]^-$	0.26	3
	PUFA isomer	28.31	303.23	C20 H32 O2	$[M-H]^-$	0.22	3
	EPA isomer	27.49	301.22	C20 H30 O2	$[M-H]^-$	0.21	3

Oxidized sterol derivative	20.75	425.27	C27 H36 O4	$[M+H]^+$	0.21	3
Terpenoid / Long-chain volatile	23.14	311.20	C21 H28 O2	$[M-H]^-$	0.19	3
Oxygenated lipid	17.25	293.18	C17 H26 O4	$[M-H]^-$	0.18	3
PUFA isomer	20.62	279.23	C18 H30 O2	$[M+H]^+$	0.18	3
Terpenoid derivative	28.61	295.21	C21 H28 O	$[M-H]^-$	0.16	3
PUFA isomer	28.71	277.22	C18 H30 O2	$[M-H]^-$	0.15	3
Dihydroxy-eicosatetraenoic acid	19.23	335.22	C20 H32 O4	$[M-H]^-$	0.15	3
Hydroxy-octadecenoic acid	21.87	297.24	C18 H34 O3	$[M-H]^-$	0.14	3
Polyhydroxylated alkyl chain	25.03	363.31	C20 H42 O5	$[M+H]^+$	0.14	3
Long-chain lipid / wax ester	28.43	417.36	C24 H48 O5	$[M+H]^+$	0.13	3
Pentadecanoic acid	26.33	239.20	C15 H28 O2	$[M-H]^-$	0.13	2
PUFA isomer	19.73	277.22	C18 H28 O2	$[M+H]^+$	0.13	3
Mannitol	1.43	181.07	C6 H14 O6	$[M-H]^-$	0.13	3
Oxylipin isomer	21.31	319.23	C20 H32 O3	$[M-H]^-$	0.12	3
Oxylipin	19.97	317.21	C20 H30 O3	$[M-H]^-$	0.11	3
Phthalic anhydride	17.21	149.02	C8 H4 O3	$[M+H]^+$	0.11	3
Octitol / Sugar derivative	1.68	243.11	C8 H18 O8	$[M+H]^+$	0.11	3
Oxylipin isomer	21.49	293.21	C18 H30 O3	$[M-H]^-$	0.11	3
Oxylipin isomer	22.77	295.23	C18 H32 O3	$[M-H]^-$	0.10	3
Terpenoid / Volatile	27.64	259.21	C18 H26 O	$[M+H]^+$	0.10	3
Dihydroxy fatty acid	18.42	311.22	C18 H32 O4	$[M-H]^-$	0.10	3
Dihydroxy fatty acid isomer	18.62	311.22	C18 H32 O4	$[M-H]^-$	0.10	3
Oxidized PUFA	17.95	309.21	C18 H30 O4	$[M-H]^-$	0.10	3
Terpenoid derivative	21.51	307.21	C22 H26 O	$[M+H]^+$	0.10	3
Diterpene derivative	20.05	301.22	C20 H28 O2	$[M+H]^+$	0.09	3
Oxylipin	19.03	291.2	C18 H28 O3	$[M-H]^-$	0.09	3
Myristic acid isomer	27.41	227.2	C14 H28 O2	$[M-H]^-$	0.08	3
Hydroxylated saturated lipid	25.84	275.26	C16 H34 O3	$[M+H]^+$	0.08	3
Glycolipid fragment	27.86	665.49	C35 H70 O11	$[M-H]^-$	0.08	3

	Sterol/Terpenoid	29.53	373.25	C27 H32 O	$[M+H]^{+}$	0.08	3
	Glycolipid	23.16	563.35	C28 H52 O11	$[M-H]^{-}$	0.08	3
	Oxylipin isomer	18.60	295.23	C18 H30 O3	$[M+H]^{+}$	0.08	3
	Small aromatic volatile	2.21	105.07	C8 H8	$[M+H]^{+}$	0.08	3
	Hexadecadienoic acid	24.85	251.2	C16 H28 O2	$[M-H]^{-}$	0.08	2
	Oxylipin isomer	21.12	319.23	C20 H32 O3	$[M-H]^{-}$	0.08	3
	Sesquiterpenoid derivative	21.26	179.14	C12 H18 O	$[M+H]^{+}$	0.08	3
	Short-chain volatile	15.93	181.12	C11 H16 O2	$[M+H]^{+}$	0.08	3
	Hydroxy-octadecenoic acid isomer	21.35	297.24	C18 H34 O3	$[M-H]^{-}$	0.08	3
	Tetradecadienoic acid / Dicarboxylic	23.80	225.19	C14 H26 O2	$[M-H]^{-}$	0.08	3
	Small polar acid	1.64	127.02	C2 H6 O6	$[M+H]^{+}$	0.07	3
	Oxidized long-chain lipid	23.16	357.3	C21 H40 O4	$[M+H]^{+}$	0.07	3
	Terpenoid derivative	22.03	335.26	C21 H34 O3	$[M+H]^{+}$	0.07	3
	Oxylipin / Nonadecatrienoic derivative	25.95	309.24	C19 H32 O3	$[M+H]^{+}$	0.07	3
	Alkyl diol isomer	19.92	319.28	C18 H38 O4	$[M+H]^{+}$	0.07	3
	Complex long-chain lipid	28.43	385.33	C23 H44 O4	$[M+H]^{+}$	0.07	3
	Hydroxy-octadecenoic acid isomer	21.59	297.24	C18 H34 O3	$[M-H]^{-}$	0.07	3
	Oxylipin isomer	18.42	295.23	C18 H30 O3	$[M+H]^{+}$	0.07	3
	Complex terpenoid	26.09	347.24	C25 H30 O	$[M+H]^{+}$	0.07	3
	Hydroxy-eicosatrienoic acid	21.58	321.24	C20 H34 O3	$[M-H]^{-}$	0.07	3
	Hydroxy-palmitoleic acid	26.01	269.21	C16 H30 O3	$[M-H]^{-}$	0.07	3
	Phenolic derivative	17.21	177.05	C10 H8 O3	$[M+H]^{+}$	0.07	3
**Sulfur-Containing Compounds and Sulfolipids**	Dodecyl sulfate derivative	18.12	265.15	C12 H26 O4 S	$[M-H]^{-}$	3.85	3
	Sulfated octadecatrienoic acid	20.12	325.19	C18 H30 O3 S	$[M-H]^{-}$	2.96	3
	Tetradecyl sulfate derivative	20.65	293.18	C14 H30 O4 S	$[M-H]^{-}$	2.75	3

Hydroxytetradecyl sulfate	19.12	309.18	C14 H30 O5 S	$[M-H]^-$	2.32	3
Taurine/Cysteamine derivative	1.23	214.09	C10 H15 N O2 S	$[M+H]^+$	1.77	3
Hydroxyhexadecyl sulfate	22.95	337.21	C16 H34 O5 S	$[M-H]^-$	1.60	3
Complex N/S marine metabolite	19.63	353.2	C17 H30 N4 O2 S	$[M-H]^-$	1.31	3
Cysteic acid derivative	0.33	167.01	C3 H6 N2 O4 S	$[M+H]^+$	1.06	3
S-containing alkaloid/peptide	19.90	375.16	C22 H24 N4 S	$[M-H]^-$	0.74	3
Sulfur-containing peptide	19.93	397.23	C19 H34 N4 O3 S	$[M-H]^-$	0.74	3
Complex marine N/S compound	20.14	441.25	C22 H34 N8 S	$[M-H]^-$	0.39	3
Sulfated hexadecadienoic acid	18.04	297.15	C16 H26 O3 S	$[M-H]^-$	0.37	3
Taurine derivative	0.32	139.02	C2 H6 N2 O3 S	$[M+H]^+$	0.32	3
Disulfide/dithio-terpenoid	23.55	455.28	C29 H44 S2	$[M-H]^-$	0.30	3
Sulfated heptadecadienoic acid	19.59	311.17	C17 H28 O3 S	$[M-H]^-$	0.30	3
Complex cyclic N/S compound	19.76	391.16	C22 H24 N4 O S	$[M-H]^-$	0.26	3
SQDG fragment / lyso-sulfolipid	19.3	555.29	C25 H48 O11 S	$[M-H]^-$	0.26	3
SQDG fragment	20.28	485.28	C22 H46 O9 S	$[M-H]^-$	0.21	3
Small sulfonated peptide/amine	1.65	201.04	C6 H8 N4 O2 S	$[M+H]^+$	0.21	3
Highly basic S-compound	0.33	186.02	C3 H3 N7 O S	$[M+H]^+$	0.2	3
Amino acid derivative	1.24	158.03	C6 H7 N O2 S	$[M+H]^+$	0.18	3
Complex oligopeptide	20.41	529.31	C21 H42 N10 O4 S	$[M-H]^-$	0.13	3
Small alkyl sulfate	1.65	143	C2 H6 O5 S	$[M+H]^+$	0.12	3
Polyamine sulfate derivative	20.14	460.29	C16 H41 N7 O6 S	$[M+H]^+$	0.12	3
Sulfated fatty acid	18.82	275.17	C14 H28 O3 S	$[M-H]^-$	0.23	3
Sulfated fatty acid isomer	19.10	275.17	C14 H28 O3 S	$[M-H]^-$	0.12	3
Small sulfated amine	0.33	185.02	C3 H8 N2 O5 S	$[M+H]^+$	0.12	3
Sulfated fatty acid isomer	18.41	297.15	C16 H26 O3 S	$[M-H]^-$	0.10	3
Polyamine sulfate	19.92	416.27	C14 H37 N7 O5 S	$[M+H]^+$	0.10	3
Nitrogenous sulfur compound	0.81	171.99	C3 H N5 O2 S	$[M+H]^+$	0.10	3
Complex cyclic isomer	21.37	391.16	C22 H24 N4 O S	$[M-H]^-$	0.09	3
S-containing lipopeptide	19.07	351.22	C18 H32 N4 O S	$[M-H]^-$	0.09	3

	S-containing alkaloid/lipid	26.70	364.23	C21 H33 N O2 S	$[M+H]^+$	0.08	3
	Thiophene / small aromatic	1.26	141.00	C6 H4 O2 S	$[M+H]^+$	0.08	3
	Complex N/S compound	20.30	504.32	C18 H45 N7 O7 S	$[M+H]^+$	0.08	3
	Peptidic sulfolipid	20.15	465.25	C18 H36 N6 O6 S	$[M+H]^+$	0.07	3
	Sulfated glycoside	18.13	333.14	C15 H26 O6 S	$[M-H]^-$	0.07	3
	Sphingolipid / S-lipid	27.79	438.38	C27 H51 N O S	$[M+H]^+$	0.07	3
**Halogenated Compounds (Chlorine-containing)**	High mass chlorinated lipopeptide	28.58	494.31	C29 H42 Cl N5	$[M-H]^-$	0.16	3
	Dichloro-docosane derivative	26.03	377.27	C22 H44 Cl2	$[M-H]^-$	0.13	3
	Chlorinated/sulfated polar metabolite	18.14	323.11	C10 H21 Cl N6 O2 S	$[M-H]^-$	0.12	3
	Small chlorinated S-compound	0.30	169.05	C6 H13 Cl O S	$[M+H]^+$	0.11	3
	Chlorohydroxy fatty acid	20.62	331.21	C18 H33 Cl O3	$[M-H]^-$	0.10	3
	Complex aromatic Cl/S compound	24.79	509.07	C29 H19 Cl N2 O3 S	$[M-H]^-$	0.09	3
	Chloro-diterpenoid / Halolipid	29.27	339.21	C20 H33 Cl O2	$[M-H]^-$	0.08	3
	Large chlorinated lipopeptide	27.93	655.46	C35 H65 Cl N4 O5	$[M-H]^-$	0.08	3
	Chlorosulfated diterpenoid	20.17	383.14	C20 H29 Cl O3 S	$[M-H]^-$	0.08	3
**Nitrogen-Containing Compounds (Amino Acids, Peptides, Polyamines)**	High mass chlorinated lipopeptide	28.58	494.31	C29 H42 Cl N5	$[M-H]^-$	0.16	3
	Dichloro-docosane derivative	26.03	377.27	C22 H44 Cl2	$[M-H]^-$	0.13	3
	Chlorinated/sulfated polar metabolite	18.14	323.11	C10 H21 Cl N6 O2 S	$[M-H]^-$	0.12	3
	Small chlorinated S-compound	0.30	169.05	C6 H13 Cl O S	$[M+H]^+$	0.11	3
	Chlorohydroxy fatty acid (Oxylipin)	20.62	331.21	C18 H33 Cl O3	$[M-H]^-$	0.10	3
	Complex aromatic Cl/S compound	24.79	509.07	C29 H19 Cl N2 O3 S	$[M-H]^-$	0.09	3
	Chloro-diterpenoid / Halolipid	29.27	339.21	C20 H33 Cl O2	$[M-H]^-$	0.08	3
	Large chlorinated lipopeptide	27.93	655.46	C35 H65 Cl N4 O5	$[M-H]^-$	0.08	3
	Chlorosulfated diterpenoid	20.17	383.14	C20 H29 Cl O3 S	$[M-H]^-$	0.08	3
	Polyamine / Long-chain base	25.03	380.34	C21 H41 N5 O	$[M+H]^+$	0.33	3

Alkylated polyamine	25.35	336.31	C19 H37 N5	$[M+H]^+$	0.32	3
Cyclic N-compound	21.26	317.21	C16 H24 N6 O	$[M+H]^+$	0.31	3
Lipopeptide	24.76	424.36	C23 H45 N5 O2	$[M+H]^+$	0.28	3
N-alkylated amino acid	16.6	228.2	C13 H25 N O2	$[M+H]^+$	0.28	3
Complex basic lipid	20.73	371.33	C21 H42 N2 O3	$[M+H]^+$	0.28	3
Complex basic lipid	17.61	343.3	C19 H38 N2 O3	$[M+H]^+$	0.24	3
Complex alkaloid/peptide	20.31	473.26	C26 H36 N2 O6	$[M+H]^+$	0.24	3
Small peptide / Amino acid	0.30	164.07	C4 H9 N3 O4	$[M+H]^+$	0.23	3
Small alkylamine (e,g,, Choline deriv,)	1.46	104.11	C5 H13 N O	$[M+H]^+$	0.23	3
Large lipopeptide	22.37	572.38	C31 H49 N5 O5	$[M+H]^+$	0.22	3
Oligopeptide	24.16	425.21	C18 H28 N6 O6	$[M+H]^+$	0.21	3
Complex N-lipid	24.52	468.39	C25 H49 N5 O3	$[M+H]^+$	0.21	3
Dipeptide / N-glycoside	18.63	335.22	C15 H30 N2 O6	$[M+H]^+$	0.21	3
Tripeptide derivative	21.34	394.2	C19 H27 N3 O6	$[M+H]^+$	0.20	3
Glycosylated amine	17.76	418.28	C21 H39 N O7	$[M+H]^+$	0.20	3
Large polycyclic N-compound	28.57	492.31	C27 H39 N7 O2	$[M-H]^-$	0.20	3
Highly oxidized small amine	1.64	187.06	C3 H10 N2 O7	$[M+H]^+$	0.19	3
Oligopeptide	17.73	423.23	C19 H30 N6 O5	$[M+H]^+$	0.18	3
Fatty acid amide (e,g,, Erucamide)	21.65	338.34	C22 H43 N O	$[M+H]^+$	0.17	3
Complex basic lipid	26.33	399.36	C23 H46 N2 O3	$[M+H]^+$	0.17	3
Complex cyclic N-compound	19.67	399.25	C21 H30 N6 O2	$[M+H]^+$	0.17	3
Polar amine isomer	2.28	123.04	C2 H6 N2 O4	$[M+H]^+$	0.17	3
Lipopeptide	21.99	498.39	C27 H51 N3 O5	$[M+H]^+$	0.17	3
Oxidized amino acid	2.03	168.05	C4 H9 N O6	$[M+H]^+$	0.16	3
Lipopeptide	24.3	512.42	C27 H53 N5 O4	$[M+H]^+$	0.16	3
Oxidized amino acid isomer	1.62	168.05	C4 H9 N O6	$[M+H]^+$	0.16	3
Small cyclic amine / amide	2.00	100.08	C5 H9 N O	$[M+H]^+$	0.14	3
Oleamide derivative	24.1	282.28	C18 H35 N O	$[M+H]^+$	0.14	3

Small polar amine	1.62	140.06	C3 H9 N O5	$[M+H]^+$	0.14	3
Complex glycosphingolipid	27.85	643.47	C31 H66 N2 O11	$[M+H]^+$	0.13	3
Complex glycosphingolipid	23.16	536.38	C27 H53 N O9	$[M+H]^+$	0.13	3
N-lipid derivative	26.31	378.3	C23 H39 N O3	$[M+H]^+$	0.12	3
Small nitrogenous metabolite	0.3	170.04	C2 H7 N3 O6	$[M+H]^+$	0.12	3
Polyamine lipid	25.36	341.27	C16 H32 N6 O2	$[M+H]^+$	0.12	3
Fatty acid amide	26.99	336.33	C22 H41 N O	$[M+H]^+$	0.12	3
Small polar amine isomer	2.02	140.06	C3 H9 N O5	$[M+H]^+$	0.12	3
Aromatic nitrogenous compound	0.34	208.04	C13 H5 N O2	$[M+H]^+$	0.11	3
Polyamine / Tripeptide	16.59	250.18	C10 H23 N3 O4	$[M+H]^+$	0.11	3
Simple alkylamine	0.31	130.16	C8 H19 N	$[M+H]^+$	0.10	3
Large lipopeptide	24.10	556.44	C29 H57 N5 O5	$[M+H]^+$	0.10	3
Small polar N-compound	1.66	186.06	C4 H11 N O7	$[M+H]^+$	0.10	3
Small amine	0.19	102.07	C3 H7 N3 O	$[M+H]^+$	0.10	3
Purine/Pyrimidine base	1.54	137.05	C5 H4 N4 O	$[M+H]^+$	0.10	3
Complex N-lipid isomer	21.34	451.32	C25 H42 N2 O5	$[M+H]^+$	0.10	3
Glycosylated N-lipid	22.46	577.33	C27 H48 N2 O11	$[M+H]^+$	0.09	3
Polyaromatic N-compound	19.93	421.22	C20 H24 N10 O	$[M+H]^+$	0.09	3
Hydroxylated fatty amide	21.66	356.35	C22 H45 N O2	$[M+H]^+$	0.09	3
Oxidized amino acid derivative	2.09	226.09	C7 H15 N O7	$[M+H]^+$	0.09	3
Alkylated pyridine/aniline	2.15	122.1	C8 H11 N	$[M+H]^+$	0.09	3
Lipopeptide	28.57	369.24	C19 H34 N2 O5	$[M-H]^-$	0.09	3
Hydroxylated fatty amide	29.13	352.32	C22 H41 N O2	$[M+H]^+$	0.09	3
Complex polyamine/alkaloid	29.28	618.43	C39 H51 N7	$[M+H]^+$	0.08	3
Complex polyamine/alkaloid isomer	28.04	618.43	C39 H51 N7	$[M+H]^+$	0.08	3
Cyclic N-compound	17.95	333.2	C16 H24 N6 O2	$[M+H]^+$	0.08	3
Alkaloid derivative	18.81	374.23	C22 H31 N O4	$[M+H]^+$	0.08	3
Benzimidazole / Indazole	1.65	117.04	C7 H4 N2	$[M+H]^+$	0.08	3

Very large lipidic amine	29.28	607.47	C41 H60 N4	$[M-H]^-$	0.08	3
Oligopeptide	23.16	541.33	C26 H40 N10 O3	$[M+H]^+$	0.08	3
Lipopeptide	22.69	510.36	C26 H47 N5 O5	$[M+H]^+$	0.08	3
Complex N-lipid	21.14	381.27	C23 H34 N4 O	$[M-H]^-$	0.08	3
Basic lipid isomer	17.41	343.29	C19 H38 N2 O3	$[M+H]^+$	0.08	3
Highly basic small molecule	1.68	175.04	C6 H2 N6 O	$[M+H]^+$	0.07	3
Lipopeptide	20.94	439.32	C24 H42 N2 O5	$[M+H]^+$	0.07	3
Complex alkaloid/peptide	23.15	438.28	C24 H39 N O6	$[M+H]^+$	0.07	3
Polyamine	25.84	297.24	C14 H28 N6 O	$[M+H]^+$	0.07	3
Oligopeptide / Polyamine	29.57	377.27	C13 H34 N10 O3	$[M-H]^-$	0.07	3
Amino acid derivative	10.44	172.13	C9 H17 N O2	$[M+H]^+$	0.07	3
Small dipeptide	1.73	119.08	C4 H10 N2 O2	$[M+H]^+$	0.07	3
N-alkylated compound	17.22	186.19	C11 H23 N O	$[M+H]^+$	0.07	3
Large peptide/glycoside	21.19	563.33	C30 H46 N2 O8	$[M+H]^+$	0.07	3
Cyclic polyamine / Peptide	22.10	375.25	C19 H30 N6 O2	$[M+H]^+$	0.07	3

Table S2. ***Rugulopteryx okamurae*** **Metabolite Profile**

Compound Family	Tentative identification	RT (min)	m/z	Molecular Formula	Ionization Mode	Rel. Area (%)	MSI level
**Core Diterpenoids (Spatane / Secospatane Skeleton)** This family constitutes the principal secondary metabolome of *R. okamurae*. These are non-esterified C20 structures varying strictly by their oxidation states (hydroxyls, ketones, or double-bond positions)	Di-oxygenated Diterpene (Isomer 1-11)	21.11	299.20	C20H26O2	$[M+H]^+$	5.43	3
		20.50	299.20	C20H26O2	$[M+H]^+$	3.23	3
		21.77	299.20	C20H26O2	$[M+H]^+$	1.13	3
		27.03	299.20	C20H26O2	$[M+H]^+$	0.85	3
		28.90	299.20	C20H26O2	$[M+H]^+$	0.82	3
		23.04	299.20	C20H26O2	$[M+H]^+$	0.50	3
		18.75	299.20	C20H26O2	$[M+H]^+$	0.48	3
		28.31	299.20	C20H26O2	$[M+H]^+$	0.46	3
		18.28	299.20	C20H26O2	$[M+H]^+$	0.45	3
		22.16	299.20	C20H26O2	$[M+H]^+$	0.33	3
		27.51	299.20	C20H26O2	$[M+H]^+$	0.33	3
	Mono-oxygenated Diterpene (Isomer 1-7)	20.03	283.20	C20H26O	$[M+H]^+$	1.55	3
		21.90	283.20	C20H26O	$[M+H]^+$	0.38	3
		23.33	283.20	C20H26O	$[M+H]^+$	0.28	3
		19.25	283.20	C20H26O	$[M+H]^+$	0.23	3
		17.09	283.20	C20H26O	$[M+H]^+$	0.20	3
		21.18	283.20	C20H26O	$[M+H]^+$	0.18	3
		21.58	283.20	C20H26O	$[M+H]^+$	0.15	3
	Tri-oxygenated Diterpene (Isomer 1-6)	18.28	317.21	C20H28O3	$[M+H]^+$	1.20	3
		27.03	317.21	C20H28O3	$[M+H]^+$	0.53	3
		28.90	317.21	C20H28O3	$[M+H]^+$	0.41	3
		18.60	317.21	C20H28O3	$[M+H]^+$	0.37	3
		18.75	317.21	C20H28O3	$[M+H]^+$	0.18	3
		20.27	317.21	C20H28O3	$[M-H]^-$	0.11	3
	Diterpenoid (Isomer 1-3)	17.10	319.23	C20H30O3	$[M+H]^+$	0.94	3

		20.46	319.23	C20H30O3	[M+H]+	0.35	3
		21.68	317.23	C20H30O3	[M+H]+	0.13	3
	Unidentified Diterpene hydrocarbon (Isomers 1-5)	14.92	271.24	C20H30	[M+H]+	0.84	3
		27.59	271.24	C20H30	[M+H]+	0.40	3
		29.11	271.24	C20H30	[M+H]+	0.24	3
		23.80	271.24	C20H30	[M+H]+	0.23	3
		28.03	271.24	C20H30	[M+H]+	0.18	3
	Spatane ketone (Isomers 1-2)	29.50	287.24	C20H30O	[M+H]+	0.57	3
		23.40	287.24	C20H30O	[M+H]+	0.10	3
		27.45	287.24	C20H30O	[M+H]+	0.13	3
	Diene-alcohol (Isomers 1-3)	23.88	287.24	C20H30O	[M+H]+	0.35	3
		20.43	287.24	C20H30O	[M+H]+	0.23	3
		18.87	287.24	C20H30O	[M+H]+	0.16	3
		19.89	287.24	C20H30O	[M+H]+	0.13	3
	Mono-oxygenated Diterpene	18.28	285.22	C20H28O	[M+H]+	0.67	3
	Diterpenoid	22.73	301.22	C20H28O2	[M+H]+	0.67	3
	4-OxoDiterpenoid	13.94	315.20	C20H26O3	[M+H]+	0.22	3
	Diterpenoid Isomer	15.98	297.18	C20H24O2	[M+H]+	0.50	3
	Unidentified diterpenoid	17.05	331.19	C20H28O4	[M-H]-	0.10	3
**Functionalized Diterpenoids (Acetates & Algal Structural Clusters)** *This group contains diterpenoids heavily structuralized via custom enzymatic pathways of brown algae—namely mono-, di-, and tri-acetal/ester adaptations used for advanced herbivore defense shields*	Diterpene Diacetate (Isomer 1-2)	21.98	420.27	C24H34O5	[M+NH4]+	0.99	3
		22.45	420.27	C24H34O5	[M+NH4]+	0.22	3
	Diterpene Triacetate (Isomer 1-2)	21.16	480.29	C26H38O7	[M+NH4]+	0.98	3
		21.41	480.30	C26H38O7	[M+NH4]+	0.38	3
	4β-acetoxydictyodial A (Isomers 1-5)	22.74	378.26	C22H32O4	[M+NH4]+	0.44	2
		19.06	361.24	C22H32O4	[M+H]+	0.29	2
		22.02	361.24	C22H32O4	[M+H]+	0.16	2
		20.02	361.24	C22H32O4	[M+H]+	0.15	2
		19.83	361.24	C22H32O4	[M+H]+	0.11	2
	Rugukadiol A	19.07	438.28	C24H36O6	[M+NH4]+	0.37	2

	Secospatane monoacetates (Isomer 1-2)	21.10<br>20.52	359.22<br>359.22	C22H30O4<br>C22H30O4	$[M+H]^+$<br>$[M+H]^+$	0.35<br>0.19	3<br>3
	Polyoxygenated Diterpene Acetate	20.04	423.24	C23H36O7	$[M-H]^-$	0.20	3
	Diterpene monoacetate isomer (Isomer 1-2)	15.96<br>16.57	357.21<br>357.21	C22H28O4<br>C22H28O4	$[M+H]^+$<br>$[M+H]^+$	0.15<br>0.11	3<br>3
	Rugukamural C	18.65	333.21	C20H30O4	$[M-H]^-$	0.13	2
	Putative Acetylated Diterpene (Isomers 1-2)	20.02<br>21.99	342.23<br>342.23	C22H30O3<br>C22H30O3	$[M+H]^+$<br>$[M+H]^+$	1.76<br>0.64	3<br>3
	Acetylated Diterpenoid (Isomer 1-2)	19.31<br>18.94	358.24<br>358.24	C22H31NO3<br>C22H31NO3	$[M+H]^+$<br>$[M+H]^+$	0.25<br>0.17	3<br>3
	Diterpenoid Isomer	22.57	329.21	C21H28O3	$[M+H]^+$	0.42	3
**Fatty Acids & Oxylipins (Primary/Signaling Lipids)** *Conserved primary metabolic chains alongside hydroperoxidized structural derivatives (oxylipins) generated naturally under mechanical stress or defense pathways*	Palmitelaidic acid	28.64	253.22	C16H30O2	$[M-H]^-$	1.59	2
	Oleic acid	27.94	281.25	C18H34O2	$[M-H]^-$	1.56	2
	Palmitic Acid	28.76	255.23	C16H32O2	$[M-H]^-$	1.53	2
	Arachidonic acid	29.05	303.23	C20H32O2	$[M-H]^-$	1.41	2
	Myristic acid	28.29	227.20	C14H28O2	$[M-H]^-$	1.16	2
	Eicosapentaenoic acid	24.53	303.23	C20H30O2	$[M+H]^+$	0.79	2
	Linolenelaidic acid	26.30	277.22	C18H30O2	$[M-H]^-$	0.22	2
	(E)-parinaric acid	23.79	275.20	C18H28O2	$[M-H]^-$	0.46	2
	9,10-epoxy-12-octadecenoic acid	20.53	295.23	C18H32O3	$[M-H]^-$	0.46	2
	12-HETE (Algal Oxylipin)	20.53	319.23	C20H32O3	$[M-H]^-$	0.19	2
	Docosatetraenoic acid	25.37	331.27	C22H36O2	$[M-H]^-$	0.18	2
**Sesquiterpenoids & Norsesquiterpenoids (C14–C15 Volatiles)** *Shorter chain defensive terpenoid units, famous across Dictyotaceae seaweeds for volatile antibiotic and anti-fouling activities,*	Sesquiterpenoid Isomer (Isomer 1-4)	21.10<br>18.75<br>20.52<br>21.77	203.14<br>203.14<br>203.14<br>203.14	C14H18O<br>C14H18O<br>C14H18O<br>C14H18O	$[M+H]^+$<br>$[M+H]^+$<br>$[M+H]^+$<br>$[M+H]^+$	0.18<br>0.55<br>0.19<br>0.17	3<br>3<br>3<br>3
	Oxygenated Norsesquiterpenoid (Isomer 1-2)	16.58<br>15.96	201.13<br>201.13	C14H16O<br>C14H16O	$[M+H]^+$<br>$[M+H]^+$	0.10<br>0.14	3<br>3
	Sesquiterpene hydrocarbon	21.10	185.13	C14H16	$[M+H]^+$	0.11	3

	Dictyolactam Acetate (Isomer 1-2)	18.74	374.23	C22H31NO4	$[M+H]^+$	0.22	2
		18.08	374.23	C22H31NO4	$[M+H]^+$	1.41	2
	Nitrogenous Diterpene	16.65	332.22	C20H29NO3	$[M+H]^+$	0.10	3
	Nitrogenous Diterpene	23.41	296.20	C20H25NO	$[M+H]^+$	1.03	3
	Putative Nitrogenous Lipid	21.62	318.28	C21H35NO	$[M+H]^+$	0.29	3
	Putative Alkaloid Diterpene	18.87	298.22	C20H27NO	$[M+H]^+$	0.48	3
**Nitrogenous Terpenoids & Marine Alkaloids** *Secondary amine or lactam adaptations fused onto standard terpene branches. This core profile represents an evolutionary escalation in seaweed defensive weapons*	Putative Marine Alkaloid	14.88	314.21	C20H27NO2	$[M+H]^+$	0.11	3
	Chlorophyll Degradation (Fragment)	24.71	593.27	C35H36N4O5	$[M+H]^+$	0.10	3
	Ketocarotenoid (Isomer 1-2)	27.04	597.39	C40H52O4	$[M+H]^+$	0.26	2
		28.31	597.39	C40H52O4	$[M+H]^+$	0.18	2
	Unidentified Polyphenolic Complex	23.80	667.34	C40H48N2O7	$[M-H]^-$	0.15	4
	Unidentified Complex Polyether	26.67	791.50	C44H72O12	$[M-H]^-$	0.17	4
	Flavocristamide A	28.64	616.46	C34H67NO6S	$[M-H]^-$	0.15	3
**High-MW Biomass, Pigments, & Degradation Derivatives** *Heavy compound arrays originating from macroalgal structural pigments or macrocyclic ring-shatters of light-harvesting complex molecules*	β-Naphthol ethyl ether	21.11	173.10	C12H12O	$[M+H]^+$	0.22	4
	Linear Alkylbenzene Sulfonate	19.34	325.19	C18H30O3S	$[M-H]^-$	0.21	2
	Ammonium Oxalate Matrix	0.24	123.04	C2H6N2O4	$[M+H]^+$	0.18	4
	Terpenyl Nucleoside Analog	22.72	383.22	C19H30N2O6	$[M+H]^+$	0.11	4
	Chromen-octadecadienoate	28.89	597.39	C35H52N2O6	$[M+H]^+$	0.11	4
**Analytical Artifacts & Environmental Contaminants** *This section aggregates analytical noise from injection boundaries or human pollution footprints collected alongside sampling matrices*	Phepropeptin B	29.53	717.43	C40H56N6O6	$[M+H]^+$	0.11	4
	Pyridinediyl Decanone	24.72	388.32	C25H41NO2	$[M+H]^+$	0.11	4
	Pimara-dienyl derivative	21.41	418.26	C24H35NO5	$[M+H]^+$	0.10	4

Table S3. ***Dictyota dichotoma*** **Metabolomic Profile**

Compound Family	Tentative identification	RT (min)	m/z	Molecular Formula	Ionization Mode	Rel. Area (%)	MSI Level
**Diterpenoids**	Dictyolactam Acetate Isomer	18.58	374.23	C22H31NO4	$[M+H]^+$	7.09	3
	Di-oxygenated Diterpene	21.10	299.20	C20H26O2	$[M+H]^+$	6.26	3
	Di-oxygenated Diterpene	20.49	299.20	C20H26O2	$[M+H]^+$	3.64	3
	(3R,5R,7S,10S,11E,13S)-10-Hydroxy-3,6,6,10,16-pentamethyl-14-azatricyclo[11,2,1,0~5,7~]hexadeca-1(16),11-diene-2,15-dione	16.61	332.22	C20H29NO3	$[M+H]^+$	2.12	2
	Acetoxyditerpene	19.04	358.24	C22H28O3	$[M+NH4]^+$	1.69	3
	Nitrogenous Diterpenoid	23.41	296.20	C20H25NO	$[M+H]^+$	1.58	3
	Di-oxygenated Diterpene	21.76	299.20	C20H26O2	$[M+H]^+$	1.21	3
	Unidentified diterpene	27.59	271.24	C20H30	$[M+H]^+$	0.98	4
	Diterpene Diacetate	21.97	420.27	C24H34O5	$[M+NH4]^+$	0.93	3
	Diterpene Triacetate	21.17	480.29	C26H38O7	$[M+NH4]^+$	0.81	3
	Diterpenoid Isomer	15.98	297.18	C20H24O2	$[M+H]^+$	0.79	3
	Di-oxygenated Diterpene	23.04	299.20	C20H26O2	$[M+H]^+$	0.62	3
	Nitrogenous Diterpenoid	27.44	338.21	C22H27NO2	$[M+H]^+$	0.59	3
	4β-acetoxydictyodial A	19.07	361.24	C22H32O4	$[M+H]^+$	0.50	2
	Diterpenoid Isomer	22.57	329.21	C21H28O3	$[M+H]^+$	0.44	3
	diterpene monoacetate isomer	15.96	357.21	C22H28O4	$[M+H]^+$	0.43	3
	Diterpene Triacetate	21.42	480.30	C26H38O7	$[M+NH4]^+$	0.43	3
	Unidentified diterpene	29.11	271.24	C20H30	$[M+H]^+$	0.40	4
	diterpene monoacetate isomer	21.10	359.22	C22H30O4	$[M+H]^+$	0.38	3
	diterpene monoacetate isomer	20.50	359.22	C22H30O4	$[M+H]^+$	0.37	3
	Di-oxygenated Diterpene	29.25	299.20	C20H26O2	$[M+H]^+$	0.37	3
	Methylated Homoditerpenoid Isomer 1	20.01	347.22	C21H30O4	$[M+H]^+$	0.36	3
	4-OxoDictyoxetane	19.04	313.18	C20H26O3	$[M-H]^-$	0.36	2
	Hydroxy-Dictyolactam Acetate	13.26	390.23	C22H31NO5	$[M+H]^+$	0.35	3
	Diterpenoid Isomer	22.05	329.21	C21H28O3	$[M+H]^+$	0.34	3

Tri-oxygenated Diterpene	18.28	317.21	C20H28O3	$[M+H]^+$	0.33	3
Diterpenoid Isomer	16.59	297.18	C20H24O2	$[M+H]^+$	0.32	3
Nitrogenous Diterpenoid	21.85	296.20	C20H25NO	$[M+H]^+$	0.31	3
Dictyoxetane	19.80	301.22	C20H28O2	$[M+H]^+$	0.29	2
Mono-oxygenated Diterpene	20.05	285.22	C20H28O	$[M+H]^+$	0.29	3
Nitrogenous Diterpenoid	25.49	338.21	C22H27NO2	$[M+H]^+$	0.29	3
Mono-oxygenated Diterpene	23.45	285.22	C20H28O	$[M+H]^+$	0.28	3
Diterpenoid Isomer	22.57	297.18	C20H24O2	$[M+H]^+$	0.28	3
Dictyolactam Acetate	18.17	356.22	C22H29NO3	$[M+H]^+$	0.28	2
Di-oxygenated Diterpene	22.16	299.20	C20H26O2	$[M+H]^+$	0.27	3
Dictyoxetane	22.08	301.22	C20H28O2	$[M+H]^+$	0.26	2
4-OxoDictyoxetane	18.12	315.20	C20H26O3	$[M+H]^+$	0.24	2
4-OxoDictyoxetane	17.28	315.20	C20H26O3	$[M+H]^+$	0.24	2
Unidentified diterpene	28.03	271.24	C20H30	$[M+H]^+$	0.24	4
Mono-oxygenated Diterpene	20.41	287.24	C20H30O	$[M+H]^+$	0.24	3
Mono-oxygenated Diterpene	23.13	285.22	C20H28O	$[M+H]^+$	0.23	3
4-OxoDictyoxetane	19.76	315.20	C20H26O3	$[M+H]^+$	0.22	2
Mono-oxygenated Diterpene	19.42	287.24	C20H30O	$[M+H]^+$	0.22	3
diterpene monoacetate isomer	16.58	357.21	C22H28O4	$[M+H]^+$	0.22	3
Diterpenoid Isomer	16.33	297.18	C20H24O2	$[M+H]^+$	0.21	3
Dictyoxetane	17.78	301.22	C20H28O2	$[M+H]^+$	0.19	2
Unidentified diterpene	29.50	271.24	C20H30	$[M+H]^+$	0.19	4
Diterpene Diacetate	22.45	420.27	C24H34O5	$[M+NH4]^+$	0.19	3
Diterpenoid Isomer 5	22.04	297.18	C20H24O2	$[M+H]^+$	0.18	3
4-OxoDictyoxetane	21.37	315.20	C20H26O3	$[M+H]^+$	0.18	2
Mono-oxygenated Diterpene	26.54	285.22	C20H28O	$[M+H]^+$	0.17	3
Diterpenoid Isomer 6	17.27	297.18	C20H24O2	$[M+H]^+$	0.17	3
Dictyolactam Acetate Isomer	20.44	374.23	C22H31NO4	$[M+H]^+$	0.17	3
Diterpenoid Isomer	21.27	329.21	C21H28O3	$[M+H]^+$	0.17	3
Aromatized Diterpene	14.13	295.17	C20H22O2	$[M+H]^+$	0.17	3

Norditerpene	20.01	287.20	C19H26O2	$[M+H]^+$	0.16	3
(2R,4R,8R,9S,13R)-2-Hydroxy-5,5,9-trimethyl-14-methylene-10,15-dioxotricyclo[11,2,1,0~4,9~] hexadec-1(16)-en-8-yl acetate	18.99	375.22	C22H30O5	$[M+H]^+$	0.15	2
Diterpenoid Isomer	20.01	329.21	C21H28O3	$[M+H]^+$	0.15	3
Mono-oxygenated Diterpene	18.88	287.24	C20H30O	$[M+H]^+$	0.15	3
Dictyoxetane	19.07	301.22	C20H28O2	$[M+H]^+$	0.15	2
Dictyodial	16.12	303.23	C20H30O2	$[M+H]^+$	0.15	2
Di-oxygenated Diterpene	18.28	299.20	C20H26O2	$[M+H]^+$	0.14	3
Diterpenoid Isomer	17.01	327.20	C21H26O3	$[M+H]^+$	0.14	3
Mono-oxygenated Diterpene	17.98	285.22	C20H28O	$[M+H]^+$	0.14	3
Aromatized Diterpene	16.71	295.17	C20H22O2	$[M+H]^+$	0.13	3
4-OxoDictyoxetane	20.68	315.20	C20H26O3	$[M+H]^+$	0.13	2
Mono-oxygenated Diterpene	20.65	287.24	C20H30O	$[M+H]^+$	0.12	3
Dictyoxetane	23.35	301.22	C20H28O2	$[M+H]^+$	0.12	2
Diterpenoid Isomer 7	19.71	297.18	C20H24O2	$[M+H]^+$	0.12	3
Highly Oxygenated Diterpenoid	14.15	373.20	C22H28O5	$[M+H]^+$	0.12	3
Dictyolactam Acetate	19.48	356.22	C22H29NO3	$[M+H]^+$	0.12	2
Dictyoxetane	20.53	301.22	C20H28O2	$[M+H]^+$	0.12	2
Di-oxygenated Diterpene	28.31	299.20	C20H26O2	$[M+H]^+$	0.11	3
Diterpenoid Isomer	18.84	297.18	C20H24O2	$[M+H]^+$	0.11	3
Isodictyodial	19.44	303.23	C20H30O2	$[M+H]^+$	0.11	2
Diterpenoid Isomer 9	18.13	297.18	C20H24O2	$[M+H]^+$	0.11	3
Methylated Homoditerpenoid Isomer 2	20.58	363.22	C21H30O5	$[M+H]^+$	0.11	3
4β-acetoxydictyodial A	19.80	361.24	C22H32O4	$[M+H]^+$	0.11	2
Mono-oxygenated Diterpene	21.99	285.22	C20H28O	$[M+H]^+$	0.11	3
(2R,4R,8R,9S,13R)-2-Hydroxy-5,5,9-trimethyl-14-methylene-10,15-dioxotricyclo[11,2,1,0~4,9~] hexadec-1(16)-en-8-yl acetate	19.32	375.22	C22H30O5	$[M+H]^+$	0.11	2
Dictyoxetane	19.95	301.22	C20H28O2	$[M+H]^+$	0.11	2

	Diterpenoid Isomer	22.87	329.21	$C_{21}H_{28}O_3$	$[M+H]^+$	0.11	3
**Fatty Acids & Lipids**	Palmitic Acid	28.72	255.23	$C_{16}H_{32}O_2$	$[M-H]^-$	1.51	2
	Oleic acid	27.88	281.25	$C_{18}H_{34}O_2$	$[M-H]^-$	1.44	2
	Palmitelaidic acid	28.70	253.22	$C_{16}H_{30}O_2$	$[M-H]^-$	0.97	2
	Myristic acid	28.34	227.20	$C_{14}H_{28}O_2$	$[M-H]^-$	0.95	2
	Arachidonic acid	29.07	303.23	$C_{20}H_{32}O_2$	$[M-H]^-$	0.87	2
	Eicosapentanoic acid	25.54	301.22	$C_{20}H_{30}O_2$	$[M-H]^-$	0.56	2
	9,10-epoxy-12-octadecenoic acid	20.52	295.23	$C_{18}H_{32}O_3$	$[M-H]^-$	0.34	2
	Palmitelaidic acid	29.48	253.22	$C_{16}H_{30}O_2$	$[M-H]^-$	0.26	2
	12-HETE	20.52	319.23	$C_{20}H_{32}O_3$	$[M-H]^-$	0.21	2
	Linolenelaidic acid	26.32	277.22	$C_{18}H_{30}O_2$	$[M-H]^-$	0.11	2
	Arachidonic acid	28.07	303.23	$C_{20}H_{32}O_2$	$[M-H]^-$	0.11	2
	9-KODE	19.68	293.21	$C_{18}H_{30}O_3$	$[M-H]^-$	0.11	2
	(E)-parinaric acid	23.79	275.20	$C_{18}H_{28}O_2$	$[M-H]^-$	0.10	2
**Phenolic Lipids & Benzenoids**	2-Hydroxy-6-[(8E,11E)-8,11,14-pentadecatrien-1-yl]benzoic acid (Isomer 1-7)	21.17	343.23	$C_{22}H_{30}O_3$	$[M+H]^+$	0.47	2
		21.99	343.23	$C_{22}H_{30}O_3$	$[M+H]^+$	0.40	2
		19.80	343.23	$C_{22}H_{30}O_3$	$[M+H]^+$	0.28	2
		21.43	343.23	$C_{22}H_{30}O_3$	$[M+H]^+$	0.14	2
		19.28	343.23	$C_{22}H_{30}O_3$	$[M+H]^+$	0.13	2
		16.04	279.17	$C_{20}H_{22}O$	$[M+H]^+$	0.13	2
		23.33	360.25	$C_{22}H_{30}O_3$	$[M+NH4]^+$	0.12	2
	β-Naphthol ethyl ether	21.10	173.10	$C_{12}H_{12}O$	$[M+H]^+$	0.26	2
	2-Phenylpent-4-enal	1.97	161.10	$C_{11}H_{12}O$	$[M+H]^+$	0.20	2
	2-Methoxynaphthalene	15.95	159.08	$C_{11}H_{10}O$	$[M+H]^+$	0.14	2
	β-Naphthol ethyl ether	20.50	173.10	$C_{12}H_{12}O$	$[M+H]^+$	0.14	2
**Sesquiterpenoids & Norisoprenoids**	Sesquiterpenoid Isomer	21.10	203.14	$C_{14}H_{18}O$	$[M+H]^+$	0.65	3
	dehydroretinaldehyde	19.80	283.21	$C_{20}H_{26}O$	$[M+H]^+$	0.63	2
	dehydroretinaldehyde	21.91	283.21	$C_{20}H_{26}O$	$[M+H]^+$	0.43	2
	Oxygenated Norsesquiterpenoid	15.96	201.13	$C_{14}H_{16}O$	$[M+H]^+$	0.39	3
	Sesquiterpenoid Isomer	20.49	203.14	$C_{14}H_{18}O$	$[M+H]^+$	0.34	3

	dehydroretinaldehyde	23.34	283.21	C20H26O	$[M+H]^+$	0.27	2
	Sesquiterpenoid Isomer	21.10	185.13	C14H16	$[M+H]^+$	0.26	3
	Zuretinol acetate	27.85	327.23	C22H32O2	$[M-H]^-$	0.19	2
	Norisoprenoid Hydrocarbon	15.95	173.13	C13H16	$[M+H]^+$	0.16	3
	Oxygenated Norsesquiterpenoid	16.59	201.13	C14H16O	$[M+H]^+$	0.15	3
	Sesquiterpenoid Isomer	20.50	185.13	C14H16	$[M+H]^+$	0.15	3
	Norisoprenoid Hydrocarbon	27.58	173.13	C13H16	$[M+H]^+$	0.11	3
	dehydroretinaldehyde	20.96	283.20	C20H26O	$[M+H]^+$	0.11	2
	Sesquiterpenoid Isomer	21.76	203.14	C14H18O	$[M+H]^+$	0.11	3
**Alkaloids & Nitrogenous**	(1S,2S,3S,6R,7S,14S)-7-Ethyl-3-methyl-14-[(2S,4S)-4-methyl-5-oxotetrahydro-2-furanyl]-5-oxa-13-azatricyclo[11,2,1,0~2,6~]hexadecane-4,8,16-trione	14.03	406.22	C22H31NO6	$[M+H]^+$	0.23	2
	(1α,9ξ,13α,14β,16β,17ξ)-20-Ethyl-1,4-dimethylaconitane-8,10,14,16-tetrol	20.53	378.26	C22H35NO4	$[M+H]^+$	0.13	2
	(1S)-1-[4-(2-{[(1S)-6,7-Dimethoxy-2-methyl-1,2,3,4-tetrahydro-1-isoquinolinyl]methyl}-4,5-dimethoxyphenoxy)benzyl]-6,7-dimethoxy-2-methyl-1,2,3,4-tetrahydroisoquinoline	23.06	667.34	C40H48N2O7	$[M-H]^-$	0.13	2
	Rubrumazine A	23.11	440.26	C25H33N3O4	$[M+H]^+$	0.11	2
**Other Polyketides, Miscellaneous &Putative Environmental / Contaminant Signals**	Dimethyl (2E)-3-methoxy-4-oxo-5-[(8E,11E)-8,11,14-pentadecatrien-1-yl]-2-hexenedioate	19.06	438.28	C24H36O6	$[M+NH4]^+$	0.82	2
	Dimethyl (2E)-3-methoxy-4-oxo-5-[(8E,11E)-8,11,14-pentadecatrien-1-yl]-2-hexenedioate	19.80	438.28	C24H36O6	$[M+NH4]^+$	0.20	2
	2-Dodecylbenzenesulfonic acid	19.34	325.19	C18H30O3S	$[M-H]^-$	0.17	2
	(−)-Centrolobine	14.10	313.18	C20H24O3	$[M+H]^+$	0.15	2
	2,5:3,4-Dianhydro-1,6-dideoxy-2-[(1E,3E,5E)-6-(4-methoxy-3,5-dimethyl-2-oxo-2H-pyran-6-yl)-2,4-dimethyl-1,3,5-heptatrien-1-yl]-4-methyl-D-altritol	20.12	401.23	C24H32O5	$[M+H]^+$	0.13	2
	cholesteryl sulfate	29.09	465.31	C27H46O4S	$[M-H]^-$	0.11	2
	Androsterone glucuronide	19.80	465.25	C25H38O8	$[M-H]^-$	0.11	2

Table S4. ***Ericaria selaginoides*** **Metabolomic Profile**

Compound Family	Tentative Identification	Rt (min)	m/z	Molecular Formula	Ionization Mode	Rel. Area (%)	MSI level
**Alkaloids & Nitrogenous**	Polyamine/Indole deriv.	24.46	423.25	C28 H30 N4	$[M+H]^+$	5.17	3
	Isomers 1-2	21.19	423.25	C28 H30 N4	$[M+H]^+$	3.32	3
	Alkaloid	21.17	458.29	C27 H39 N O5	$[M+H]^+$	1.98	3
	Polycyclic Alkaloid	26.22	423.25	C23 H30 N6 O2	$[M+H]^+$	1.40	3
	Alkaloid	18.60	460.31	C27 H41 N O5	$[M+H]^+$	0.75	3
	Peptidic Alkaloid	21.18	463.24	C25 H30 N6 O3	$[M+H]^+$	0.72	3
	Polycyclic Alkaloid	20.54	425.27	C23 H32 N6 O2	$[M+H]^+$	0.68	3
	Nitrogenous derivative	24.53	463.24	C24 H34 N2 O7	$[M+H]^+$	0.64	3
	Polycyclic Alkaloid	20.53	474.32	C24 H39 N7 O3	$[M+H]^+$	0.57	3
	Nitrogenous derivative	24.12	501.29	C30 H38 N4 O3	$[M-H]^-$	0.55	3
	Peptidic Alkaloid	16.30	483.27	C25 H34 N6 O4	$[M+H]^+$	0.55	3
	Alkaloid	24.41	458.29	C27 H39 N O5	$[M+H]^+$	0.54	3
	Alkaloid	23.50	442.29	C27 H39 N O4	$[M+H]^+$	0.51	3
	Polyamine/Indole deriv.	23.62	423.25	C28 H30 N4	$[M+H]^+$	0.51	3
	Nitrogenous derivative	18.61	465.26	C24 H36 N2 O7	$[M+H]^+$	0.45	3
	Alkaloid	20.80	458.29	C27 H39 N O5	$[M+H]^+$	0.42	3
	Polycyclic Alkaloid	19.75	458.29	C23 H35 N7 O3	$[M+H]^+$	0.41	3
	Nitrogenous derivative	18.47	481.26	C24 H36 N2 O8	$[M+H]^+$	0.34	3
	Nitrogenous derivative	19.20	549.29	C34 H38 N4 O3	$[M-H]^-$	0.32	3
	Polyamine/Indole deriv.	21.22	441.27	C28 H34 N4 O	$[M-H]^-$	0.32	3
	Polycyclic Alkaloid	26.11	439.28	C24 H34 N6 O2	$[M+H]^+$	0.31	3
	Polycyclic Alkaloid	20.97	563.30	C31 H36 N10 O	$[M-H]^-$	0.30	3
	Nitrogenous derivative	27.09	463.24	C24 H34 N2 O7	$[M+H]^+$	0.30	3
	Polycyclic Alkaloid	17.71	439.28	C24 H34 N6 O2	$[M+H]^+$	0.30	3
	Polycyclic Alkaloid	21.46	441.26	C23 H32 N6 O3	$[M+H]^+$	0.26	3
	Peptidic Alkaloid	17.82	497.29	C26 H36 N6 O4	$[M+H]^+$	0.26	3
	Peptidic Alkaloid	17.57	465.26	C25 H32 N6 O3	$[M+H]^+$	0.26	3

Peptidic Alkaloid	20.53	479.28	C26 H34 N6 O3	$[M+H]^+$	0.25	3
Polyamine deriv.	18.05	497.29	C27 H32 N10	$[M+H]^+$	0.24	3
Peptidic Alkaloid	19.75	463.24	C25 H30 N6 O3	$[M+H]^+$	0.24	3
Polyamine deriv.	22.98	439.28	C29 H34 N4	$[M+H]^+$	0.24	3
Polycyclic Alkaloid	16.30	443.28	C23 H34 N6 O3	$[M+H]^+$	0.22	3
Peptidic Alkaloid	17.30	467.28	C25 H34 N6 O3	$[M+H]^+$	0.21	3
Polyamine deriv.	18.47	441.26	C28 H32 N4 O	$[M+H]^+$	0.20	3
Nitrogenous derivative	21.45	481.26	C24 H36 N2 O8	$[M+H]^+$	0.20	3
Polycyclic Alkaloid	20.29	460.31	C23 H37 N7 O3	$[M+H]^+$	0.20	3
Polycyclic Alkaloid	20.14	439.28	C24 H34 N6 O2	$[M+H]^+$	0.19	3
Peptidic Alkaloid	18.57	459.28	C16 H40 N6 O9	$[M-H]^-$	0.18	3
Polyamine deriv.	21.26	425.27	C28 H32 N4	$[M+H]^+$	0.17	3
Polycyclic Alkaloid	17.80	492.33	C24 H41 N7 O4	$[M+H]^+$	0.17	3
Polycyclic Alkaloid	20.71	407.26	C23 H30 N6 O	$[M+H]^+$	0.17	3
Polyamine deriv.	16.60	565.28	C35 H34 N8	$[M-H]^-$	0.16	3
Polycyclic Alkaloid	22.65	439.28	C24 H34 N6 O2	$[M+H]^+$	0.16	3
Polycyclic Alkaloid	17.82	457.29	C24 H36 N6 O3	$[M+H]^+$	0.16	3
Peptidic Alkaloid	19.70	465.26	C25 H32 N6 O3	$[M+H]^+$	0.15	3
Peptidic Alkaloid	22.68	485.26	C17 H38 N6 O10	$[M-H]^-$	0.14	3
Polyamine deriv.	25.43	439.25	C28 H32 N4 O	$[M-H]^-$	0.14	3
Peptidic Alkaloid	20.29	465.26	C25 H32 N6 O3	$[M+H]^+$	0.14	3
Nitrogenous derivative	20.54	455.28	C29 H36 N4 O	$[M-H]^-$	0.13	3
Alkaloid	18.95	490.32	C28 H43 N O6	$[M+H]^+$	0.13	3
Alkaloid	19.22	474.32	C28 H43 N O5	$[M+H]^+$	0.13	3
Polycyclic Alkaloid	28.24	441.26	C23 H32 N6 O3	$[M+H]^+$	0.12	3
Peptidic Alkaloid	16.91	481.26	C25 H32 N6 O4	$[M+H]^+$	0.12	3
Polyamine/Indole deriv.	16.93	423.25	C28 H30 N4	$[M+H]^+$	0.12	3
Polycyclic Alkaloid	24.16	447.25	C25 H30 N6 O2	$[M+H]^+$	0.12	3
Polyamine deriv.	25.14	439.28	C29 H34 N4	$[M+H]^+$	0.12	3
Nitrogenous derivative	19.42	455.28	C29 H36 N4 O	$[M-H]^-$	0.11	3

	Peptidic Alkaloid	20.79	463.24	C25 H30 N6 O3	$[M+H]^+$	0.11	3
	Polyamine deriv.	22.63	439.25	C28 H32 N4 O	$[M-H]^-$	0.11	3
	Nitrogenous derivative	18.80	565.28	C34 H38 N4 O4	$[M-H]^-$	0.11	3
	Polyamine deriv.	23.01	425.27	C28 H32 N4	$[M+H]^+$	0.11	3
	Small Amine	1.68	128.14	C8 H17 N	$[M+H]^+$	0.11	3
	Polyamine deriv.	28.55	425.27	C28 H32 N4	$[M+H]^+$	0.10	3
	Polycyclic Alkaloid	17.44	460.30	C23 H37 N7 O3	$[M+H]^+$	0.10	3
	Alkaloid	20.41	444.31	C27 H41 N O4	$[M+H]^+$	0.10	3
	Polycyclic Alkaloid	20.52	547.27	C30 H32 N10 O	$[M-H]^-$	0.10	3
	Nitrogenous derivative	17.30	443.28	C28 H36 N4 O	$[M-H]^-$	0.10	3
	Polyamine deriv.	22.79	531.27	C30 H30 N10	$[M+H]^+$	0.10	3
	Alkaloid	20.89	474.32	C28 H43 N O5	$[M+H]^+$	0.09	3
	Nitrogenous derivative	22.07	481.26	C24 H36 N2 O8	$[M+H]^+$	0.09	3
	Alkaloid	24.74	446.33	C27 H43 N O4	$[M+H]^+$	0.09	3
	Polycyclic Alkaloid	16.21	478.32	C23 H39 N7 O4	$[M+H]^+$	0.09	3
	Polyamine deriv.	19.11	441.30	C29 H36 N4	$[M+H]^+$	0.09	3
	Polyamine/Indole deriv.	16.50	423.25	C28 H30 N4	$[M+H]^+$	0.09	3
	Nitrogenous derivative	25.44	463.24	C24 H34 N2 O7	$[M+H]^+$	0.09	3
	Polyamine deriv.	23.19	441.26	C28 H32 N4 O	$[M+H]^+$	0.09	3
	Nitrogenous derivative	23.03	487.27	C29 H36 N4 O3	$[M-H]^-$	0.09	3
	Nitrogenous derivative	17.86	455.28	C29 H34 N4 O	$[M+H]^+$	0.09	3
	Nitrogenous derivative	23.89	447.25	C24 H34 N2 O6	$[M+H]^+$	0.08	3
	Nitrogenous derivative	18.63	565.28	C34 H38 N4 O4	$[M-H]^-$	0.08	3
	Polyamine deriv.	26.20	455.24	C24 H26 N10	$[M+H]^+$	0.08	3
	Polyamine deriv.	24.89	425.27	C28 H32 N4	$[M+H]^+$	0.08	3
	Alkaloid	19.70	460.31	C28 H37 N5 O	$[M+H]^+$	0.08	3
**Sterols, Terpenoids & Oxylipins**	Seco-sterol (Isomer 1-7)	26.63	439.25	C27 H36 O5	$[M-H]^-$	2.49	3
		24.53	441.26	C27 H36 O5	$[M+H]^+$	0.54	3
		26.24	441.26	C27 H36 O5	$[M+H]^+$	0.26	3
		27.08	441.26	C27 H36 O5	$[M+H]^+$	0.15	3

	17.58	441.26	C27 H36 O5	$[M+H]^+$	0.14	3
	18.70	441.26	C27 H36 O5	$[M+H]^+$	0.10	3
	18.23	441.26	C27 H36 O5	$[M+H]^+$	0.10	3
C28 Sterol derivative (Isomer 1-3)	23.89	439.28	C28 H38 O4	$[M+H]^+$	1.21	3
	29.99	439.28	C28 H38 O4	$[M+H]^+$	0.61	3
	20.54	439.28	C28 H38 O4	$[M+H]^+$	0.57	3
Highly oxygenated sterol (Isomer 1-5)	24.12	455.28	C28 H40 O5	$[M-H]^-$	1.03	3
	18.14	457.29	C28 H40 O5	$[M+H]^+$	0.19	3
	21.46	457.26	C27 H38 O6	$[M-H]^-$	0.15	3
	22.64	455.28	C28 H40 O5	$[M-H]^-$	0.15	3
	26.11	457.29	C28 H40 O5	$[M+H]^+$	0.10	3
Methoxycystoketal quinone (Isomer 1-3)	26.61	423.25	C27 H34 O4	$[M+H]^+$	1.82	3
	27.08	423.25	C27 H34 O4	$[M+H]^+$	1.08	3
	20.74	439.25	C27 H34 O5	$[M+H]^+$	0.13	3
Oxygenated Terpenoid	20.99	487.27	C28 H40 O7	$[M-H]^-$	0.61	3
Heavy oxygenated terpene	21.69	549.28	C33 H40 O7	$[M+H]^+$	0.57	3
Hydroxy-sterol (Isomer 1-11)	18.62	425.27	C27 H36 O4	$[M+H]^+$	1.80	3
	20.30	425.27	C27 H36 O4	$[M+H]^+$	1.14	3
	16.30	425.27	C27 H36 O4	$[M+H]^+$	1.13	3
	24.26	425.27	C27 H36 O4	$[M+H]^+$	0.98	3
	20.98	425.27	C27 H36 O4	$[M+H]^+$	0.73	3
	23.68	425.27	C27 H36 O4	$[M+H]^+$	0.59	3
	19.70	425.27	C27 H36 O4	$[M+H]^+$	0.47	3
	23.85	425.27	C27 H36 O4	$[M+H]^+$	0.45	3
	19.38	425.27	C27 H36 O4	$[M+H]^+$	0.39	3
	15.94	425.27	C27 H36 O4	$[M+H]^+$	0.15	3
	22.82	425.27	C27 H36 O4	$[M+H]^+$	0.13	3
Dehydro-sterol (Isomer 1-4)	19.92	407.26	C27 H34 O3	$[M+H]^+$	0.41	3
	21.90	407.26	C27 H34 O3	$[M+H]^+$	0.40	3
	23.89	407.26	C27 H34 O3	$[M+H]^+$	0.19	3

		22.42	407.26	C27 H34 O3	[M+H]+	0.19	3
	Oxysterol (Isomer 1-5)	20.99	441.27	C27 H38 O5	[M-H]-	0.72	3
		18.62	441.27	C27 H38 O5	[M-H]-	0.49	3
		20.55	441.27	C27 H38 O5	[M-H]-	0.43	3
		17.58	441.27	C27 H38 O5	[M-H]-	0.38	3
		20.31	441.27	C27 H38 O5	[M-H]-	0.31	3
	Oxysterol derivative	16.29	459.28	C27 H40 O6	[M-H]-	0.31	3
	Deoxy-sterol (Isomer 1-3)	24.18	405.24	C27 H32 O3	[M+H]+	0.45	3
		25.43	405.24	C27 H32 O3	[M+H]+	0.29	3
		19.60	405.24	C27 H32 O3	[M+H]+	0.10	3
	C27 Sterol (Isomer 1-3)	17.34	427.28	C27 H38 O4	[M+H]+	0.35	3
		20.15	427.28	C27 H38 O4	[M+H]+	0.31	3
		20.69	427.28	C27 H38 O4	[M+H]+	0.21	3
	C28 Sterol	19.28	441.30	C28 H40 O4	[M+H]+	0.20	3
	C28 Sterol derivative	24.66	439.28	C28 H38 O4	[M+H]+	0.09	3
	Oxygenated Terpenoid (Isomer 1-4)	17.64	473.29	C28 H42 O6	[M-H]-	0.18	3
		24.51	485.26	C28 H38 O7	[M-H]-	0.15	3
		23.55	487.27	C28 H40 O7	[M-H]-	0.10	3
		18.13	455.28	C28 H38 O5	[M+H]+	0.09	3
	Cholest-type sterol	21.80	409.27	C27 H36 O3	[M+H]+	0.15	3
	Methoxycystoketal quinone	25.48	439.25	C27 H34 O5	[M+H]+	0.10	3
	Didehydro-sterol	21.80	391.26	C27 H34 O2	[M+H]+	0.10	3
	Sterol	24.68	411.29	C27 H38 O3	[M+H]+	0.09	2
**Fatty Acids & Lipids**	Stearidonic acid deriv.	26.38	277.22	C18 H30 O2	[M-H]-	0.34	2
	Palmitoleic acid deriv. (Isomer 1-2)	29.56	253.22	C16 H30 O2	[M-H]-	0.34	2
		28.74	253.22	C16 H30 O2	[M-H]-	0.24	2
	Hydroxy-eicosanoid	25.60	301.22	C20 H30 O2	[M-H]-	0.64	2
	Myristic acid deriv.	28.45	227.20	C14 H28 O2	[M-H]-	0.17	2
	Pentadecatrienoic acid deriv. (Isomer 1-2)	17.59	233.15	C15 H20 O2	[M+H]+	0.17	2
		19.75	233.15	C15 H20 O2	[M+H]+	0.15	2

	Pentadecadienoic acid deriv.	23.49	235.17	C15 H22 O2	$[M+H]^+$	0.16	2
	Terpenoid lipid	25.43	353.21	C23 H28 O3	$[M+H]^+$	0.15	3
**Halogenated & Sulfated**	Halogenated sterol	24.12	491.26	C28 H41 Cl O5	$[M-H]^-$	0.55	3
	Halogenated sterol	20.96	477.24	C27 H39 Cl O5	$[M-H]^-$	0.33	3
	Sulfated lipid	20.31	293.18	C14 H30 O4 S	$[M-H]^-$	0.27	3
	Sulfur-containing amine	17.97	265.15	C13 H22 N4 S	$[M-H]^-$	0.27	3
	Halogenated Alkaloid	16.29	495.25	C28 H37 Cl N4 O2	$[M-H]^-$	0.25	3
	Chloro-sulfated lipid	26.48	475.23	C24 H41 Cl O5 S	$[M-H]^-$	0.25	3
	Halogenated Alkaloid	26.44	441.22	C23 H29 Cl N6 O	$[M+H]^+$	0.24	3
	Sulfated oxylipin	19.77	325.19	C18 H30 O3 S	$[M-H]^-$	0.22	3
	Halogenated sterol	21.85	477.24	C27 H37 Cl O5	$[M+H]^+$	0.22	3
	Small Sulfur/Nitrogen	1.23	214.09	C10 H15 N O2 S	$[M+H]^+$	0.19	3
	Halogenated sterol deriv.	17.67	509.27	C28 H43 Cl O6	$[M-H]^-$	0.17	3
	Halogenated sterol deriv.	18.14	509.27	C28 H43 Cl O6	$[M-H]^-$	0.14	3
	Chloro-sulfated lipid	17.58	477.24	C24 H43 Cl O5 S	$[M-H]^-$	0.14	3
	Halogenated sterol	21.22	477.24	C27 H39 Cl O5	$[M-H]^-$	0.12	3
	Halogenated sterol	20.57	477.24	C27 H39 Cl O5	$[M-H]^-$	0.12	3
	Halogenated sterol	18.48	495.25	C27 H41 Cl O6	$[M-H]^-$	0.11	3
	Sulfated lipid	18.95	309.17	C14 H30 O5 S	$[M-H]^-$	0.11	3
	Sulfur-alkaloid	23.48	407.26	C19 H38 N2 O5 S	$[M+H]^+$	0.11	3
	Halogenated Alkaloid	22.64	491.26	C29 H37 Cl N4 O	$[M-H]^-$	0.11	3
	Halogenated Terpenoid	19.18	585.26	C33 H43 Cl O7	$[M-H]^-$	0.11	3
	Chloro-sulfated lipid	18.63	477.24	C24 H43 Cl O5 S	$[M-H]^-$	0.11	3
	Halogenated sterol	17.41	477.24	C27 H39 Cl O5	$[M-H]^-$	0.10	3
	Sulfated lipid	21.86	337.21	C16 H34 O5 S	$[M-H]^-$	0.10	3
	Halogenated sterol	19.75	475.23	C27 H37 Cl O5	$[M-H]^-$	0.10	3
	Poly-sulfur alkaloid	24.11	457.29	C17 H44 N8 S3	$[M+H]^+$	0.09	3
	Small sulfated amine	0.33	167.01	C3 H6 N2 O4 S	$[M+H]^+$	0.09	3
	Sulfur-alkaloid	19.40	443.28	C20 H38 N6 O3 S	$[M+H]^+$	0.09	3
	Disulfide/Terpene deriv.	23.55	455.28	C29 H44 S2	$[M-H]^-$	0.08	3

	Sulfated lipid	17.47	265.15	C12 H26 O4 S	$[M-H]^-$	0.08	3
	Sulfur-alkaloid	18.96	555.29	C27 H40 N8 O3 S	$[M-H]^-$	0.13	3
	Sulfated oxylipin	21.24	339.20	C19 H32 O3 S	$[M-H]^-$	0.13	3
**Phenolics & Small Molecules**	Phloroglucinol derivative	24.50	189.09	C12 H14 O2	$[M-H]^-$	0.24	3
	Phloroglucinol	1.90	127.04	C6 H6 O3	$[M+H]^+$	0.22	3
	Phenolic / Organic Acid	11.50	197.12	C11 H16 O3	$[M+H]^+$	0.18	3
	Phenolic derivative (Isomer 1-2)	17.58	177.09	C11 H12 O2	$[M+H]^+$	0.14	3
		20.14	177.09	C11 H12 O2	$[M+H]^+$	0.10	3
	Simple Phenolic (Isomer 1-8)	19.96	175.08	C11 H10 O2	$[M+H]^+$	0.23	3
		17.45	175.08	C11 H10 O2	$[M+H]^+$	0.18	3
		21.63	175.08	C11 H10 O2	$[M+H]^+$	0.15	3
		21.92	175.08	C11 H10 O2	$[M+H]^+$	0.14	3
		22.08	175.08	C11 H10 O2	$[M+H]^+$	0.11	3
		20.52	175.08	C11 H10 O2	$[M+H]^+$	0.10	3
		22.78	175.08	C11 H10 O2	$[M+H]^+$	0.09	3
		27.08	175.08	C11 H10 O2	$[M+H]^+$	0.08	3
	Small Organic Acid	1.65	185.07	C5 H12 O7	$[M+H]^+$	0.10	3

Table S5. ***Codium bursa*** **Metabolomic Profile**

Family	Tentative Identification	RT [min]	m/z	Molecular Formula	Ionization Mode	Relative Area (%)	MSI Level
**Meroterpenoids & Terpenoids**	Meroterpenoid derivative (Isomer 1-4)	21.44	423.25	C27 H34 O4	$[M+H]^+$	9.16	3
		26.17	423.25	C27 H34 O4	$[M+H]^+$	7.68	3
		22.44	423.25	C27 H34 O4	$[M+H]^+$	2.92	3
		24.42	423.25	C27 H34 O4	$[M+H]^+$	2.48	3
	Brevione A (Isomer 1-9)	25.50	425.27	C27 H36 O4	$[M+H]^+$	3.04	2
		21.08	425.27	C27 H36 O4	$[M+H]^+$	1.18	2
		18.77	425.27	C27 H36 O4	$[M+H]^+$	0.58	2
		27.52	425.27	C27 H36 O4	$[M+H]^+$	0.43	2
		29.83	425.27	C27 H36 O4	$[M+H]^+$	0.36	2
		17.69	425.27	C27 H36 O4	$[M+H]^+$	0.18	2
		26.59	425.27	C27 H36 O4	$[M+H]^+$	0.16	2
		16.21	425.27	C27 H36 O4	$[M+H]^+$	0.13	2
		24.18	425.27	C27 H36 O4	$[M+H]^+$	0.12	2
	Algal Meroterpenoid (Isomer 1-4)	20.56	441.30	C28 H40 O4	$[M+H]^+$	2.57	2
		25.52	441.30	C28 H40 O4	$[M+H]^+$	2.29	2
		17.43	441.30	C28 H40 O4	$[M+H]^+$	0.22	2
		23.88	441.30	C28 H40 O4	$[M+H]^+$	0.20	2
	(+)-Spiculoic acid A (Isomer 1-2)	23.86	409.27	C27 H36 O3	$[M+H]^+$	0.88	2
		23.24	409.27	C27 H36 O3	$[M+H]^+$	0.72	2
	Oxygenated Sesquiterpenoid (Isomer 1-2)	23.86	219.17	C15 H22 O	$[M+H]^+$	0.60	3
		23.24	219.17	C15 H22 O	$[M+H]^+$	0.52	3
	Meroterpenoid derivative (Suite B)	22.61	421.27	C28 H36 O3	$[M+H]^+$	0.27	3
	Algal Sesquiterpene Hydrocarbon (Isomer 1-2)	23.23	191.18	C14 H22	$[M+H]^+$	0.24	3
		23.86	191.18	C14 H22	$[M+H]^+$	0.16	3
	(+)-Spiculoic acid A (Isomer 1-2)	20.66	426.30	C27 H36 O3	$[M+NH4]^+$	0.21	2
		21.22	426.30	C27 H36 O3	$[M+NH4]^+$	0.14	2
	Sinubrasolide A (Isomer 1-2)	19.43	439.28	C28 H38 O4	$[M+H]^+$	0.21	2

		24.02	439.28	C28 H38 O4	$[M+H]^+$	0.16	2
	Makassaric acid	24.60	411.29	C27 H38 O3	$[M+H]^+$	0.18	2
	Linalyl propionate	16.56	228.20	C13 H22 O2	$[M+NH4]^+$	0.18	2
	Prenylated Chromanol	19.53	483.27	C29 H40 O6	$[M-H]^-$	0.17	2
	Chabrolonaphthoquinone A	27.59	421.24	C27 H32 O4	$[M+H]^+$	0.12	2
	Oxygenated Polycyclic Triterpenoid	29.82	423.29	C28 H38 O3	$[M+H]^+$	0.12	3
	Prenylated Polyphenol derivative	21.85	439.25	C27 H34 O5	$[M+H]^+$	0.10	2
**Fatty Acids & Oxylipins**	Palmitic acid (C16:0)	28.91	255.23	C16 H32 O2	$[M-H]^-$	5.52	2
	Oleic acid (C18:1)	28.11	281.25	C18 H34 O2	$[M-H]^-$	5.15	2
	Linolenelaidic acid (C18:3)	26.35	277.22	C18 H30 O2	$[M-H]^-$	3.31	2
	Palmitelaidic acid (C16:1)	29.53	253.22	C16 H30 O2	$[M-H]^-$	1.31	2
	Arachidonic acid (C20:4)	29.11	303.23	C20 H32 O2	$[M-H]^-$	0.75	2
	Hexadecatrienoic acid (C16:3)	22.53	249.19	C16 H26 O2	$[M-H]^-$	0.67	2
	Myristic acid (C14:0)	28.45	227.20	C14 H28 O2	$[M-H]^-$	0.55	2
	Palmitelaidic acid isomer	28.75	253.22	C16 H30 O2	$[M-H]^-$	0.53	2
	9,10-Epoxystearic acid (Isomer 1-6)	26.34	297.24	C18 H34 O3	$[M-H]^-$	0.42	2
		26.58	297.24	C18 H34 O3	$[M-H]^-$	0.28	2
		28.59	297.24	C18 H34 O3	$[M-H]^-$	0.14	2
		25.47	297.24	C18 H34 O3	$[M-H]^-$	0.13	2
		24.75	297.24	C18 H34 O3	$[M-H]^-$	0.11	2
		25.88	297.24	C18 H34 O3	$[M-H]^-$	0.11	2
	9-KODE	19.56	293.21	C18 H30 O3	$[M-H]^-$	0.32	2
	Stearic acid (C18:0)	26.14	283.26	C18 H36 O2	$[M-H]^-$	0.27	2
	9,10-Epoxy-12-octadecenoic acid	20.59	295.23	C18 H32 O3	$[M-H]^-$	0.19	2
		22.73	295.23	C18 H32 O3	$[M-H]^-$	0.14	2
	Phytosphingosine	25.32	336.31	C18 H38 O4	$[M+NH4]^+$	0.18	2
		25.03	319.28	C18 H38 O4	$[M+H]^+$	0.18	2
	Algal C17 Oxylipin derivative	17.20	293.18	C17 H26 O4	$[M-H]^-$	0.17	3
	Stearidonic acid (Algal C18:4 ω-3)	19.57	277.22	C18 H28 O2	$[M+H]^+$	0.16	2
	2-Linolenoylglycerol	19.84	353.27	C21 H36 O4	$[M+H]^+$	0.14	2

	Juniperic acid (Isomer 1-2)	26.32	271.23	C16 H32 O3	$[M-H]^-$	0.14	2
		28.60	271.23	C16 H32 O3	$[M-H]^-$	0.14	2
	Palmitelaidic acid isomer	29.18	253.22	C16 H30 O2	$[M-H]^-$	0.13	2
	Glyceryl 2-myristate	28.87	301.24	C17 H34 O4	$[M-H]^-$	0.12	2
	Hydroxy fatty acid	28.13	327.25	C19 H36 O4	$[M-H]^-$	0.11	3
**Pigments & Carotenoids**	Chlorophyll degradation product (Isomer 1-2)	24.81	593.27	C35 H36 N4 O5	$[M+H]^+$	1.99	3
		27.29	593.28	C35 H36 N4 O5	$[M+H]^+$	0.59	3
	Dehydro-carotenone macroalgal pigment (Isomer 1-2)	23.67	581.40	C40 H52 O3	$[M+H]^+$	1.17	3
		28.56	581.40	C40 H52 O3	$[M+H]^+$	0.63	3
	Apocarotenoid algal pigment	24.59	393.28	C27 H36 O2	$[M+H]^+$	0.12	3
**Phospholipid**	1-Stearoyl-sn-glycero-3-phosphate (LPA) (Isomer 1-2)	16.88	439.28	C21 H43 O7 P	$[M+H]^+$	2.26	2
		16.44	439.28	C21 H43 O7 P	$[M+H]^+$	1.58	2
**Alkaloids, Peptides & Nitrogenous**	Complex Polycyclic Alkaloid structure	18.83	441.26	C28 H32 N4 O	$[M+H]^+$	0.45	2
	Elaidamide	22.81	282.28	C18 H35 N O	$[M+H]^+$	0.38	2
	Chaetoglobosin K	18.73	555.29	C34 H40 N2 O5	$[M-H]^-$	0.33	2
	Bis-phenylpropanoid alkamide amide	21.44	445.23	C24 H32 N2 O6	$[M+H]^+$	0.31	2
	Complex Macrocyclic Peptide	21.26	482.29	C29 H39 N O5	$[M+H]^+$	0.27	2
	Chaetoglobosin K	18.97	555.29	C34 H40 N2 O5	$[M-H]^-$	0.24	2
	Venezueline G	18.63	194.08	C10 H13 N O3	$[M-H]^-$	0.20	2
	Medelamine A	19.19	214.25	C14 H31 N	$[M+H]^+$	0.19	2
	L-β-(3-hydroxyureido)-alanine	0.00	164.07	C4 H9 N3 O4	$[M+H]^+$	0.18	2
	Cyclic Peptolide	22.98	426.30	C27 H39 N O3	$[M+H]^+$	0.17	3
	Microsporin B	19.84	532.35	C28 H42 N4 O5	$[M+NH4]^+$	0.16	2
	Indolizidine Alkaloid derivative	17.57	240.23	C15 H29 N O	$[M+H]^+$	0.14	2
	Sphingofungin E	17.70	418.28	C21 H39 N O7	$[M+H]^+$	0.14	2
	Cyclic Peptolide	21.88	426.30	C27 H39 N O3	$[M+H]^+$	0.12	3
	Synechobactin B	17.76	531.30	C24 H44 N4 O9	$[M-H]^-$	0.12	2
	Cyclic Peptolide	24.59	426.30	C27 H39 N O3	$[M+H]^+$	0.10	3
**Volatiles & Small Organics**	Short-chain volatile ester	1.68	117.09	C6 H12 O2	$[M+H]^+$	0.34	3
	Oxalate-derived small organic cluster	0.24	123.04	C2 H6 N2 O4	$[M+H]^+$	0.56	3

		0.59	123.04	C2 H6 N2 O4	[M+H]+	0.33	3
		1.54	123.04	C2 H6 N2 O4	[M+H]+	0.10	3
**Phenolics & Polyketides**	Functionalized Indenone/Phenolic derivative	28.89	311.16	C20 H22 O3	[M+H]+	0.38	3
	Functionalized Indenone/Phenolic derivative	20.85	311.16	C20 H22 O3	[M+H]+	0.14	3
	Polyketide Macrolide	17.91	325.24	C19 H32 O4	[M+H]+	0.11	3
	Functionalized Indenone/Phenolic derivative	21.79	311.16	C20 H22 O3	[M+H]+	0.11	3
	Polyketide Macrocycle	25.46	485.29	C29 H42 O6	[M-H]-	0.12	2
**Exogenous Contaminants**	2-Dodecylbenzenesulfonic acid isomer (1-2)	19.51	325.19	C18 H30 O3 S	[M-H]-	1.58	2
		20.25	325.19	C18 H30 O3 S	[M-H]-	0.39	2
	4-Undecylbenzenesulfonic acid	19.09	311.17	C17 H28 O3 S	[M-H]-	0.31	2
	Acetyl tributyl citrate	24.10	403.23	C20 H34 O8	[M+H]+	0.16	2
	Cyclopentadecanolide	26.18	239.20	C15 H28 O2	[M-H]-	0.12	2
	Fatty acid derivative / Acetate artifact	24.82	251.20	C16 H28 O2	[M-H]-	0.27	3
	Ethyl elaidate	28.56	309.28	C20 H38 O2	[M-H]-	0.16	2

Table S6. ***Asparagopsis armata*** **Metabolomic Profile**

Family	Tentative Identification	RT (min)	m/z	Molecular Formula	Ionization Mode	Rel. Area (%)	MSI Level
**Core Fatty Acids & Aliphatic Lipids** These represent the essential lipid background and cell membrane building blocks of the alga, extracted primarily by the dichloromethane phase	Palmitic Acid	28.84	255.23	C16H32O2	$[M-H]^-$	6.88	2
	Oleic Acid	28.10	281.25	C18H34O2	$[M-H]^-$	5.62	2
	Palmitelaidic acid (isomer 1-3)	29.52	253.22	C16H30O2	$[M-H]^-$	0.93	2
		28.34	253.22	C16H30O2	$[M-H]^-$	0.64	2
		28.75	253.22	C16H30O2	$[M-H]^-$	0.63	2
	Myristic Acid	28.44	227.20	C14H28O2	$[M-H]^-$	0.61	2
	Arachidonic Acid (PUFA)	29.09	303.23	C20H32O2	$[M-H]^-$	0.76	2
	Eicosapentaenoic acid (EPA)	25.58	301.22	C20H30O2	$[M-H]^-$	0.14	2
	Docosapentaenoic acid (isomer 1-2)	25.07	331.26	C22H34O2	$[M+H]^+$	0.16	2
		28.26	331.26	C22H34O2	$[M+H]^+$	0.10	2
	Docosatrienoic acid derivative	29.04	352.32	C22H38O2	$[M+H]^+$	0.11	2
	Linolenic Acid	26.37	277.22	C18H30O2	$[M-H]^-$	0.12	2
	Aliphatic Lipid Ester	29.34	269.25	C17H34O2	$[M-H]^-$	0.23	3
	Glyceryl 2-myristate	28.86	301.24	C17H34O4	$[M-H]^-$	0.16	2
**Oxylipins & Oxidized Fatty Acid Derivatives** Highly enriched biological markers generated by the alga via enzymatic oxidation of core lipids as signaling molecules or defense responses	Dihydroxyhexadecanoic acid (isomer 1-4)	21.29	287.22	C16H32O4	$[M-H]^-$	2.93	3
		24.97	287.22	C16H32O4	$[M-H]^-$	0.41	3
		24.72	287.22	C16H32O4	$[M-H]^-$	0.17	3
		29.38	287.22	C16H32O4	$[M-H]^-$	0.10	3
	Oxidized C18 fatty acid (isomer 1-4)	24.98	313.24	C18H34O4	$[M-H]^-$	0.90	3
		23.71	313.24	C18H34O4	$[M-H]^-$	0.81	3
		24.72	313.24	C18H34O4	$[M-H]^-$	0.51	3
		29.38	313.24	C18H34O4	$[M-H]^-$	0.19	3
	13(S)-HPODE	21.01	311.22	C18H32O4	$[M-H]^-$	0.39	2
	Hydroxy-EPA	20.14	317.21	C20H30O3	$[M-H]^-$	0.23	2
	Hydroxy fatty acid (isomer 1-2)	25.26	271.23	C16H32O3	$[M-H]^-$	0.22	3
		27.55	271.23	C16H32O3	$[M-H]^-$	0.13	3
	Thapsic acid	21.21	285.21	C16H30O4	$[M-H]^-$	0.13	2

	Epoxystearic acid (isomer 1-2)	21.91	297.24	C18H34O3	$[M-H]^-$	0.13	2
		23.32	297.24	C18H34O3	$[M-H]^-$	0.11	2
	Glycerol ester derivative	21.04	299.22	C17H32O4	$[M-H]^-$	0.13	3
	Azelaic acid (Dicarboxylic acid)	10.69	187.10	C9H16O4	$[M-H]^-$	0.36	2
	12-HETE	21.28	319.23	C20H32O3	$[M-H]^-$	0.15	2
	Dihydroxyheptadecenyl acetate isomer	28.10	327.25	C19H36O4	$[M-H]^-$	0.14	3
	Dihydroxy-methyltetradecanoic acid	21.00	273.21	C15H30O4	$[M-H]^-$	0.15	3
	Modified aliphatic long-chain ketone	21.82	282.28	C18H32O	$[M+H]^+$	0.55	3
	Sphingoid polyol backbone derivative	25.30	319.28	C18H38O4	$[M+H]^+$	0.31	3
**Terpenoids, Carotenoid Fragments & Specialized Lipids** *Marine-derived isoprenoids, broken carotenoids, and highly specialized marine cyclic ethers/lactones surviving the non-volatile extraction protocol*	Meroterpenoid derivative (isomer 1-4)	25.06	425.27	C27H36O4	$[M+H]^+$	3.17	3
		22.78	425.27	C27H36O4	$[M+H]^+$	0.50	3
		16.77	425.27	C27H36O4	$[M+H]^+$	0.41	3
		22.00	425.27	C27H36O4	$[M+H]^+$	0.26	3
	Putative Diterpene (isomer 1-5)	25.07	271.24	C20H30	$[M+H]^+$	0.76	3
		28.25	271.24	C20H30	$[M+H]^+$	0.36	3
		24.71	271.24	C20H30	$[M+H]^+$	0.20	3
		20.70	271.24	C20H30	$[M+H]^+$	0.15	3
		29.42	271.24	C20H30	$[M+H]^+$	0.13	3
	Putative Sesquiterpene (isomer 1-2)	21.49	203.18	C15H22	$[M+H]^+$	0.32	3
		21.70	203.18	C15H22	$[M+H]^+$	0.30	3
		22.65	203.18	C15H22	$[M+H]^+$	0.11	3
	Oxygenated Sesquiterpenoid	23.82	219.17	C15H22O	$[M+H]^+$	0.11	3
	Dehydroretinaldehyde	25.04	283.21	C20H26O	$[M+H]^+$	0.11	2
	Dihydroretinol derivative	25.09	289.25	C20H32O	$[M+H]^+$	0.12	2
	Retinoid/Terpenoid ester derivative	27.87	327.23	C22H32O2	$[M-H]^-$	0.16	3
	Carotenoid breakdown product	23.26	583.41	C40H54O3	$[M+H]^+$	0.11	3
	Polyoxygenated Triterpenoid backbone	28.45	457.33	C29H46O4	$[M-H]^-$	0.11	3
	Sesterterpene/Polyketide lactone core	24.45	423.25	C27H34O4	$[M+H]^+$	0.15	3
	Marine bislactone/polyketide lipid (isomer 1-2)	21.09	381.27	C22H38O5	$[M-H]^-$	0.47	3
		21.28	381.27	C22H38O5	$[M-H]^-$	0.31	3

	Marine oxylipin/cyclic ether (isomer 1-3)	21.09	365.27	C22H36O4	$[M+H]^+$	0.35	3
		21.28	365.27	C22H36O4	$[M+H]^+$	0.33	3
		25.02	363.26	C22H36O4	$[M-H]^-$	0.16	3
	Dihydroactinidiolide	14.06	179.11	C11H16O2	$[M-H]^-$	0.23	2
**Nitrogenous Compounds, Amines & Nitrogen Primary Metabolites** *Highly water-soluble primary nitrogen pools and organic bases, eluting almost instantly at the beginning of reverse-phase chromatography*	Putative Alkylamine/Alkaloid fragment	1.62	128.14	C8H17N	$[M+H]^+$	1.93	3
	Polar aromatic amine fragment (isomer 1-3)	1.96	162.13	C11H15N	$[M+H]^+$	1.44	3
		8.85	162.13	C11H15N	$[M+H]^+$	0.17	3
		10.94	162.13	C11H15N	$[M+H]^+$	0.12	3
	Unknown Polar Primary Metabolite	1.73	122.10	C8H8	$[M+H]^+$	1.07	4
	Phenethylamine	1.65	122.10	C8H11N	$[M+H]^+$	1.07	3
	Aliphatic long-chain amine variant	19.20	214.25	C14H31N	$[M+H]^+$	0.77	3
	Piperidine alkaloid deri. (isomer 1-2)	10.93	220.17	C14H21NO	$[M+H]^+$	0.71	3
		9.47	220.17	C14H21NO	$[M+H]^+$	0.37	3
	Ultra-polar nitrogenous salt	1.80	123.04	C2H6N2O4	$[M+H]^+$	0.98	4
	Small aromatic amide derivative	9.16	192.14	C12H17NO	$[M+H]^+$	0.40	3
	Aliphatic amide segment	1.60	116.11	C6H13NO	$[M+H]^+$	0.36	3
	Polar nitrogenous acid core	18.62	194.08	C10H13NO3	$[M-H]^-$	0.36	3
	Piperidinyl alcohol derivative	1.66	158.15	C9H19NO	$[M+H]^+$	0.34	3
	Small aliphatic amide variant	1.77	172.17	C10H21NO	$[M+H]^+$	0.28	3
	Piperidinyl phenyl alcohol variant	10.26	206.15	C13H19NO	$[M+H]^+$	0.25	3
	Aminocaproic acid	1.51	132.10	C6H13NO2	$[M+H]^+$	0.25	2
	Palmitamide (Fatty amide isomer 1-2)	24.60	256.26	C16H33NO	$[M+H]^+$	0.14	2
		24.88	256.26	C16H33NO	$[M+H]^+$	0.12	2
	Hypoxanthine	1.49	137.05	C5H4N4O	$[M+H]^+$	0.18	2
	3-Formylindole	12.00	146.06	C9H7NO	$[M+H]^+$	0.18	2
	Guanine	1.50	152.06	C5H5N5O	$[M+H]^+$	0.13	2
	Indole-3-acetic acid	11.02	174.06	C10H9NO2	$[M-H]^-$	0.14	2
	Marine cyclic peptide fragment variant	25.67	482.36	C26H47N3O5	$[M+H]^+$	0.42	3
	Amide-linked phenolic siderophore core	29.35	431.18	C22H28N2O7	$[M-H]^-$	0.10	3

**Pigments & Chlorophyll Degradation Pathways** *Heavy, nitrogen-packed macrocycles representing the structural degradation and harvesting networks of photosynthetic red algal machinery*	Pheophorbide/Phorbin macrocycle (isomer 1-2)	24.88	593.27	C35H36N4O5	[M+H]+	2.37	2
		27.26	593.27	C35H36N4O5	[M+H]+	0.32	2
	Chlorophyll porphyrin catabolite (isomer 1-3)	29.77	609.27	C35H36N4O6	[M+H]+	0.34	2
		20.50	609.27	C35H36N4O6	[M+H]+	0.11	2
		29.45	609.27	C35H36N4O6	[M+H]+	0.10	2
	Phorbin derivative macrocycle	23.45	535.27	C33H34N4O3	[M+H]+	0.16	2
**Phenolics, Complex Glycosides & Aromatics** *Polyphenolic components, sugar conjugates, and defense structures generated from the core carbohydrate networks of red macroalgae.*	Highly oxygenated polyphenol (isomer 1-3)	24.74	509.07	C25H18O12	[M-H]-	3.07	3
		26.87	509.07	C25H18O12	[M-H]-	0.76	3
		25.52	509.07	C25H18O12	[M-H]-	0.39	3
	Polyphenolic/Flavonoid glucuronide (isomer 1-3)	24.74	507.08	C22H20O14	[M-H]-	1.56	3
		26.87	507.08	C22H20O14	[M-H]-	0.39	3
		25.52	507.08	C22H20O14	[M-H]-	0.20	3
	Iridoid glycoside (isomer 1-2)	23.71	421.13	C17H26O12	[M-H]-	0.26	3
		21.28	421.13	C17H26O12	[M-H]-	0.10	3
	Polyhydroxy aromatic derivative	21.69	403.12	C17H22O11	[M+H]+	0.12	3
	Alkylphenol derivative (isomer 1-2)	23.02	349.27	C22H36O3	[M+H]+	0.25	3
		23.27	349.27	C22H36O3	[M+H]+	0.24	3
**Proven Laboratory & Environmental Contaminants**	Saflufenacil (isomer 1-3)	24.74	499.05	C17H17ClF4N4O5S	[M-H]-	1.12	2
		26.87	499.05	C17H17ClF4N4O5S	[M-H]-	0.30	2
		25.52	499.05	C17H17ClF4N4O5S	[M-H]-	0.19	2
	Dodecylbenzenesulfonic acid (isomer 1-2)	19.55	325.19	C18H30O3S	[M-H]-	0.49	2
		20.21	325.19	C18H30O3S	[M-H]-	0.14	2
	Plastic additive (isomer 1-3)	23.71	429.17	C26H26N2O2S	[M-H]-	0.39	2
		26.19	429.17	C26H26N2O2S	[M-H]-	0.16	2
		21.28	429.17	C26H26N2O2S	[M-H]-	0.13	2
	Tetracycline	21.61	443.15	C22H24N2O8	[M-H]-	0.11	2
	Tianeptine	21.61	435.11	C21H25ClN2O4S	[M-H]-	0.11	2

Tributyl citrate acetate	24.09	403.23	$C_{20}H_{34}O_8$	$[M+H]^+$	0.26	2
Acetyl hexamethyl tetralin	24.35	259.21	$C_{18}H_{26}O$	$[M+H]^+$	0.19	2
Cyclohexanone (isomer 1-2)	1.50	94.02	$C_6H_{10}O$	$[M+H]^+$	0.36	2
	11.93	94.02	$C_6H_{10}O$	$[M+H]^+$	0.12	2
Tetrahydrothiophene	1.52	89.04	C_4H_8S	$[M+H]^+$	0.14	3
Short-chain volatile ester fragment	1.68	117.09	$C_6H_{12}O_2$	$[M+H]^+$	0.55	3
Linalyl propionate	16.55	228.20	$C_{13}H_{22}O_2$	$[M+H]^+$	0.31	3

Table S7. ***Polysiphonia elongata*** **Metabolomic Profile**

Family	Tentative Identification	RT [min]	m/z	Molecular Formula	Ionization Mode	Rel. Area (%)	MSI level
**Fatty Acids & Lipids**	Palmitic Acid	28.91	255.23	$C_{16}H_{32}O_2$	$[M-H]^-$	3.09	2
	Palmitelaidic acid	28.70	253.22	$C_{16}H_{30}O_2$	$[M-H]^-$	3.06	2
	Oleic acid	27.99	281.25	$C_{18}H_{34}O_2$	$[M-H]^-$	2.41	2
	Myristic acid	28.38	227.20	$C_{14}H_{28}O_2$	$[M-H]^-$	1.42	2
	Palmitelaidic acid	29.52	253.22	$C_{16}H_{30}O_2$	$[M-H]^-$	1.19	2
	Arachidonic acid	29.10	303.23	$C_{20}H_{32}O_2$	$[M-H]^-$	0.71	2
	2-[(5Z)-5-tetradecenyl]cyclobutanone	21.81	282.28	$C_{18}H_{32}O$	$[M+NH_4]^+$	0.42	2
	(2S,3S,4R)-1,2,3,4-Octadecanetetrol	25.30	319.28	$C_{18}H_{38}O_4$	$[M+H]^+$	0.37	2
	ethyl elaidate	27.70	309.28	$C_{20}H_{38}O_2$	$[M-H]^-$	0.33	2
	Eicosapentanoic acid (EPA) (Isomer 1-2)	25.57	301.22	$C_{20}H_{30}O_2$	$[M-H]^-$	0.28	2
		16.61	303.23	$C_{20}H_{30}O_2$	$[M+H]^+$	0.22	2
	16-Heptadecene-1,2,4-triol	27.25	285.24	$C_{17}H_{34}O_3$	$[M-H]^-$	0.27	2
	Juniperic acid (Isomer 1-2)	25.25	271.23	$C_{16}H_{32}O_3$	$[M-H]^-$	0.24	2
		21.50	271.23	$C_{16}H_{32}O_3$	$[M-H]^-$	0.11	2
	Pentadecanoic acid	21.57	241.22	$C_{15}H_{30}O_2$	$[M-H]^-$	0.23	2
	9Z-Heptadecenoic acid	21.42	267.23	$C_{17}H_{32}O_2$	$[M-H]^-$	0.21	2
	Linolenelaidic acid	26.35	277.22	$C_{18}H_{30}O_2$	$[M-H]^-$	0.20	2
	Azelaic acid (Isomer 1-2)	12.04	187.10	$C_9H_{16}O_4$	$[M-H]^-$	0.16	2
		10.71	187.10	$C_9H_{16}O_4$	$[M-H]^-$	0.11	2
	3-oxopalmitic acid	21.26	269.21	$C_{16}H_{30}O_3$	$[M-H]^-$	0.14	2
	Docosatetraenoic acid	25.47	331.27	$C_{22}H_{36}O_2$	$[M-H]^-$	0.13	2
	Isopropyl myristate	28.96	269.25	$C_{17}H_{34}O_2$	$[M-H]^-$	0.12	2
	Linolenelaidic acid	26.57	277.22	$C_{18}H_{30}O_2$	$[M-H]^-$	0.11	2
	Zuretinol acetate	27.89	327.23	$C_{22}H_{32}O_2$	$[M-H]^-$	0.11	2
	10,16-Dihydroxyhexadecanoic acid	21.69	287.22	$C_{16}H_{32}O_4$	$[M-H]^-$	0.11	2
	2-Hydroxymyristic acid	21.26	243.20	$C_{14}H_{28}O_3$	$[M-H]^-$	0.11	2
	(E)-parinaric acid	23.82	275.20	$C_{18}H_{28}O_2$	$[M-H]^-$	0.11	2

	1-Stearoyl-sn-glycero-3-phosphate (Isomer 1-2)	18.10	439.28	C21H43O7P	$[M+H]^+$	0.10	2
		20.50	439.28	C21H43O7P	$[M+H]^+$	0.10	2
**Algal Oxylipins & Epoxides** *These are oxidized lipids, heavily utilized by red algae as stress-signaling molecules and defense compounds against grazing,*	HEPE	17.10	319.23	C20H30O3	$[M+H]^+$	0.58	2
	9,10-EPOXYSTEARIC ACID (Isomer 1-4)	21.53	297.24	C18H34O3	$[M-H]^-$	0.67	2
		21.79	297.24	C18H34O3	$[M-H]^-$	0.37	2
		21.31	297.24	C18H34O3	$[M-H]^-$	0.21	2
		25.68	297.24	C18H34O3	$[M-H]^-$	0.15	2
	Algal Oxylipin	17.19	293.18	C17H26O4	$[M-H]^-$	0.20	2
	C20 Oxylipin derivative	17.05	331.19	C20H28O4	$[M-H]^-$	0.18	2
	9-KODE (Isomer 1-2)	21.43	293.21	C18H30O3	$[M-H]^-$	0.18	2
		21.20	293.21	C18H30O3	$[M-H]^-$	0.14	2
	9,10-epoxy-12-octadecenoic acid (Isomer 1-3)	22.72	295.23	C18H32O3	$[M-H]^-$	0.16	2
		22.33	295.23	C18H32O3	$[M-H]^-$	0.13	2
		20.59	295.23	C18H32O3	$[M-H]^-$	0.11	2
	12-HETE (Isomer 1-2)	21.64	319.23	C20H32O3	$[M-H]^-$	0.15	2
		21.28	319.23	C20H32O3	$[M-H]^-$	0.11	2
	resolvin E2	12.73	335.22	C20H30O4	$[M+H]^+$	0.12	2
**Terpenoids (Diterpenes, Meroterpenoids, & Derivatives)**	Meroterpenoid derivative (isomer 1-10)	24.44	423.25	C27H34O4	$[M+H]^+$	2.42	3
		21.14	423.25	C27H34O4	$[M+H]^+$	1.25	3
		19.71	423.25	C27H34O4	$[M+H]^+$	0.92	3
		21.43	423.25	C27H34O4	$[M+H]^+$	0.45	3
		18.45	423.25	C27H34O4	$[M+H]^+$	0.35	3
		26.63	423.25	C27H34O4	$[M+H]^+$	0.27	3
		22.60	423.25	C27H34O4	$[M+H]^+$	0.19	3
		27.05	423.25	C27H34O4	$[M+H]^+$	0.18	3
		26.20	423.25	C27H34O4	$[M+H]^+$	0.16	3
		20.76	423.25	C27H34O4	$[M+H]^+$	0.11	3
	Brevione A (Isomer 1-3)	24.30	425.27	C27H36O4	$[M+H]^+$	0.36	3
		23.85	442.29	C27H36O4	$[M+NH4]^+$	0.23	3
		23.45	442.29	C27H36O4	$[M+NH4]^+$	0.13	3

Oxygenated Diterpene (isomer 1-3)	12.77	317.21	C20H28O3	$[M+H]^+$	0.77	3
	18.30	317.21	C20H28O3	$[M+H]^+$	0.28	3
	16.20	317.21	C20H28O3	$[M+H]^+$	0.20	3
Mono-oxygenated Diterpene (isomer 1-2)	21.52	287.24	C20H30O	$[M+H]^+$	0.13	3
	16.61	285.22	C20H30O	$[M+H]^+$	0.12	3
Mono-oxygenated Diterpene (Isomer 1-2)	18.86	287.24	C20H28O	$[M+H]^+$	0.12	3
	19.94	285.22	C20H28O	$[M+H]^+$	0.10	3
Diterpene (Isomer 1-4)	17.29	271.24	C20H30	$[M+H]^+$	0.62	3
	21.03	271.24	C20H30	$[M+H]^+$	0.34	3
	22.18	271.24	C20H30	$[M+H]^+$	0.18	3
	26.07	271.24	C20H30	$[M+H]^+$	0.14	3
Diterpene (Isomer 1-4)	19.24	301.22	C20H28O2	$[M+H]^+$	0.24	3
	16.79	301.22	C20H28O2	$[M+H]^+$	0.18	3
	17.10	301.22	C20H28O2	$[M+H]^+$	0.13	3
	15.36	301.22	C20H28O2	$[M+H]^+$	0.10	3
Diterpene hydrocarbon (isomer 1-2)	24.81	273.26	C20H32	$[M+H]^+$	0.47	3
	21.56	273.26	C20H32	$[M+H]^+$	0.34	3
Diterpenoid (isomer 1-3)	15.19	297.18	C20H24O2	$[M+H]^+$	0.19	3
	16.61	297.18	C20H24O2	$[M+H]^+$	0.15	3
	15.96	297.18	C20H24O2	$[M+H]^+$	0.13	3
Putative Diterpenoid (Isomer 1-2)	21.61	329.21	C21H28O3	$[M+H]^+$	0.26	3
	16.28	327.20	C21H26O3	$[M+H]^+$	0.19	3
(4S,4aR,8aS)-4-[E)-2-3-(Furyl)vinyl]-3,4a,8,8-tetramethyl-4a,5,6,7,8,8a-(hexahydro-14H)-naphthalenone (isomer 1-5)	20.42	299.20	C20H26O2	$[M+H]^+$	0.67	3
	21.12	299.20	C20H26O2	$[M+H]^+$	0.52	3
	14.75	299.20	C20H26O2	$[M+H]^+$	0.18	3
	13.10	299.20	C20H26O2	$[M+H]^+$	0.18	3
	18.31	299.20	C20H26O2	$[M+H]^+$	0.15	3
Dehydroretinaldehyde (Isomer 1-2)	17.10	283.21	C20H26O	$[M+H]^+$	0.15	3
	19.24	283.21	C20H26O	$[M+H]^+$	0.14	3
(+)-Ingenol	13.51	347.19	C20H28O5	$[M-H]^-$	0.25	3

	4-OxoDictyoxetane	19.04	315.20	C20H26O3	[M+H]$^+$	0.24	3
	Loliolide	11.45	197.12	C11H16O3	[M+H]$^+$	0.13	3
	C13 apocarotenoid	16.53	228.20	C13H22O2	[M+NH4]$^+$	0.18	3
**Marine Alkaloids & Nitrogenous Compounds** *These highly specialized nitrogen-containing molecules represent defensive secondary metabolites and porphyrin photosynthetic) breakdown products*	Dictyolactam acetate	18.79	374.23	C22H31NO4	[M+H]$^+$	1.81	3
	Putative nitrogenous diterpene (Isomer 1-2)	21.87	296.20	C20H25NO	[M+H]$^+$	0.35	3
		23.44	296.20	C20H25NO	[M+H]$^+$	0.31	3
	Phorbinyl propanoic acid porphyrin (fragments)	24.78	593.28	C35H36N4O5	[M+H]$^+$	1.20	3
		27.22	593.28	C35H36N4O5	[M+H]$^+$	0.51	3
		24.25	593.27	C35H36N4O5	[M+H]$^+$	0.22	3
	Porphyrintriyl tripropanoic acid	20.46	609.27	C35H36N4O6	[M+H]$^+$	0.53	3
	Tetraazaheptacycloethanone (Isomer 1-4)	21.14	458.29	C28H32N4O	M+NH4]+	1.32	3
		24.49	441.26	C28H32N4O	[M+H]$^+$	0.33	3
		26.61	439.25	C28H32N4O	[M-H]$^-$	0.28	3
		19.71	458.29	C28H32N4O	M+NH4]+	0.23	3
	Asterriquinone J	21.47	587.23	C34H35ClN2O5	[M+H]$^+$	1.06	3
	Chaetoglobosin K	18.81	555.29	C34H40N2O5	[M-H]$^-$	0.57	3
	Medelamine A	19.14	214.25	C14H31N	[M+H]$^+$	0.37	3
	Kynurenic acid	17.17	190.05	C10H7NO3	[M+H]$^+$	0.34	3
	(+)-Fusarisetin A	13.28	390.23	C22H31NO5	[M+H]$^+$	0.31	3
	Aspochalasin O	24.40	458.29	C27H39NO5	[M+H]$^+$	0.24	3
	sphingoid base derivative	17.71	418.28	C21H39NO7	[M+H]$^+$	0.14	3
	sphingoid base derivative	21.67	532.35	C27H49NO9	[M+H]$^+$	0.11	3
	pseudouridine	17.17	245.08	C9H12N2O6	[M+H]$^+$	0.11	3
	(+)-Crocacin C	19.48	358.24	C22H31NO3	[M+H]$^+$	0.14	3
**Polyketides & Aromatics** *This family includes metabolites generated via the polyketide synthase PKS) pathway or containing complex aromatic ring systems, which are*	Putative fungal isocoumarin	17.17	177.05	C10H8O3	[M+H]$^+$	2.55	3
	(−)-Macrolactin-A	22.01	420.27	C24H34O5	[M+NH4]$^+$	0.64	3
	Parmosidone F	21.42	493.08	C25H18O11	[M-H]$^-$	0.63	3
	Putative glycosylated polyketide	21.42	491.08	C22H20O13	[M-H]$^-$	0.32	3
	(−)-Macrolactin-A	22.46	420.27	C24H34O5	[M+NH4]$^+$	0.12	3
	(−)-Centrolobine	14.43	313.18	C20H24O3	[M+H]$^+$	0.20	3

common secondary defensive traits across marine and associated microscopic organisms	Pironetin	19.95	325.24	C19H32O4	$[M+H]^+$	0.16	3
	Cylindrocyclophane C1	21.74	601.36	C36H55ClO5	$[M-H]^-$	0.10	3
**Miscellaneous Compounds & Elution/Surfactant Contaminants** *This section collects industrial plasticizers, processing solvents, linear alkylbenzene sulfonate detergents, and highly unique mixed-biosynthesis fragments that do not neatly fit into primary terpene or lipid classes,*	MBP Phthalate (fragment)	17.17	223.10	C12H14O4	$[M+H]^+$	0.84	3
	2-Dodecylbenzenesulfonic acid (Surfactant)	19.36	325.19	C18H30O3S	$[M-H]^-$	1.01	3
		20.10	325.19	C18H30O3S	$[M-H]^-$	0.27	3
	4-Undecylbenzenesulfonic acid (Surfactant)	18.98	311.17	C17H28O3S	$[M-H]^-$	0.21	3
	Butyl acetate Solvent (residue)	1.70	117.09	C6H12O2	$[M+H]^+$	0.33	3
	Dibutyl sebacate Plasticizer)	21.39	313.24	C18H34O4	$[M-H]^-$	0.16	3
	Tributyl citrate acetate Plasticizer)	24.09	403.23	C20H34O8	$[M+H]^+$	0.14	3
	[(1,2-Dioxo-1,2-ethanediyl)bis(oxy)]diammonium	0.25	123.04	C2H6N2O4	$[M+H]^+$	0.48	3
		1.28	123.04	C2H6N2O4	$[M+H]^+$	0.25	3
	Erinacine P	21.52	493.28	C27H40O8	$[M+H]^+$	0.20	3
	sinubrasolide A	24.13	439.28	C28H38O4	$[M+H]^+$	0.17	3
		23.37	455.28	C28H40O5	$[M-H]^-$	0.12	3
		20.50	474.32	C28H40O5	$[M+NH4]^+$	0.12	3

Table S8. ***Polysiphonia esp*****. Metabolomic Profile**

Metabolite Family	Tentative Identification	Rt (min)	m/z	Molecular Formula	Ionization Mode	Rel. Area (%)	MSI Level
**Fatty Acids & Small Oxygenated Lipids**	Oleic acid	28.31	281.25	C18H34O2	$[M-H]^-$	22.52	3
	Palmitic acid	29.11	255.23	C16H32O2	$[M-H]^-$	21.92	3
	Polyoxygenated polyketide	29.12	511.47	C32H64O4	$[M-H]^-$	0.69	4
	Hydroxy-octadecanoic acid	28.3	563.51	C36H68O4	$[M-H]^-$	0.61	4
	Nonadecanoic acid deriv.	28.27	327.25	C19H36O4	$[M-H]^-$	0.47	4
	Heptadecanoic acid deriv.	29.11	301.24	C17H34O4	$[M-H]^-$	0.47	4
	Palmitic acid isomer	26.64	255.23	C16H32O2	$[M-H]^-$	0.29	3
	Small organic acid	1.96	167.01	C11H2O2	$[M+H]^+$	0.29	4
	Docosahexaenoic acid (DHA)	25.89	331.27	C22H36O2	$[M-H]^-$	0.28	3
	Octacosatetraenoic acid deriv.	25.74	441.30	C28H42O4	$[M-H]^-$	0.27	4
	Docosapentaenoic acid deriv,	21.48	329.25	C22H34O2	$[M-H]^-$	0.22	3
	Eicosenoic acid	22.14	305.25	C20H34O2	$[M-H]^-$	0.21	3
	Hydroxy-octadecanoic acid	22.58	299.26	C18H36O3	$[M-H]^-$	0.19	3
	Heptadecenoic acid	21.87	267.23	C17H32O2	$[M-H]^-$	0.19	3
	Eicosenoic acid deriv,	24.14	403.23	C20H34O8	$[M+H]^+$	0.17	4
	Heneicosadienoic acid	22.21	317.25	C21H34O2	$[M-H]^-$	0.15	3
	Pentadecanoic acid	22.16	241.22	C15H30O2	$[M-H]^-$	0.14	3
	Small dicarboxylic acid	3.20	147.06	C6H10O4	$[M+H]^+$	0.11	3
	Polyoxygenated lipid	17.76	383.24	C21H34O6	$[M+H]^+$	0.11	4
	Oxygenated lipid	28.3	349.24	C21H34O4	$[M-H]^-$	0.11	4
	Heptadecenoic acid isomer	23.53	267.23	C17H32O2	$[M-H]^-$	0.11	3
	Eicosanoic acid / Arachidic deriv.	22.06	347.31	C20H42O4	$[M+H]^+$	0.10	3
	Diterpene / Hydrocarbon	17.89	271.24	C20H30	$[M+H]^+$	0.10	3
	Docosahexaenoic acid isomer	28.35	331.27	C22H36O2	$[M-H]^-$	0.10	3
	Hydroxy-eicosanoic acid	24.17	327.29	C20H40O3	$[M-H]^-$	0.10	3
	Eicosatrienoic acid	22.98	305.25	C20H34O2	$[M-H]^-$	0.10	3
	Linoleic acid	21.72	279.23	C18H32O2	$[M-H]^-$	0.09	3

	Hexadecenoic / Palmitoleic acid	22.52	253.22	C16H30O2	$[M-H]^-$	0.09	3
	Oleic acid isomer	26.85	281.25	C18H34O2	$[M-H]^-$	0.08	3
	Oxygenated FA	28.48	313.24	C18H34O4	$[M-H]^-$	0.05	4
	Linoleic acid isomer	19.94	279.23	C18H32O2	$[M-H]^-$	0.05	3
	Nonadecenoic acid	25.61	293.25	C19H34O2	$[M-H]^-$	0.04	3
	Oxygenated octadecanoic acid	23.56	303.29	C18H38O3	$[M+H]^+$	0.04	4
	Small oxygenated organic	1.86	139.02	C10H2O	$[M+H]^+$	0.04	4
	Oxygenated docosanoic acid	21.08	391.34	C22H46O5	$[M+H]^+$	0.04	4
	Hydroxy-palmitoleic acid	29.07	269.21	C16H30O3	$[M-H]^-$	0.04	4
	Palmitic acid isomer	24.98	255.23	C16H32O2	$[M-H]^-$	0.08	3
	Palmitic acid isomer	27.97	255.23	C16H32O2	$[M-H]^-$	0.07	3
	Palmitic acid isomer	24.09	255.23	C16H32O2	$[M-H]^-$	0.06	3
	Palmitic acid isomer	25.50	255.23	C16H32O2	$[M-H]^-$	0.05	3
	Oxylipin / Oxidized FA	17.24	293.18	C17H26O4	$[M-H]^-$	0.08	4
	Oxylipin	21.28	291.16	C17H24O4	$[M-H]^-$	0.05	4
	Small organic acid	1.65	219.09	C9H14O6	$[M+H]^+$	0.06	4
	Small aliphatic acid	12.10	187.10	C9H16O4	$[M-H]^-$	0.06	3
	Small aliphatic acid	13.01	171.10	C9H16O3	$[M-H]^-$	0.06	3
**Terpenoids, Carotenoids & Phenolics**	Carotenoid	24.01	641.42	C42H56O5	$[M+H]^+$	3.31	3
	Xanthophyll derivative	24.01	581.40	C40H52O3	$[M+H]^+$	2.05	3
	Phlorotannin	25.19	593.28	C34H40O9	$[M+H]^+$	1.4	4
	Carotenoid ester isomer	28.95	641.42	C42H56O5	$[M+H]^+$	1.35	3
	Complex Terpenoid	28.96	659.43	C42H58O6	$[M+H]^+$	0.95	4
	Xanthophyll derivative	28.47	581.40	C40H52O3	$[M+H]^+$	0.58	3
	Carotenoid ester isomer	28.48	641.42	C42H56O5	$[M+H]^+$	0.43	3
	Carotenoid ester isomer	23.11	641.42	C42H56O5	$[M+H]^+$	0.32	3
	Complex polyketide	23.09	681.41	C51H52O	$[M+H]^+$	0.29	4
	Carotenoid oxidation product	24.05	703.42	C43H60O8	$[M-H]^-$	0.20	4
	Xanthophyll derivative	22.31	581.40	C40H52O3	$[M+H]^+$	0.09	3
	Complex Terpenoid	28.82	657.42	C42H58O6	$[M-H]^-$	0.05	4

	Complex oxygenated lipid	28.32	585.49	C38H66O4	$[M-H]^-$	0.06	4
	Complex oxygenated lipid	29.11	533.46	C34H62O4	$[M-H]^-$	0.08	4
	Complex phenolic	29.09	587.23	C34H34O9	$[M+H]^+$	0.07	4
	Complex Terpenoid	24.74	563.47	C35H62O5	$[M+H]^+$	0.04	4
**Nitrogenous Metabolites**	Alkaloid	24.00	659.43	C37H58N2O8	$[M+H]^+$	4.56	4
	Oleamide	22.04	282.28	C18H35NO	$[M+H]^+$	1.81	3
	Peptide	28.95	581.40	C36H48N6O	$[M+H]^+$	1.69	4
	Tetradecylamine	19.26	214.25	C14H31N	$[M+H]^+$	1.34	3
	Small Dipeptide	0.29	123.04	C2H6N2O4	$[M+H]^+$	1.01	4
	Amino acid deriv,	0.29	151.04	C3H6N2O5	$[M+H]^+$	0.70	4
	Tyrosine deriv,	18.66	194.08	C10H13NO3	$[M-H]^-$	0.67	3
	Small Dipeptide	0.64	123.04	C2H6N2O4	$[M+H]^+$	0.62	4
	Complex Lipopeptide	28.87	681.41	C39H56N2O8	$[M+H]^+$	0.59	4
	Tridecylamine	17.31	200.24	C13H29N	$[M+H]^+$	0.57	3
	Amino acid deriv,	0.65	151.04	C3H6N2O5	$[M+H]^+$	0.55	4
	Tetradecylamine isomer	18.83	214.25	C14H31N	$[M+H]^+$	0.44	3
	Nitrogenous compound	24.04	502.34	C28H41N9	$[M-H]^-$	0.39	4
	Nitrogenous compound	20.83	609.27	C36H32N8O2	$[M+H]^+$	0.36	4
	Amino acid deriv,	0.29	164.07	C4H9N3O4	$[M+H]^+$	0.33	4
	Small Dipeptide	1.86	123.04	C2H6N2O4	$[M+H]^+$	0.32	4
	Fatty Amide	16.60	226.82	C13H25NO2	$[M+H]^+$	0.32	3
	Complex Peptide	22.36	572.38	C31H49N5O5	$[M+H]^+$	0.27	4
	Complex Peptide	17.72	423.23	C19H30N6O5	$[M+H]^+$	0.26	4
	Fatty Amide	17.75	418.28	C21H39NO7	$[M+H]^+$	0.26	4
	Palmitamide	24.92	256.26	C16H33NO	$[M+H]^+$	0.22	3
	Amino acid deriv,	0.29	170.04	C2H7N3O6	$[M+H]^+$	0.21	4
	Dipeptide / Polyamine	16.59	250.18	C10H23N3O4	$[M+H]^+$	0.21	4
	Complex Peptide	28.42	533.25	C33H32N4O3	$[M+H]^+$	0.18	4
	Complex Alkaloid / Peptide	23.08	581.40	C36H48N6O	$[M+H]^+$	0.17	4
	Amino acid derivative	0.65	170.04	C2H7N3O6	$[M+H]^+$	0.17	4

Amino acid derivative	0.63	164.07	C4H9N3O4	$[M+H]^+$	0.16	4
Small Nitrogenous	0.19	102.07	C3H7N3O	$[M+H]^+$	0.16	4
Docosanamide	25.75	338.34	C22H43NO	$[M+H]^+$	0.15	3
Complex Peptide	22.45	577.33	C27H48N2O11	$[M+H]^+$	0.15	4
Polyamine	21.05	408.37	C18H45N7O3	$[M+H]^+$	0.14	4
Nitrogenous organic	29.11	323.22	C15H28N6O2	$[M-H]^-$	0.14	4
Small N-heterocycle	0.33	208.04	C13H5NO2	$[M+H]^+$	0.13	4
Small Nitrogenous	1.88	102.07	C3H7N3O	$[M+H]^+$	0.13	4
Nitrogenous organic	28.3	210.11	C7H11N7O	$[M+H]^+$	0.12	4
Nitrogenous organic	29.17	210.11	C7H11N7O	$[M+H]^+$	0.12	4
Fatty Amide	20.21	452.39	C25H49N5O2	$[M+H]^+$	0.11	4
Amino acid / Pyrroline deriv,	25.44	172.13	C9H17NO2	$[M+H]^+$	0.10	4
Small Nitrogenous	0.29	165.06	C3H8N4O4	$[M+H]^+$	0.10	4
Docosanamide isomer	19.11	338.34	C22H43NO	$[M+H]^+$	0.09	3
Small Nitrogenous	0.32	121.04	C6H4N2O	$[M+H]^+$	0.09	4
Complex Peptide	28.47	584.42	C38H53N3O2	$[M+H]^+$	0.09	4
Nitrogenous lipid	22.19	416.35	C27H45NO2	$[M+H]^+$	0.08	4
Complex Peptide	21.60	635.51	C31H66N6O7	$[M+H]^+$	0.08	4
Small Nitrogenous	0.30	110.01	CH3NO5	$[M+H]^+$	0.08	4
Complex Alkaloid	28.96	533.25	C29H28N10O	$[M+H]^+$	0.08	4
Small Nitrogenous	0.32	140.01	C9HNO	$[M+H]^+$	0.07	4
Octylamine	0.56	130.16	C8H19N	$[M+H]^+$	0.07	3
Amino acid deriv,	25.43	170.12	C9H15NO2	$[M+H]^+$	0.07	4
Complex Lipopeptide	25.14	652.50	C34H69NO10	$[M+H]^+$	0.07	4
Complex Peptide	24.93	605.24	C35H32N4O6	$[M+H]^+$	0.07	4
Polyamine	19.27	496.42	C22H53N7O5	$[M+H]^+$	0.07	4
Small Nitrogenous	0.27	156.14	C9H17NO	$[M+H]^+$	0.07	4
Complex Polyamine	23.92	683.42	C24H58N8O14	$[M+H]^+$	0.07	4
Small Nitrogenous	0.64	165.06	C3H8N4O4	$[M+H]^+$	0.07	4
Complex Peptide	22.06	369.30	C18H36N6O2	$[M+H]^+$	0.07	4

	Complex Lipopeptide	23.86	740.55	C38H77NO12	$[M+H]^+$	0.06	4
	Nonylamine	16.6	158.15	C9H19NO	$[M+H]^+$	0.06	3
	Amino acid deriv,	24.36	170.12	C9H15NO2	$[M+H]^+$	0.06	4
	Small Nitrogenous	1.73	156.14	C9H17NO	$[M+H]^+$	0.06	4
	Complex Alkaloid	28.97	599.41	C41H50N4	$[M+H]^+$	0.06	4
	Complex Peptide	25.69	608.47	C33H61N5O5	$[M+H]^+$	0.06	4
	Complex Peptide	23.85	600.41	C38H53N3O3	$[M+H]^+$	0.05	4
	Small Nitrogenous	24.03	157.01	C2H2N6O3	$[M-H]^-$	0.05	4
	Hexadecanamide deriv,	13.15	274.27	C16H35NO2	$[M+H]^+$	0.05	4
	Amino acid deriv,	1.95	164.07	C4H9N3O4	$[M+H]^+$	0.05	4
	Small Peptide	12.14	269.14	C9H20N2O7	$[M+H]^+$	0.05	4
	Polyamine	21.08	459.49	C24H58N8	$[M+H]^+$	0.05	4
	Polyamine	22.33	578.34	C20H47N7O12	$[M+H]^+$	0.05	4
	Complex Peptide	26.51	584.42	C38H53N3O2	$[M+H]^+$	0.05	4
	Small Nitrogenous	0.93	139.98	CHNO7	$[M+H]^+$	0.04	4
	Fatty Amide	17.73	446.31	C23H43NO7	$[M+H]^+$	0.04	4
	Fatty Amide	15.17	226.18	C13H23NO2	$[M+H]^+$	0.04	4
	Complex Peptide	28.45	591.26	C35H34N4O5	$[M+H]^+$	0.04	4
	Fatty Amide	25.7	384.35	C23H45NO3	$[M+H]^+$	0.04	4
	Docosanamide deriv,	19.11	356.35	C22H45NO2	$[M+H]^+$	0.04	4
	Palmitamide isomer	29.37	256.26	C16H33NO	$[M+H]^+$	0.04	3
	Small Nitrogenous	0.11	162.11	C7H15NO3	$[M+H]^+$	0.04	4
**Sulfur-Containing Metabolites**	Small organic sulfate	0.32	167.01	C3H6N2O4S	$[M+H]^+$	1.37	3
	Alkyl sulfate	22.36	365.24	C18H38O5S	$[M-H]^-$	0.27	3
	Small S-amino acid deriv,	0.31	139.02	C2H6N2O3S	$[M+H]^+$	0.41	3
	N/S containing organic	0.32	186.02	C3H3N7OS	$[M+H]^+$	0.34	4
	Sulfolipid	21.85	339.20	C19H32O3S	$[M-H]^-$	0.20	4
	Sulfolipid	19.13	311.17	C17H28O3S	$[M-H]^-$	0.18	4
	Sulfolipid	18.29	311.17	C17H28O3S	$[M-H]^-$	0.08	4
	N/S compound	22.5	336.13	C18H17N5S	$[M+H]^+$	0.16	4

S-amino acid deriv,	0.32	185.02	C3H8N2O5S	$[M+H]^+$	0.15	4
Sulfolipid	20.28	325.19	C18H30O3S	$[M-H]^-$	0.14	4
N/S compound	0.8	171.99	C3HN5O2S	$[M+H]^+$	0.13	4
N/S compound	0.66	110.01	C2H7NS2	$[M+H]^+$	0.10	4
S-amino acid deriv,	0.29	153.03	C3H8N2O3S	$[M+H]^+$	0.10	4
N/S compound	1.98	171.99	C3HN5O2S	$[M+H]^+$	0.10	4
N/S compound	1.80	144.08	C7H13NS	$[M+H]^+$	0.10	4
Complex N/S compound	21.13	325.19	C11H30N6OS2	$[M-H]^-$	0.10	4
S-amino acid deriv,	0.64	148.95	C2H2N2O2S2	$[M-H]^-$	0.09	4
S-amino acid deriv,	1.87	186.02	C2H7N3O5S	$[M+H]^+$	0.08	4
Complex N/S compound	24.79	600.42	C29H61NO9S	$[M+H]^+$	0.08	4
N/S compound	18.22	265.15	C13H22N4S	$[M-H]^-$	0.08	4
N/S compound	3.20	274.12	C11H19N3O3S	$[M+H]^+$	0.08	4
S-amino acid deriv,	0.66	153.03	C3H8N2O3S	$[M+H]^+$	0.07	4
N/S compound	0.81	199.99	C4HN5O3S	$[M+H]^+$	0.07	4
N/S compound	1.66	274.12	C11H19N3O3S	$[M+H]^+$	0.07	4
N/S compound	1.95	199.99	C4HN5O3S	$[M+H]^+$	0.06	4
Complex N/S compound	25.74	406.33	C21H47N3S2	$[M+H]^+$	0.06	4
Complex N/S compound	29.12	496.30	C22H47N3O7S	$[M-H]^-$	0.06	4
S-compound	0.32	181.01	C9H8S2	$[M+H]^+$	0.06	4
Complex N/S compound	24.15	583.40	C23H54N10O5S	$[M+H]^+$	0.06	4
N/S compound	0.28	195.99	C5HN5O2S	$[M+H]^+$	0.05	4
N/S compound	0.34	255.94	C2HN5O6S2	$[M+H]^+$	0.05	4
Complex N/S compound	28.28	496.30	C26H47N3O2S2	$[M-H]^-$	0.05	4
S-compound	24.02	201.04	C12H10OS	$[M-H]^-$	0.05	4
Alkyl sulfate	17.46	265.15	C12H26O4S	$[M-H]^-$	0.04	3
N/S compound	20.75	293.18	C15H26N4S	$[M-H]^-$	0.04	4
N/S compound	24.01	411.27	C22H38N2O3S	$[M+H]^+$	0.04	4
Complex S-compound	28.31	589.24	C38H36O4S	$[M+H]^+$	0.04	4
Chlorinated lipid	29.19	492.31	C25H49Cl2N3O2	$[M-H]^-$	0.24	4

**Halogenated Compounds Cl, Br)**	Chlorinated lipid isomer	28.31	492.31	C25H49Cl2N3O2	$[M-H]^-$	0.20	4
	Chlorinated peptide/alkaloid	23.83	693.39	C39H51ClN10	$[M-H]^-$	0.18	4
	Small Chlorinated S-cmpd	0.29	169.05	C6H13ClOS	$[M+H]^+$	0.13	4
	Small Chlorinated S-cmpd	0.64	169.05	C6H13ClOS	$[M+H]^+$	0.09	4
	Complex Chlorinated N/S	24.05	546.37	C29H58ClN3S2	$[M-H]^-$	0.10	4
	Chlorinated N-compound	29.13	313.19	C14H27ClN6	$[M-H]^-$	0.10	4
	Small Chlorinated N-cmpd	0.30	280.99	C6H5ClN4O7	$[M+H]^+$	0.10	4
	Chlorinated fatty acid	28.30	317.23	C18H35ClO2	$[M-H]^-$	0.09	4
	Chlorinated N-compound	28.36	339.21	C16H29ClN6	$[M-H]^-$	0.09	4
	Small Chlorinated N-cmpd	0.65	141.01	C2H5ClN2O3	$[M+H]^+$	0.08	4
	Chlorinated lipid	28.80	377.27	C22H44Cl2	$[M-H]^-$	0.08	4
	Brominated N-lipid	29.13	712.57	C41H84BrN3O	$[M-H]^-$	0.07	4
	Chlorinated N-lipid	24.06	541.35	C29H47ClN8	$[M-H]^-$	0.07	4
	Chlorinated S-lipid	29.12	369.24	C22H39ClS	$[M-H]^-$	0.07	4
	Chlorinated Pigment/Lipid	28.80	693.39	C42H59ClO6	$[M-H]^-$	0.07	4
	Chlorinated N/S cmpd	0.29	192.06	C8H14ClNS	$[M+H]^+$	0.07	4
	Chlorinated N-compound	0.30	239.97	C4H2ClN3O7	$[M+H]^+$	0.06	4
	Chlorinated N-compound	0.29	265.02	C15H5ClN2O	$[M+H]^+$	0.06	4
	Chlorinated N-compound	0.63	182.03	C4H8ClN3O3	$[M+H]^+$	0.06	4
	Complex Chlorinated N-lipid	28.33	738.59	C38H83Cl2N7O2	$[M-H]^-$	0.06	4
	Chlorinated fatty amide	17.87	330.22	C18H32ClNO2	$[M+H]^+$	0.1	4
	Chlorinated fatty amide	17.53	330.22	C18H32ClNO2	$[M+H]^+$	0.05	4
	Chlorinated N-lipid	29.09	351.22	C20H33ClN2O	$[M-H]^-$	0.05	4
	Chlorinated lipid	28.28	613.52	C39H76Cl2	$[M-H]^-$	0.05	4

Supplementary Material about Flow Cytometry. Figures

<table>
<tr><td>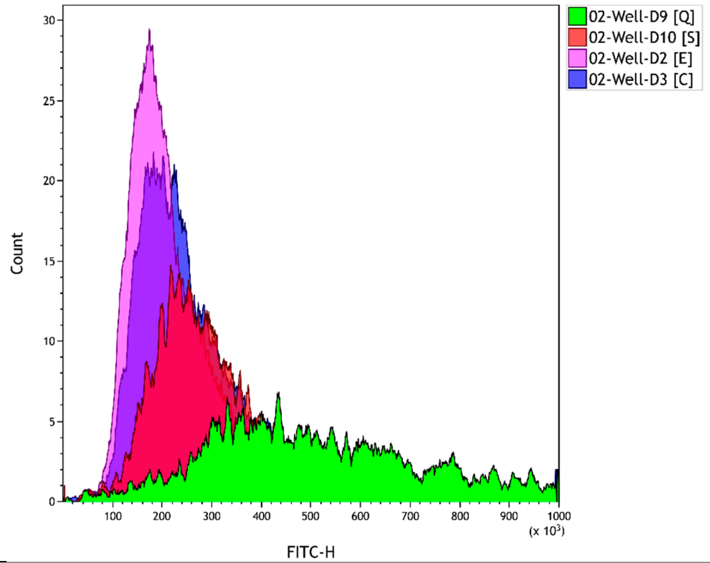
</td><td></td></tr>
<tr><td>Reactive oxygen species (ROS): Plot Overlay. D9: ARO; D10: ADD; D2: Control; D3: Control-Acetone</td><td></td></tr>
</table>

Supplementary Figure S1. Representative overlay histogram of intracellular reactive oxygen species (ROS) in control and macroalgal extract-treated HT-29 cells.

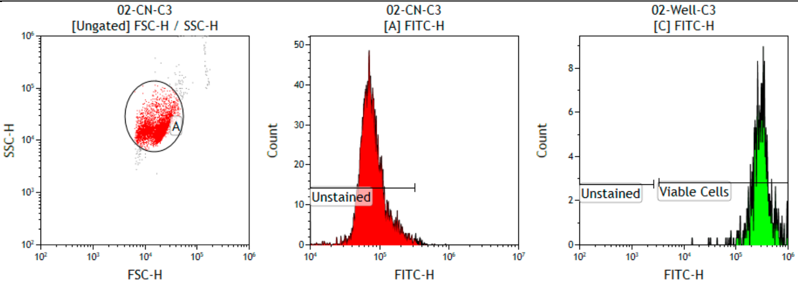

Gate	%Gated
All	100.00
Unstained	0.00
Viable Cells	98.81

Control Culture – Viability (Fluorescein diacetate)

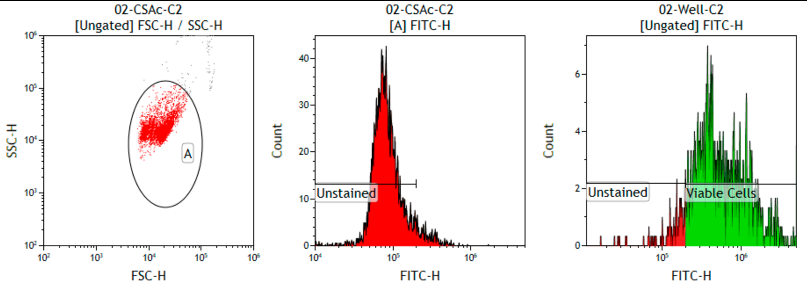

Gate	%Gated
All	100.00
Unstained	8.75
Viable Cells	91.51

Control Culture with Acetone – Viability (Fluorescein diacetate)

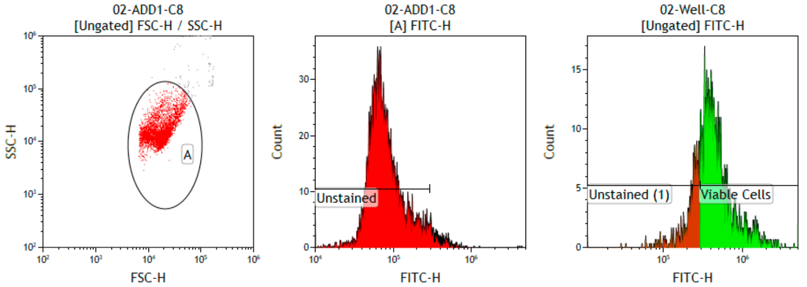

Gate	%Gated
All	100.00
Unstained (1)	23.72
Viable Cells	77.00

Experiment Culture (ADD) – Viability (Fluorescein diacetate)

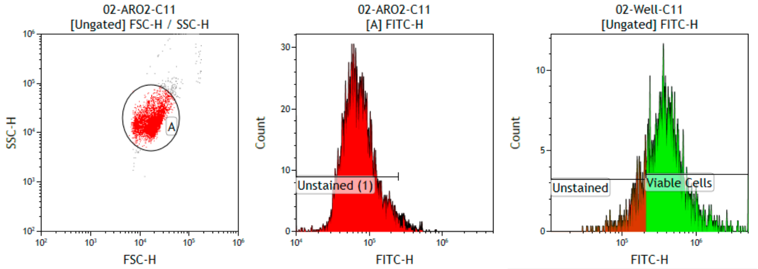

Gate	%Gated
All	100.00
Unstained	18.94
Viable Cells	82.72

Experiment Culture (ARO) – Viability (Fluorescein diacetate)

Supplementary Figure S2. Representative FDA fluorescence histograms showing metabolic viability of HT-29 cells treated with Dictyota dichotoma (ADD) and Rugulopteryx okamurae (ARO) extracts.

APOPTOSIS-NECROSIS

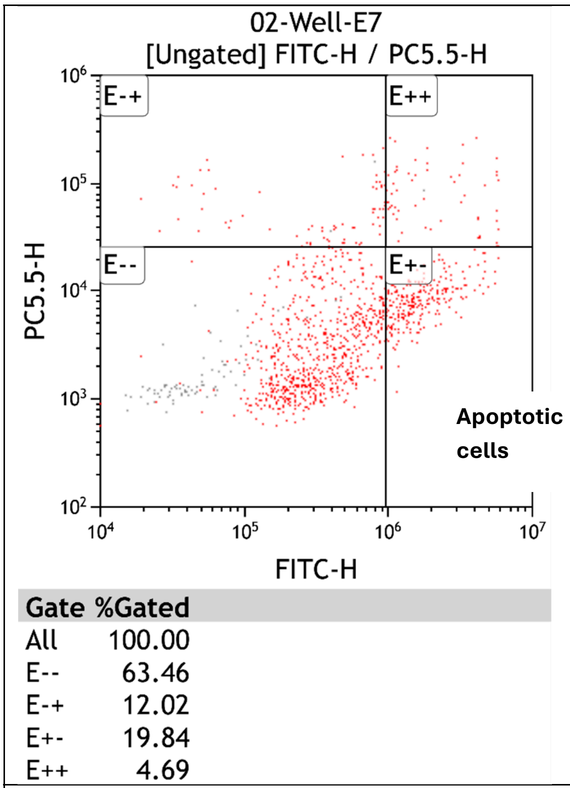

Control Culture (positive for apoptosis)– Apoptosis (Annexin V-FITC + PI)

Supplementary Figure S3. Positive apoptosis control validating Annexin V-FITC/PI staining in HT-29 cells.

Table S9. Quantified Population Breakdown Across Replicates (n = 3)

Population Quadrant	Control (%)	Control-Ac (%)	ADD Treatment (%)	ARO Treatment (%)	p-value (vs. Control-Ac)
**Viable** (E--)	91.5 ± 0.8	92.3 ± 1.1	92.0 ± 1.4	93.8 ± 0.9	> 0.05 (ns)
**Early Apoptosis** (E+-)	0.50 ± 0.12	0.32 ± 0.08	0.17 ± 0.05	0.36 ± 0.09	> 0.05 (ns)
**Late Apoptosis** (E++)	1.81 ± 0.25	1.59 ± 0.19	0.84 ± 0.14	1.14 ± 0.21	> 0.05 (ns)
**Necrosis / Damage** (E-+)	6.21 ± 0.74	5.76 ± 0.65	7.03 ± 0.82	4.67 ± 0.58	> 0.05 (ns)

Data expressed as mean ± standard deviation across three independent biological replicates (n = 3). Statistical significance evaluated via one-way ANOVA followed by Tukey's post-hoc test; ns = non-significant (p > 0.05).
